# Parameter estimation in models of biological oscillators: an automated regularised estimation approach

**DOI:** 10.1186/s12859-019-2630-y

**Published:** 2019-02-15

**Authors:** Jake Alan Pitt, Julio R. Banga

**Affiliations:** 1 (Bio)Process Engineering Group, IIM-CSIC, Eduardo Cabello 6, Vigo, 36208 Spain; 2 RWTH Aachen University, Faculty of Medicine, Joint Research Centre for Computational Biomedicine (JRC-COMBINE), Aachen, Germany

**Keywords:** Parameter estimation, Global optimisation, Regularisation, Parameter bounding, Dynamic modelling

## Abstract

**Background:**

Dynamic modelling is a core element in the systems biology approach to understanding complex biosystems. Here, we consider the problem of parameter estimation in models of biological oscillators described by deterministic nonlinear differential equations. These problems can be extremely challenging due to several common pitfalls: (i) a lack of prior knowledge about parameters (i.e. massive search spaces), (ii) convergence to local optima (due to multimodality of the cost function), (iii) overfitting (fitting the noise instead of the signal) and (iv) a lack of identifiability. As a consequence, the use of standard estimation methods (such as gradient-based local ones) will often result in wrong solutions. Overfitting can be particularly problematic, since it produces very good calibrations, giving the impression of an excellent result. However, overfitted models exhibit poor predictive power.

Here, we present a novel automated approach to overcome these pitfalls. Its workflow makes use of two sequential optimisation steps incorporating three key algorithms: (1) sampling strategies to systematically tighten the parameter bounds reducing the search space, (2) efficient global optimisation to avoid convergence to local solutions, (3) an advanced regularisation technique to fight overfitting. In addition, this workflow incorporates tests for structural and practical identifiability.

**Results:**

We successfully evaluate this novel approach considering four difficult case studies regarding the calibration of well-known biological oscillators (Goodwin, FitzHugh–Nagumo, Repressilator and a metabolic oscillator). In contrast, we show how local gradient-based approaches, even if used in multi-start fashion, are unable to avoid the above-mentioned pitfalls.

**Conclusions:**

Our approach results in more efficient estimations (thanks to the bounding strategy) which are able to escape convergence to local optima (thanks to the global optimisation approach). Further, the use of regularisation allows us to avoid overfitting, resulting in more generalisable calibrated models (i.e. models with greater predictive power).

**Electronic supplementary material:**

The online version of this article (10.1186/s12859-019-2630-y) contains supplementary material, which is available to authorized users.

## Background

Oscillations and sustained rhythms are pervasive in biological systems and have been deeply studied in areas such as metabolism [1–5], the cell cycle [6–9], and Circadian rhythms [10–16], to name but a few. In recent years, many research efforts have been devoted to the development of synthetic oscillators [17–20], including tunable ones [21–24].

Mathematical and computational approaches have been widely used to explore and analyse these complex dynamics [25–32]. Model-based approaches have also allowed for the identification of design principles underlying circadian clocks [12, 33] and different types of biochemical [34] and genetic oscillators [35–39]. Similarly, the study of the behaviour of populations of coupled oscillators has also greatly benefited from mathematical analysis and computer simulations [40–47].

A number of approaches can be used to build mathematical models of these biological oscillators [48–52]. This process is sometimes called reverse engineering, inverse problem solving or dynamic model identification [53–55]. Model calibration (i.e. parameter estimation or data fitting) is a particularly important and challenging sub-problem in the identification process [56–64]. Different strategies have been specially developed and applied to calibrate models of oscillators [32, 50, 65–71] and to characterise and explore their parameter space [31, 72–76].

In this study, we consider parameter estimation in mechanistic dynamic models of biological oscillators. From all the issues that plague model calibration [77], we pay special attention to three that are particularly problematic in oscillatory models: huge search spaces, multimodality and overfitting. We also discuss how to handle lack of identifiability.

## Methods

### Models of biological oscillators

Here, we consider mechanistic models of oscillatory biological systems given by deterministic nonlinear ordinary differential (ODEs). The general model structure is:

1 $$\begin{array}{*{20}l} \frac{d\mathbf{x}(t, \boldsymbol{\theta})}{dt} &= \mathbf{f}\left(t, \mathbf{u}(t),\mathbf{x}\left(t,\boldsymbol{\theta}\right), \boldsymbol{\theta}\right), \text{ for } \mathbf{x}(t, \boldsymbol{\theta}) \in \mathbf{\Psi}_{O}  \end{array} $$

2 $$\begin{array}{*{20}l} \mathbf{y}\left(\mathbf{x},\boldsymbol{\theta}\right) &= \mathbf{g}\left(\mathbf{x}\left(\boldsymbol{\theta}, t\right), t, \boldsymbol{\theta}\right) \end{array} $$

3 $$\begin{array}{*{20}l} \mathbf{x}\left(t_{0}, \boldsymbol{\theta}\right) &= \mathbf{x}_{0}  \end{array} $$

where $\mathbf {x} \in \mathbb {R}^{N_{\mathbf {x}}}$ represents the states of the system as time-dependent variables, under the initial conditions **x**_0_, $\boldsymbol {\theta } \in \mathbb {R}^{N_{\boldsymbol {\theta }}}$ is the parameter vector, **u**(*t*) represents any time-dependent input (e.g. stimuli) affecting the system and $t \in \left [t_{0}, t_{end}\right ] \subset \mathbb {R}$ is the time variable. **Ψ**_*O*_ represents the set of all possible oscillatory dynamics. The observation function $g : \mathbb {R}^{N_{\mathbf {x}}\times N_{\boldsymbol {\theta }}}\mapsto \mathbb {R}^{N_{\mathbf {y}}}$ maps the states to a vector of observables $\mathbf {y}\in \mathbb {R}^{N_{\mathbf {y}}}$, i.e. the state variables that can be measured. While the methodology here is developed for and tested on oscillatory models, it is not strictly restricted to models that exhibit such behaviour.

### Formulation of the parameter estimation problem

We now consider the parameter estimation problem considering dynamic models described by the above Eqs. (1 – 3). We formulate this estimation problem as a maximisation of the likelihood function given by:

4 $$ L\left(\tilde{\mathbf{y}}|\boldsymbol{\theta}\right) = \prod_{k = 1}^{N_{e}}\prod_{j = 1}^{N_{y, k}}\prod_{i=1}^{N_{t, k, j}}\frac{1}{\sqrt[]{2\pi\sigma_{kji}^{2}}}e^{\left(\frac{-\left(y_{kji}\left(\mathbf{x}\left(t_{i}, \boldsymbol{\theta}\right), \boldsymbol{\theta}\right) - \tilde{y}_{kji}\right)^{2}}{2\sigma_{kji}^{2}}\right)}  $$

where *N*_*e*_ is the number of experiments, *N*_*y*,*k*_ the number of observables in those experiments, *N*_*t*,*k*,*j*_ is the number of time points for each observable, $\tilde {y}_{kji}$ represents the measured value for the i^th^ time point of the j^th^ observable in the k^th^ experiment, and *σ*_*kji*_ represents its corresponding standard deviation. Under specific conditions [78], the maximisation of the likelihood formulation is equivalent to the minimisation of the weighted least squares cost given by:

5 $$\begin{array}{*{20}l} Q_{NLS}(\boldsymbol{\theta}) \,=\, \sum_{k = 1}^{N_{e}}\sum_{j = 1}^{N_{y, k}}\sum_{i = 1}^{N_{t, k, j}}\left(\frac{y_{kji}\left(\mathbf{x}\left(t_{i}, \boldsymbol{\theta}\right), \boldsymbol{\theta}\right) - \tilde{y}_{kji}}{\sigma_{kji}}\right)^{2} \,=\, \mathbf{r}(\boldsymbol{\theta})^{T}\mathbf{r}(\boldsymbol{\theta}) \end{array} $$

Using the above cost, the estimation problem can be formulated as the following minimisation problem:

6 $$ \min_{\boldsymbol{\theta}}\left(Q_{NLS}(\boldsymbol{\theta})\right) = \min_{\boldsymbol{\theta}}\left(\mathbf{r}(\boldsymbol{\theta})^{T}\mathbf{r}(\boldsymbol{\theta})\right)  $$

subject to the dynamic system described by Eqs. (1–3), and also subject to the parameter bounds:

7 $$ \theta_{i}^{min} \leq \theta_{i} \leq \theta_{i}^{max} \: \forall\: \theta_{i} \in \boldsymbol{\theta}  $$

We denote the solution to this minimisation problem as $\widehat {\boldsymbol {\theta }}$. In principle, this problem could be solved by standard local optimisation methods such as Gauss-Newton or Levenberg-Marquardt. However, as described next, there are many pitfalls and issues that complicate the application of these methods to many real problems.

### Pitfalls and perils in the parameter estimation problem

Numerical data fitting in nonlinear dynamic models is a hard problem with a long list of possible pitfalls, including [77]: a lack of identifiability, local solutions, badly scaled data and parameters, oscillating dynamics, inconsistent constraints, non-differentiable model functions, slow convergence and errors in experimental data. It should be noted that several of these difficulties are interrelated, e.g. a lack of practical identifiability can be due to noisy and non-informative data and will result in slow convergence and/or finding local solutions.

In the case of oscillators, the above issues apply, particularly multimodality and lack of identifiability. However, there are at least two additional important difficulties that must be also considered: overfitting (i.e. fitting the noise rather than the signal) and very large search spaces (which creates convergence difficulties and also makes it more likely the existence of additional local optima). Although these four issues are all sources of difficulties for proper parameter estimation, the last two have been less studied.

#### Lack of identifiability

The objective of identifiability analysis is to find out whether it is possible to uniquely estimate the values of the unknown model parameters [79]. It is useful to distinguish between two types of identifiability: structural and practical. Structural identifiability [80, 81] studies if the model parameters can be uniquely determined assuming ideal conditions for the measurements and therefore only considering the model dynamics and the input-output mapping (i.e. what is perturbed and what is observed). Structural identifiability is sometimes called a priori identifiability. Despite recent advances [82–84], structural identifiability analysis remains difficult to apply to large dynamic models with arbitrary nonlinearities.

It is important to note that, even if structural identifiability holds, unique determination of parameter values is not guaranteed since it is a necessary condition but not a sufficient one. Practical identifiability analysis [85–87] considers experimental limitations, i.e. it aims to find if parameter values can be determined with sufficient precision taking into account the limitations in the measurements (i.e. the amount and quality of information in the observed data). Practical (sometimes called a posteriori) identifiability analysis will typically compute confidence intervals of the parameter values. Importantly, it can also be taken into account as an objective in optimal experimental design [86].

#### Multimodality

Schittkowski [77] puts emphasis on the extremely difficult nature of data fitting problems when oscillatory dynamics are present: the cost function to be minimised will have a large number of local solutions and an irregular structure. If local optimisation methods are used, they will likely converge to one of these local solutions (typically the one with the basin of attraction that includes the initial guess). Several researchers have studied the landscape of the cost functions being minimised, describing them as very rugged and with multiple local minima [77, 88, 89]. Thus, this class of problems clearly needs to be solved with some sort of global optimisation scheme as illustrated in a number of studies during the last two decades [57, 86, 90–93].

The simplest global optimisation approach (and widely used in parameter estimation) is the so-called multi-start method, i.e. a (potentially large) number of repeated local searchers initialised from usually random initial points inside the feasible space of parameters. Although a number of studies have illustrated the power of this approach [94–96], others have found that it can be inefficient [92, 97–99]. This is especially the case when there is a large number of local solutions: in such situations, the same local optima will be repeatedly found by many local searches, degrading efficiency.

Thus, several methods have tried to improve the performance of the plain multi-start method by incorporating mechanisms to avoid repeated convergence to already found local solutions. This is the case of hybrid metaheuristics, where a global search phase is performed via diversification mechanisms and combined with local searches (intensification mechanisms). In this context, the enhanced scatter search (eSS) method has shown excellent performance [64, 97, 99, 100]. Here we will use an extension of the eSS method distributed in the MEIGO toolbox [101] as the central element of our automated multi-step approach. We have modified the MEIGO implementation of eSS in several ways, as detailed in Additional file 1. In order to illustrate the performance and robustness of eSS with respect to several state-of-the-art local and global solvers, we provide a critical comparison in Additional file 2.

#### Huge search spaces

In this study, we consider the common case scenario where little prior information about (some or all of the) parameters is available and therefore we need to consider ample bounds in the parameter estimation. These huge parameter bounds complicate convergence from arbitrary initial points, increase computation time and make it more likely that we will have a large number of local solutions in the search space. Although deterministic methods, which could be used to systematically reduce these bounds exist [102–104], currently they do not scale up well with problem size. Such techniques therefore can not be applied to problems of realistic size. Some analytical approaches have also been used for the analysis of biological oscillators [26, 31]. Alternatively, non-deterministic sampling techniques have been used to explore the parameter space and identify promising regions consistent with pre-specified dynamics [105]. Inspired by these results, we will re-use the sampling performed during an initial optimisation phase to reduce the parameter bounds.

#### Overfitting

Overfitting describes the problem associated with fitting the noise in the data, rather than the signal. Overfitted models can be misleading as they present a low-cost function value, giving the false impression that they are well-calibrated models that can be useful for making predictions. However, overfitted models have poor predictive power, i.e. they do not generalise well and can result in major prediction artefacts [106]. In order to fight overfitting, a number of regularisation techniques have been presented. Regularisation methods originated in the area of inverse problem theory [107]. Most regularisation schemes are based on adding a penalty term to the cost function, based on some prior knowledge of the parameters. This penalty makes the problem more regular, in the sense of reducing ill-conditioning and by penalising wild behaviour. Regularisation also can be used to minimise model complexity.

However, regularisation methods for nonlinear dynamics models remain an open question [99]. Further, these methods require some prior knowledge about the parameters and a tuning process which can be cumbersome and computationally demanding. Here, we will present a workflow that aims to automate this process.

#### Small illustrative example

In order to graphically illustrate several of the above issues, let us consider the ENSO problem, a small yet challenging example taken from the National Institute of Standards (NIST) nonlinear least squares (NLLS) test suite [108].

**Fig. 1:**
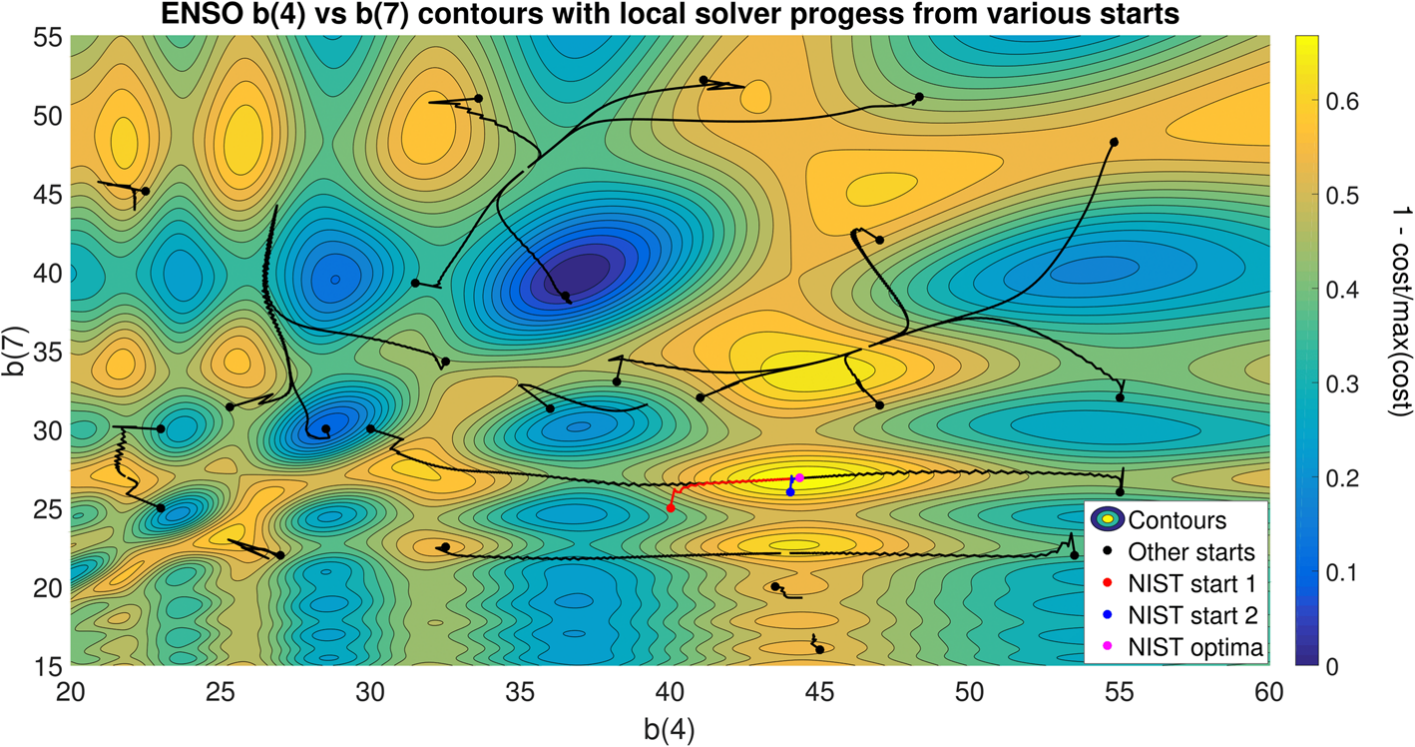
ENSO problem NL2SOL contours: contours of the cost function (nonlinear least squares) for parameters b4 and b7, and trajectories of a multi-start of the NL2SOL local solver

To visualise the multimodality of this problem, we can use contour plots of the cost function for pairs of parameters, as shown in Fig. 1. In this figure we also show the convergence paths followed by a multi-start of a local optimisation method (NL2SOL [109]), illustrating how most of the runs converge to local solutions or saddle points close to the initial point. We can also see how different runs converge to the same local solutions, explaining the low efficiency of multi-start for problems with many local optima. We also provide additional figures for this problem in Additional file 1.

**Fig. 2:**
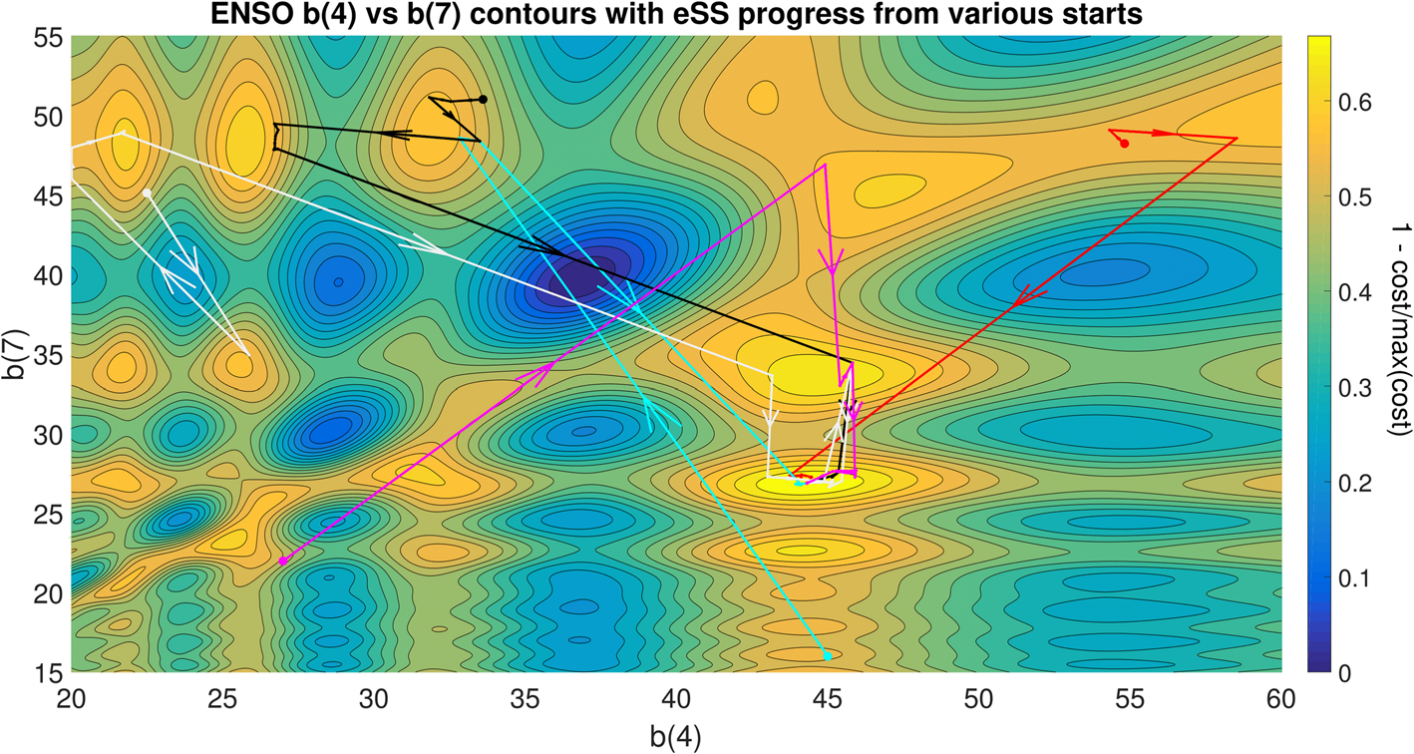
ENSO problem eSS contours: contours of the cost function (nonlinear least squares) for parameters b4 and b7, and trajectories of the enhanced scatter search (eSS) global optimisation solver initialized from various starting points

In contrast, we plot the convergence paths of the enhanced scatter search (eSS) method in Fig. 2, showing how the more efficient handling of the local minima allows this strategy to successfully and consistently find the global solution even from initial guesses that are far from the solution. It should be noted that while NIST lists this ENSO problem as being of “average” difficulty, this is largely due to the excellent starting point considered in their test suite, which are extremely close to the solution. Indeed, we can see in the aforementioned figures that the choice of parameter bounds and initial guess can dramatically change the difficulty of the problem.

### An automated regularised estimation approach

Here we present a novel methodology, GEARS (Global parameter Estimation with Automated Regularisation via Sampling), that aims to surmount the pitfalls described above. Our method combines three main strategies: i) global optimisation, (ii) reduction of the search space and (iii) regularised parameter estimation. In addition to these strategies, the method also incorporates identifiability analysis, both structural and practical. All these strategies are combined in a hands-off procedure, requiring no user supervision after the initial information is provided.

**Fig. 3:**
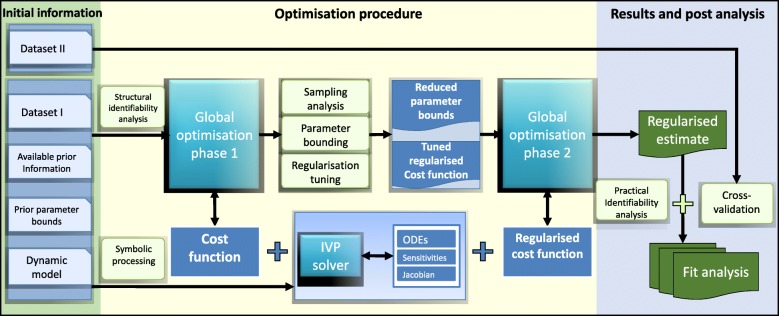
Workflow of procedure: a schematic diagram of the GEARS method

An overview of the entire procedure can be seen in Fig. 3. The initial information required by the method includes the dynamic model to be fitted (as a set of ODEs), the input-output mapping (including the observation function) and a data set for the fitting (dataset I). A second data set is also needed for the purposes of cross-validation and evaluation of overfitting (dataset II). Additionally, users can include (although it is not mandatory) any prior information about the parameters and their bounds. If the latter is not available, users can just declare very ample bounds, since the method is prepared for this worst-case scenario.

The method first performs two pre-processing steps. The first is a structural identifiability analysis test. A second pre-processing step involves symbolic manipulation to generate the components needed for the efficient computation of parametric sensitivities. After the pre-processing steps, the method performs a first global optimisation run using eSS and a non-regularised cost function. This step is used to obtain useful sampling information about the cost function landscape, which is then used to perform parameter bounding and regularisation tuning. This new information is then fed into a second global optimisation run, again using eSS but now with a regularised cost function and the new (reduced) parameter bounds. The outcome of this second optimisation is the regularised estimate, which is then subject to several post-fit analysis, including practical identifiability and cross-validation (using dataset II). Details regarding each of these steps are given below.

#### Structural identifiability analysis

The structural identifiability analysis step allows us to ensure that based on the model input-output (observation) mapping we are considering, we should in principle be able to uniquely identify the parameter values of the model (note that it is a necessary but not sufficient condition). If the problem is found to be structurally non-identifiable, users should take appropriate actions, like model reformulation, model reduction or by changing the input-output mapping if possible.

In our workflow, we analyse the structural identifiability of the model using the STRIKE-GOLDD package [82], which tests identifiability based on the rank of the symbolic Lie derivatives of the observation function. It can then detect each structurally non-identifiable parameter based on rank deficiency.

#### Symbolic processing for efficient numerics

In GEARS we use a single-shooting approach, i.e. the initial value problem (IVP) is solved for each valuation of the cost function inside the iterative optimisation loop. It is well known that gradient-based local methods, such as those used in eSS, require high-quality gradient information.

Solving the IVP (original, or extended for sensitivities) is the most computationally expensive part of the optimisation, so it is important to use efficient IVP solvers. In GEARS we use AMICI [110], a high level wrapper for the CVODES solver [111], currently regarded as the state of the art. In order to obtain the necessary elements for the IVP solution, the model is first processed symbolically by AMICI, including the calculation of the Jacobian. It should be noted that an additional advantage of using AMICI is that allows the integration of models with discontinuities (including events and logical operations).

#### Global optimisation phase 1

The objective of this step is to perform an efficient sampling (storing all the points tested during the optimisation) of the parameter space. This sampling will then be used to perform (i) reduction of parameter bounds, and (ii) tuning of the regularisation term to be used in the second optimisation phase.

The cost function used is a weighted least-squares criterion as given by Eqs. (5–7). The estimation problem is solved using the enhanced scatter search solver (eSS, [112]), implemented in the MEIGO toolbox [101]. Within the eSS method, we use the gradient-based local solver NL2SOL [109]. In order to maximise its efficiency, we directly provide the solver with sensitivities calculated using AMICI. After convergence, eSS finds the optimal parameters vector for the fitting data $\widehat {\boldsymbol {\theta }}^{I}$. While this solution might fit the dataset I very well, it is rather likely that it will not have the highest predictive power (as overfitting may have occurred). During the optimization, we store each for function evaluation the parameter vector $\boldsymbol {\theta }^{S}_{i}$ and its cost value $Q_{NLS}\left (\boldsymbol {\theta }^{S}_{i}\right) = \zeta _{i}$, building the sampling:

8 $$\begin{array}{*{20}l} \boldsymbol{\Theta} &= \left[\boldsymbol{\theta}^{S}_{1}, \dots, \boldsymbol{\theta}^{S}_{N_{S}}\right]\in \mathbb{R}^{N_{\boldsymbol{\theta}}}\times\mathbb{R}^{N_{S}}  \end{array} $$

9 $$\begin{array}{*{20}l} \boldsymbol{\zeta} &\,=\, \left[{Q}_{NLS}\left(\boldsymbol{\theta}^{S}_{1}\right), \dots, {Q}_{NLS}\left(\boldsymbol{\theta}^{S}_{N_{S}}\right)\right] \,=\, \left[\zeta_{1} \dots \zeta_{N_{S}}\right]\!\in\! \mathbb{R}^{N_{S}}  \end{array} $$

where *N*_*S*_ is the number of function evaluations, *N*_***θ***_ is the number of parameters and each $Q_{NLS}\left (\boldsymbol {\theta }^{S}_{i}\right)$ is a parameter vector selected by eSS.

#### Parameter bounding

The sampling obtained in the global optimisation phase 1 is now used to reduce the bounds of the parameters, making the subsequent global optimisation phase 2 more efficient and less prone to the issues detailed above. We first compute calculate a cost cut-off value for each parameter using Algorithm 1. This algorithm is used to determine reasonable costs, whereby costs deemed to be far from the global optimum are rejected. We calculate one cost cut off for each parameter, as different parameters have different relationships to the cost function. Once these cut-off values have been calculated for each parameter, we apply Algorithm 2 to obtain the reduced bounds.


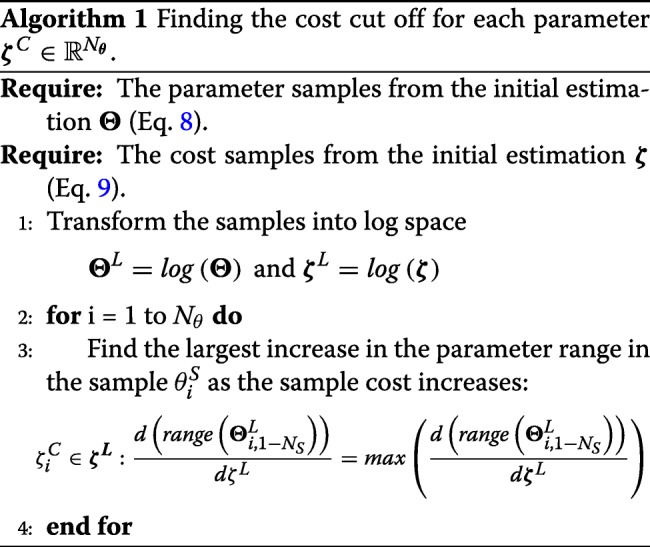



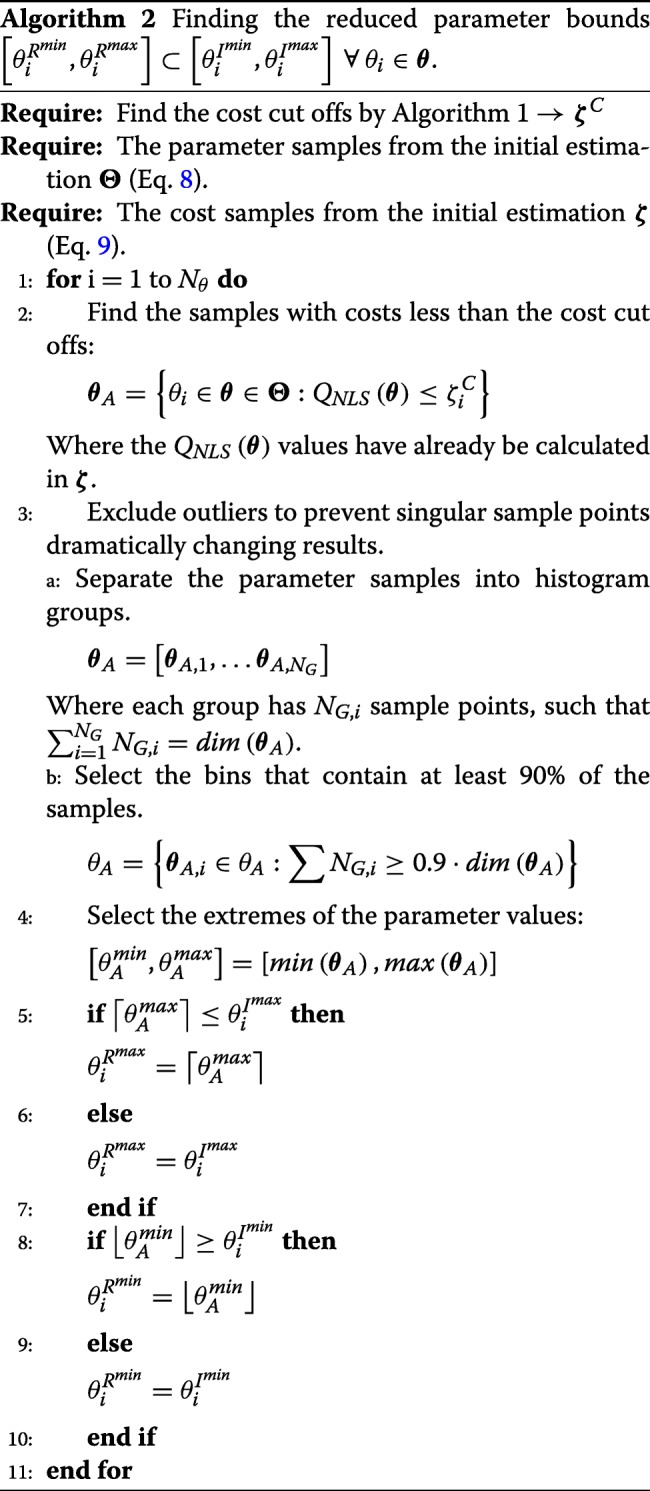


#### Regularised cost function

The next step builds an extended cost function using a Tikhonov-based regularisation term. This is a two norm regularisation term given by:

10 $$\begin{array}{*{20}l} \Gamma(\boldsymbol{\theta})&= \left(\boldsymbol{\theta} - \boldsymbol{\theta}^{ref} \right)^{T}\mathbf{W}^{T}\mathbf{W}\left(\boldsymbol{\theta} - \boldsymbol{\theta}^{ref} \right)  \end{array} $$

11 $$\begin{array}{*{20}l} \mathbf{W} &= \left[ \begin{array}{ccccc} \frac{1}{\theta^{ref}_{1}} & 0 & \cdots & \cdots & 0 \\ 0 & \frac{1}{\theta^{ref}_{2}} & 0 & \cdots & 0 \\ \vdots & 0 & \ddots & \ddots & \vdots \\ \vdots & \vdots & \ddots & \ddots & 0 \\ 0 & 0 & \cdots & 0 & \frac{1}{\theta^{ref}_{N_{\theta}}} \end{array}\right]  \end{array} $$

where *W* normalises the regularisation term with respect to ***θ***^*ref*^, to avoid bias due to the scaling of the reference parameter. The extended cost function is as follows:

12 $$ Q_{R}(\boldsymbol{\theta}) = Q_{NLS}(\boldsymbol{\theta}) + \alpha\Gamma(\boldsymbol{\theta})  $$

where *α* is a weighting parameter regulating the influence of the regularisation term.

Once the regularised cost function is built, we need to tune the regularisation parameters. Once again, we start from the cost cut off values calculated in Algorithm 2. We also use the reduced parameter bounds to ensure that our regularisation parameters and reduced parameter bounds do not conflict each other. The procedure for calculating the values for the regularisation parameters *α* and ***θ***^*ref*^ can be found in Algorithm 3.


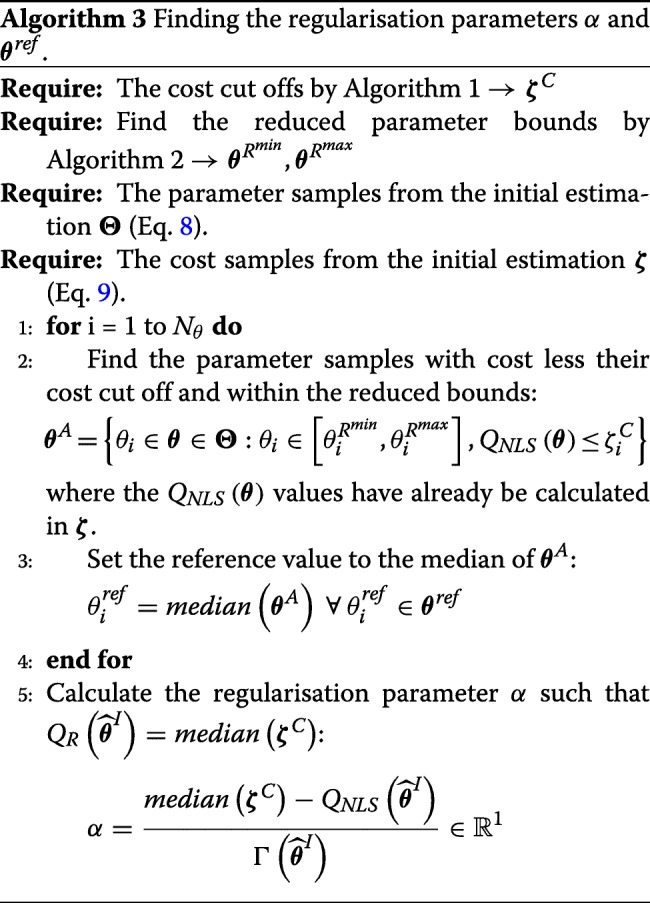


#### Global optimisation phase 2

Once we have calculated both the values of the regularisation parameters and the reduced parameter bounds, we are now able to perform the final regularised optimisation. We use the same set up for the global optimisation solver (eSS with NL2SOL as the local solver, and AMICI as the IVP solver). We then solve the regularised parameter estimation problem given by:

13 $$ \min_{\boldsymbol{\theta}} Q_{R}(\boldsymbol{\theta}) = \min_{\boldsymbol{\theta}}\left(Q_{NLS}(\boldsymbol{\theta}) + \alpha\Gamma(\boldsymbol{\theta})\right)  $$

Subject to the system described in Eqs. 1–3, and the reduced parameter bounds given by:

14 $$ {\theta}_{i}^{R^{min}} \leq \theta_{i} \leq {\theta}_{i}^{R^{max}} \: \forall\: \theta_{i} \in \boldsymbol{\theta}  $$

We denote the solution to this regularised estimation problem as $\widehat {\boldsymbol {\theta }}^{R}$.

### Practical identifiability analysis

The next step is to analyse the practical identifiability of the regularised solution. This is done using an improved version of the VisId toolbox [87]. The VisId toolbox accesses practical identifiability based on testing collinearity between parameters. A lack of practical identifiability is typically due to a lack of information in the available fitting data, and in principle can be surmounted by a more suitable experimental design [86].

#### Cross-validation and post-fit analysis

Next, we assess the level of overfitting using cross-validation. This is typically done by comparing the fitted model predictions with experimental data obtained under conditions (e.g. initial conditions) different from the ones used in the fitting dataset. In other words, cross-validation tests how generalisable the model is. Here, we perform cross-validation on both the non-regularised $\widehat {\boldsymbol {\theta }}^{I}$ and regularised solution $\widehat {\boldsymbol {\theta }}^{R}$. This allows us to assess the reduction of overfitting due to the regularised estimation.

In addition to cross-validation, several post-fit statistical metrics are also computed: normalised root mean square error (NRMSE), *R*^2^ and *χ*^2^ tests [113], parameter uncertainty (confidence intervals computed using the Fisher information matrix, FIM), and parameter correlation matrix (also computed using the FIM). The normalised root mean square error is a convenient metric for the quality of fit, given by:

15 $$\begin{array}{*{20}l} NRMSE\left(\boldsymbol{\theta}\right) &= \sqrt[]{\frac{\sum\limits_{k = 1}^{N_{e}}\sum\limits_{j = 1}^{N_{y, k}}\sum\limits_{i = 1}^{N_{t, k, j}}\left(\frac{y_{kji} - \tilde{y}_{kji}}{\max{\left(\tilde{\mathbf{y}}_{kj}\right)} - \min{\left(\tilde{\mathbf{y}}_{kj}\right)}}\right)^{2}}{n}} \end{array} $$

We use the NRMSE measure to assess both the quality of fit and quality of prediction. One important caveat to note here is that some of these post-fit analyses are based on the Fisher information matrix (FIM) for their calculation. This is a first order approximation and can be inaccurate for highly nonlinear models [114]. In those instances, bootstrapping techniques are better alternatives, although they are computationally expensive.

#### Implementation: The GEARS Matlab toolbox

The methodology proposed here has been implemented in a Matlab toolbox, “GEARS: Global parameter Estimation with Automated Regularisation via Sampling”. GEARS is a software package that can be downloaded from https://japitt.github.io/GEARS/, made freely available under the terms of the GNU general public license version 3. GEARS runs on Matlab R2015b or later and is multi-platform (tested on both Windows and Linux). The Optimisation and Symbolic Math Matlab toolboxes are required to run GEARS. In addition to this, GEARS requires the freely available AMICI package (http://icb-dcm.github.io/AMICI/) to solve the initial value problem. Optionally, it also requires the Ghostscript software (https://www.ghostscript.com) for improved exportation of results. For more details please see the documentation within the GEARS toolbox. It should be noted that for the structural and practical identifiability analysis steps, users need to install the VisId [87] and STRIKE-GOLDD [82] packages respectively. These packages are freely available at https://github.com/gabora/visid and https://sites.google.com/site/strikegolddtoolbox/ respectively.

### Case studies

Next, we consider four case studies of parameter estimation in dynamic models of biological oscillators. The general characteristics of these problems are given in Table 1. While these problems are small in terms the number of parameters, they exhibit most of the difficult issues discussed above, such as overfitting and multimodality, making them rather challenging. For each case study, synthetic datasets (i.e. pseudo-experimental data generated by simulation from a set of nominal parameters) were generated. For each case study, 10 fitting datasets with equivalent initial conditions were produced, plus a set of 10 additional cross-validation data sets (where the initial conditions were changed randomly within a reasonable range). All these data sets were generated using a standard deviation of 10.0% of the nominal signal value and a detection threshold of 0.1.

**Table 1:** Summary of case studies considered

	FitzHugh-Nagumo	Goodwin Oscillator	Repressilator	Enzymatic Oscillator
Abbreviation	FHN	GO	RP	EO
Main Reference	[117]	[25]	[69]	[116]
Number of parameters	3	8	4	7
Number of estimated parameters	3	8	4	7
Number of states	2	3	6	3
Number of observables	1	2	1	2
Number of data points per experiment	6	20	20	14

#### FitzHugh-Nagumo (FHN) problem

This problem considers the calibration of a FitzHugh-Nagumo model, which is a simplified version of the Hodgkin-Huxley model [115], describing the activation and deactivation dynamics of a spiking neuron. We consider the FHN model as described by Eqs. 16–21:

16 $$\begin{array}{*{20}l} \frac{dV}{dt} &= g\left(V - \frac{V^{3}}{3} + R\right)  \end{array} $$

17 $$\begin{array}{*{20}l} \frac{dR}{dt} &= -\frac{1}{g}\left(V - a + b\cdot R\right) \end{array} $$

18 $$\begin{array}{*{20}l} V(t_{0},\boldsymbol{\theta}) &= V_{0} \end{array} $$

19 $$\begin{array}{*{20}l} R(t_{0},\boldsymbol{\theta}) &= R_{0} \end{array} $$

20 $$\begin{array}{*{20}l} y(t_{i}) &= V(t_{i}) \end{array} $$

21 $$\begin{array}{*{20}l} \boldsymbol{\theta} &= \{a, b, g\} \in \left[10^{-5}, 10^{5}\right] \end{array} $$

where *y* is the observation function considered in the example. The flexibility of the model dynamics makes this model prone to overfitting. Synthetic data was generated taking nominal parameter values {*a*,*b*,*g*}={0.2,0.2,3}. The fitting data was generated with initial conditions of *V*_0_=−1,*R*_0_=1.

#### Goodwin (GO) oscillator problem

The Goodwin oscillator model describes control of enzyme synthesis by feedback repression. The GO model is capable of oscillatory behaviour in particular areas of the parameter space. Griffith [26] showed that limit-cycle oscillations can be obtained only for values of the Hill coefficient *n*≥8 (note that this information could be used to bound this parameter but here we will not use it, assuming a worst case scenario with little prior information available). The GO model suffers from some identifiability issues as well as tendency to overfitting. The dynamics are given by Eqs. 22–28:

22 $$\begin{array}{*{20}l} \frac{{dx}_{1}}{dt} &= \frac{k_{1} \cdot K_{i}^{n}}{K_{i}^{n} + x_{3}^{n}} - k_{2} \cdot x_{1}  \end{array} $$

23 $$\begin{array}{*{20}l} \frac{{dx}_{2}}{dt} &= k_{3}\cdot x_{1} - k_{4} \cdot x_{2} \end{array} $$

24 $$\begin{array}{*{20}l} \frac{{dx}_{3}}{dt} &= k_{5}\cdot x_{2} - k_{6}\cdot x_{3} \end{array} $$

25 $$\begin{array}{*{20}l} x_{1-3}(t_{0},\boldsymbol{\theta}) &= x_{1-3, 0} \end{array} $$

26 $$\begin{array}{*{20}l} \mathbf{y}_{F}(t_{i}) &= \left[x_{1}(t_{i}), x_{3}(t_{i})\right] \end{array} $$

27 $$\begin{array}{*{20}l} \mathbf{y}_{V}(t_{i}) &= \left[x_{1}(t_{i}), x_{2}(t_{i}), x_{3}(t_{i})\right] \end{array} $$

28 $$\begin{array}{*{20}l} \boldsymbol{\theta} &\,=\, \{k_{1-6}, K_{i}, n\} \!\text{ where } \{k_{1-6}, K_{i}\} \\ &\quad\!\in\! \left[10^{-3}, 10^{3}\right] \!\text{ and }\!n\! \in \left[1, 12\right] \end{array} $$

where variables {*x*_1_,*x*_2_,*x*_3_} represent concentrations of gene mRNA, the corresponding protein, and a transcriptional inhibitor, respectively; **y**_*F*_ is the observation function for the estimation problem, and **y**_*V*_ is the observation function for the cross-validation procedure. Synthetic data was generated considering nominal parameter values {*k*_1−6_,*K*_*i*_,*n*}={1,0.1,1,0.1,1,0.1,1,10}. The fitting datasets were generated for the initial conditions *x*_1−3,0_= [ 0.1,0.2,2.5]. It is important to note that we have considered an additional observable for cross-validation, which makes the problem much more challenging (i.e. it exacerbates the prediction problems due to overfitting).

#### Repressilator (RP) problem

The Repressilator is a well-known synthetic gene regulatory network [17]. We consider the particular parameter estimation formulation studied by [69] with dynamics given by Eqs. 29–37:

29 $$\begin{array}{*{20}l} \frac{{dp}_{1}}{dt} &= \beta(m_{1} - p_{1})  \end{array} $$

30 $$\begin{array}{*{20}l} \frac{{dp}_{2}}{dt} &= \beta(m_{2} - p_{2}) \end{array} $$

31 $$\begin{array}{*{20}l} \frac{{dp}_{3}}{dt} &= \beta(m_{3} - p_{3}) \end{array} $$

32 $$\begin{array}{*{20}l} \frac{{dm}_{1}}{dt} &= \alpha_{0} + \frac{\alpha}{\left(1+p_{3}^{n}\right)} - m_{1} \end{array} $$

33 $$\begin{array}{*{20}l} \frac{{dm}_{2}}{dt} &= \alpha_{0} + \frac{\alpha}{\left(1+p_{1}^{n}\right)} - m_{2} \end{array} $$

34 $$\begin{array}{*{20}l} \frac{{dm}_{3}}{dt} &= \alpha_{0} + \frac{\alpha}{\left(1+p_{2}^{n}\right)} - m_{3} \end{array} $$

35 $$\begin{array}{*{20}l} p_{1-3}(t_{0}) &= p_{1-3, 0}, m_{1-3}(t_{0}) = m_{1-3, 0} \end{array} $$

36 $$\begin{array}{*{20}l} \boldmath{y}_{F}(t_{i}) &= m_{3}(t_{i}) \text{ and} \boldmath{V}(t_{i}) = \left[p_{3}(t_{i}), m_{3}(t_{i})\right] \end{array} $$

37 $$\begin{array}{*{20}l} \boldsymbol{\theta} &\,=\, \{\alpha_{0}, \alpha, \beta, n\} \text{ where } \{\alpha_{0}, \alpha, \beta\}\\ &\quad\in \left[10^{-3}, 500\right] \text{ and } n \in \left[1, 10\right] \end{array} $$

where **y**_*F*_ is the observation function for the fitting procedure and *y*_*V*_ is the observation function for the cross-validation procedure (i.e. an additional observable for cross-validation). Synthetic data was generated considering nominal parameter values {*k*_1−6_,*K*_*i*_,*n*}= [ 0.05,298,8.5,0.3]. The fitting data was generated for the initial conditions given by [*p*_1−3,0_,*m*_1−3,0_]= [ 10,0.01,1,1,0.01,10].

#### Enzymatic oscillator (EO) problem

The enzymatic oscillator is a small biochemical system model that illustrates the effect of coupling between two instability-generating mechanisms [116]. Parameter estimation in this system is particularly challenging due to the existence of a variety of modes of dynamic behaviour, from simple periodic oscillations to birhythmicity and chaotic behaviour. The chaotic behaviour is restricted to a particular region of the parameter space as discussed in [116]. Its dynamics are difficult even for regions with simple periodic behaviour: the existence of extremely steep oscillations causes small shifts in the period of oscillations to have a large impact on the estimation cost function. We consider the dynamics described by Eqs. 38–43:

38 $$\begin{array}{*{20}l} \frac{d\alpha}{dt} &= v_{Km1_{r1}} - \frac{\alpha\cdot\sigma_{r2}(\alpha + 1)(\beta + 1)^{2}}{10^{6}L1_{r2} + (\alpha + 1)^{2}(\beta + 1)^{2}} \end{array} $$

39 $$\begin{array}{*{20}l} \frac{d\beta}{dt} &= \frac{50\alpha\cdot\sigma_{r2}(\alpha + 1)(\beta + 1)^{2}}{\left(10^{6}L1_{r2} + (\alpha + 1)^{2}\left(\beta + 1\right)^{2}\right)}\\ &\quad- \frac{\sigma_{2_{r3}}(\gamma + 1)^{2}\left(d_{r3}\frac{\beta}{100} + 1\right)\beta}{\left(L2_{r3} + (\gamma + 1)^{2}\left(d_{r3}\frac{\beta}{100} + 1\right)^{2}\right)} \end{array} $$

40 $$\begin{array}{*{20}l} \frac{d\gamma}{dt} &= \frac{\left(\!\sigma_{2_{r3}}(\gamma + 1)^{2}\left(d_{r3}\frac{\beta}{100} + 1\right)\!\beta\!\right)}{\left(\!50\left(L2_{r3} + (\!\gamma + 1)^{2}\left(d_{r3}\frac{\beta}{100} \!+ \!1\!\right)^{2}\!\right)\!\right)} \,-\, {ks}_{r4}\!\cdot\!\gamma \end{array} $$

41 $$\begin{array}{*{20}l} \alpha(t_{0}) &= \alpha_{0}, \beta(t_{0}) = \beta_{0}, \gamma(t_{0}) = \gamma_{0} \end{array} $$

42 $$\begin{array}{*{20}l} \mathbf{y}(t_{i}) &= \left[\alpha(t_{i}), \beta(t_{i})\right] \end{array} $$

43 $$\begin{array}{*{20}l} \boldsymbol{\theta} &= \left\{\!v_{Km1_{r1}} L1_{r2},\sigma_{r2},\! L2_{r3},d_{r3},\!\sigma_{2_{r3}}, \!{ks}_{r4}\!\right\}\!\in\! \left[\!10^{-3}\!, \!10^{3}\right]  \end{array} $$

where **y** is the observation function. Synthetic data was generated considering nominal parameter values ***θ***= [ 0.4,500,10,10,0.07,7,2.5]. An important point to note here is that these parameter values were chosen to be in the vicinity of, but not inside, the region with chaotic behaviour. For the fitting datasets the initial conditions [*α*_0_,*β*_0_,*γ*_0_]= [ 29.1999,188.8,0.3367] were used.

## Results

All the problems were solved with GEARS using 10 different datasets for fitting and 10 additional datasets for cross-validation. For the RP and GO models an extra observable was considered in the case of the cross-validation. To illustrate the results obtained during the different steps, we will focus on the FHN problem. Detailed results for all the other case studies are given in Additional files 1 and 3. The GEARS software distribution includes all the scripts implementing these case studies.

First, the structural identifiability of these problems was analysed using STRIKE-GOLDD [82], concluding that all of them are identifiable a priori. This analysis also revealed that for the GO case study the initial conditions for the unobserved state *x*_2_ must also be known, otherwise two parameters are structurally unidentifiable.

**Fig. 4:**
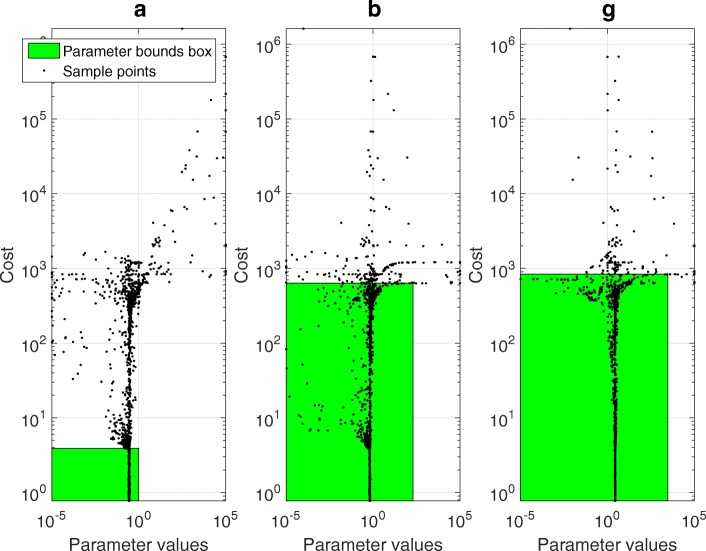
FHN case study: parameter samples. Parameter samples, showing the cost cut off values and the reduced bounds for each parameter

**Fig. 5:**
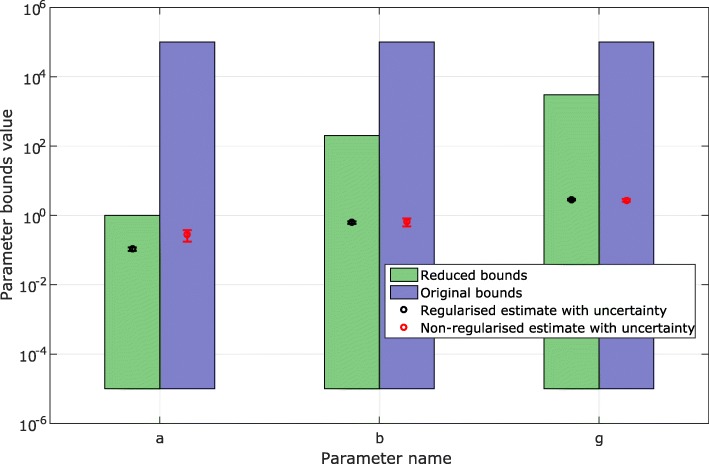
FHN case study: reduction of parameters bounds. Original and reduced parameter bounds, also showing the parameter confidence levels for the first fitting data set

**Fig. 6:**
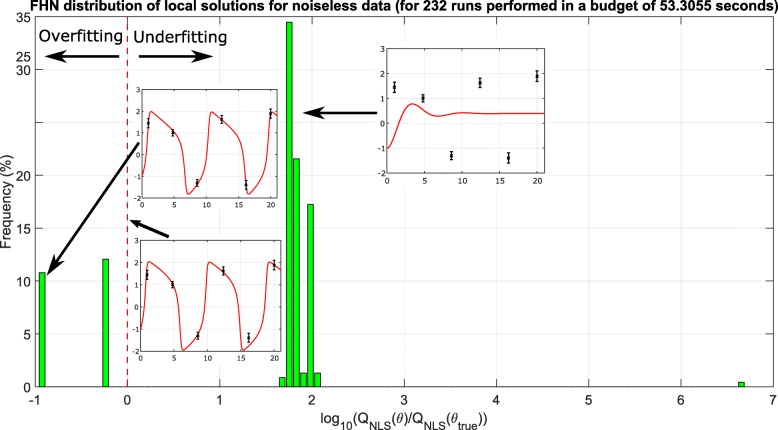
FHN case study: distribution of local solutions. Histogram of the local solutions found with a multi-start local solver; the arrows indicate examples of underfitting and overfitting

**Fig. 7:**
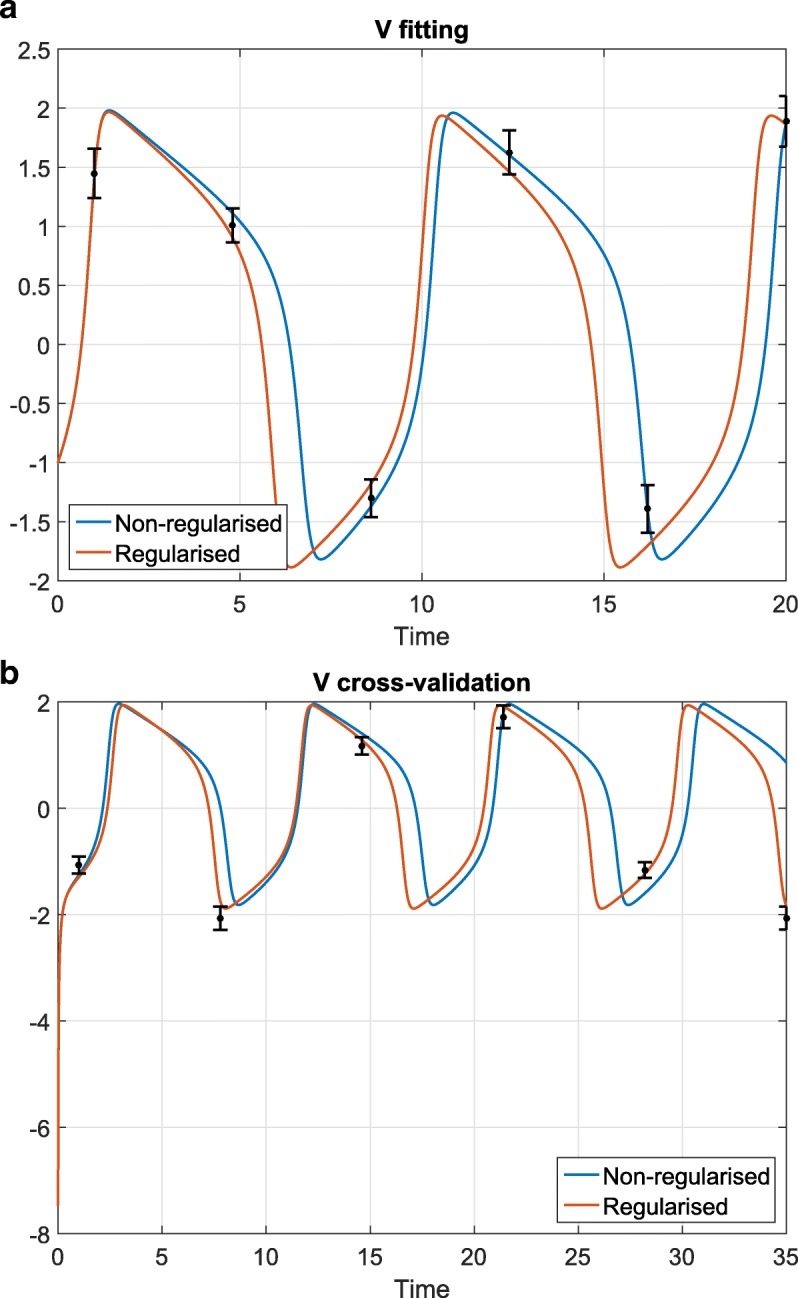
FHN problem: effect of regularisation on fitting and cross-validation. An example of how regularisation affects the calibration of the FHN model: reduction of the quality of the fitting (subplot (**a**)), but improvement on the quality of the cross-validation (subplot (**b**)); the corresponding numerical results are given in Table 4

**Fig. 8:**
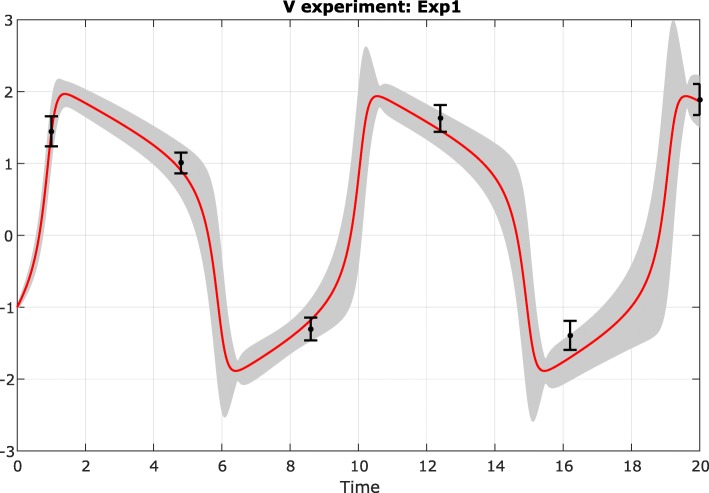
FHN problem fit with uncertainty: final regularised fit with uncertainty intervals coonsidering the first fitting data set

Next, the GEARS workflow proceeded performing an initial estimation. The samples obtained were analysed by applying a cut off to each parameter as seen in Fig. 4 for the FHN problem. The cost cut-off values were then used to significantly reduce the parameter bounds, as shown in Table 2 and Fig. 5. Next, GEARS performed the regularised estimation step (Table 3). We are successful able to avoid the multiple local solutions that are frequently found using local optimisers (see Fig. 6). As expected, this second estimation reduced the quality of the calibration to the fitting data, as shown in Table 4, Figs. 7a and 8. However, it increased the generalisability of the model, as can be seen in Fig. 7 and Table 4. Finally, GEARS confirmed a satisfactory practical identifiability for the resulting calibrated FHN, as indicated by the rather small confidence intervals for the estimated parameters.

**Table 2:** FHN case study: bounds reduction. A table showing the bounds reduction performed on the FHN model for the first fitting data set

Parameter	Original bounds	Reduced bounds
a	[10^−5^,10^5^]	[10^−5^,1]
b	[10^−5^,10^5^]	[10^−5^,1000]
g	[10^−5^,10^5^]	[10^−5^,100]

**Table 3:** FHN case study: summary of the regularised results. A summary of the final results of the procedure applied to the FHN model for the first fitting data set

Parameter names	Parameter value	Confidence (95%)	Coefficient of variation (%)	Bounds status
a	0.1069	0.0129	6.1732	Bounds not active
b	0.6230	0.0551	4.5130	Bounds not active
g	2.8490	0.1528	2.7370	Bounds not active

**Table 4:** FHN case study: summary of NRMSE results. A table of the NRMSE for the FHN case study. The fitting is to one particular experiment, while the cross-validation covers ten experiments for the fit to the first fitting data set

Experiments	Regularised	Non-regularised
Fitting	0.2647	0.1095
Cross-validation	1.4054	1.4487

More detailed results for FHN and all the other case studies can be found in Additional files 1 and 3. Regarding practical identifiability, using the VisId toolbox we found that both the FHN and RP problems are fully identifiable in practice indicating that our dataset contains enough information. In the case of the EO and GO models we found that there are a number of collinear parameters sets indicating that, at least to some degree, these parameters are compensating one another due to a lack of information in the data.

**Fig. 9:**
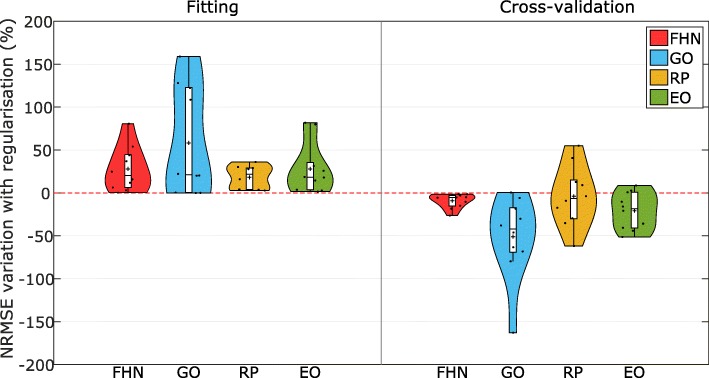
Effect of regularisation for all the case studies. Violin plots illustrating the effect of regularisation on the fitting and cross-validation errors (given as normalized root mean square error, NRMSE) for each model and over all the data sets considered. It is shown how regularisation increases the NRMSE for the fitting, but with the benefit of generally improving the predictive power, i.e. reducing the NRMSE in the cross-validations

Considering now all the case studies, it is important to assess the consistency of the effect of regularisation on their generalisability. In Fig. 9 we show how the regularised estimation always decreases the quality of calibration to the initial fitting data, as expected. However, the regularised estimations lead to better cross-validation results, i.e. these calibrated models have better predictive power because we have avoided overfitting. It should be noted, however, that there are a few cases where the procedure is unable to significantly improve the generalisability of the model. The explanation is that no real overfit was present in the initial calibrations.

In summary, using GEARS we have been able to successfully calibrate these challenging oscillators models, avoiding the typical pitfalls, including convergence to local optima and overfitting, in an automated manner.

## Conclusions

Parameter estimation in nonlinear dynamic models of biosystems is a very challenging problem. Models of biological oscillators are particularly difficult. In this study, we present a novel automated approach to overcome the most common pitfalls. Its workflow makes use of identifiability analysis and two sequential optimisation steps incorporating three key algorithms: (1) sampling strategies to systematically tighten the parameter bounds reducing the search space, (2) efficient global optimisation to avoid convergence to local solutions, (3) an advanced regularisation technique to fight overfitting.

We evaluate this novel approach considering four difficult case studies regarding the calibration of well known biological oscillators (Goodwin, FitzHugh–Nagumo, Repressilator and a metabolic oscillator). We show how our approach results in more efficient estimations which are able to escape convergence to local optima. Further, the use of regularisation allows us to avoid overfitting, resulting in more generalisable calibrated models (i.e. models with greater predictive power).

## Additional files

Additional file 1 Remarks on the eSS optimisation solver and detailed results for the Goodwin Oscillator problem and additional ENSO contour plots. This file describes our modifications to the eSS global optimisation solver. It also contains tables and figures showing the detailed results for the Goodwin Oscillator problem, and additional contour plots for the ENSO example. (PDF 1730 kb)

Additional file 2 Critical comparison of optimisation solvers. In GEARS, the optimisation problems are solved using the hybrid metaheuristic eSS. A comparison of the eSS global optimisation solver used in GEARS with other competitive local and global optimisation solvers. (PDF 880 kb)

Additional file 3 Detailed results for the Fitzhugh-Nagumo, Repressilator and Enzymatic Oscillator problems. This file contains tables and figures showing the detailed results for the Fitzhugh-Nagumo, Repressilator and Enzymatic Oscillator problems. (PDF 1680 kb)

## Abbreviations

EO: Enzymatic Oscillator model
eSS: Enhanced scatter search
FNH: FiztHugh-Nagumo model
GO: Goodwin Oscillator model
IVP: Initial value problem
NIST: National institute of standards
NLLS: Nonlinear least squares
RP: Repressilator model
