# Supplementary material for: Parameter estimation in models of biological oscillators: an automated regularised estimation approach
Source: BMC Bioinformatics. 2019 Feb 15;20:82. doi: 10.1186/s12859-019-2630-y (PMC6377730; doi:10.1186/s12859-019-2630-y)
Supplement: Supplementary file 1 — Remarks on the eSS optimisation solver and detailed results for the Goodwin Oscillator problem and additional ENSO contour plots. This file describes our modifications to the eSS global optimisation solver. It also contains tables and figures showing the detailed results for the Goodwin Oscillator problem, and additional contour plots for the ENSO example. (PDF 1730 kb) [file 12859_2019_2630_MOESM1_ESM.pdf]

Additional file 1 for “Parameter estimation in models of biological oscillators: an automated regularised estimation approach.”

Jake Alan Pitt[1,2] and Julio R. Banga[1*]

1 (Bio)Process Engineering Group, Spanish National Research Council, IIM-CSIC, C/Eduardo Cabello 6, 36208, Vigo, Spain
2 RWTH-Aachen University Hospital, Joint Research Centre for Computational Biomedicine (JRC-COMBINE), 52074, Aachen, Germany

[*]Correspondence should be directed to Prof J. R. Banga at julio@iim.csic.es

S1.1 Analysis of logarithmic scaling in eSS

The original scatter search method used for parameter estimation in dynamic models already incorporated logarithmic sampling of the search space (Rodriguez-Fernandez et al., 2006). In that algorithm, a logarithm scaling was applied to the generation of the initial reference set of the parameter values, allowing for an initial diversification of the exploration of the parameter space. However, logarithm scaling was not used in other parts of the search. In recent years, several studies (Raue et al., 2013; Fröhlich et al., 2017; Kreutz, 2016; Villaverde et al., 2018) have proposed to perform the whole parameter estimation in logarithm space.

In this study we compared three different sets-ups: eSS with the entire search in log scale, eSS with all the search except for the local solver in log scale and the default formulation with only the initial reference set being in log scale. We tested each of the scaling set-ups for all four case studies, for both noisy and noiseless data (the first fitting data set is used as the noiseless data). To test the methods we run eSS 30 times for each case running eSS with the stopping criteria of reach the global optima’s (known) cost value or a hard cut off of 3 hours. We found that in every case running the local search in linear scale and rest of eSS in log scale was the most efficient method.

S1.1.1 Analysis with noisy data

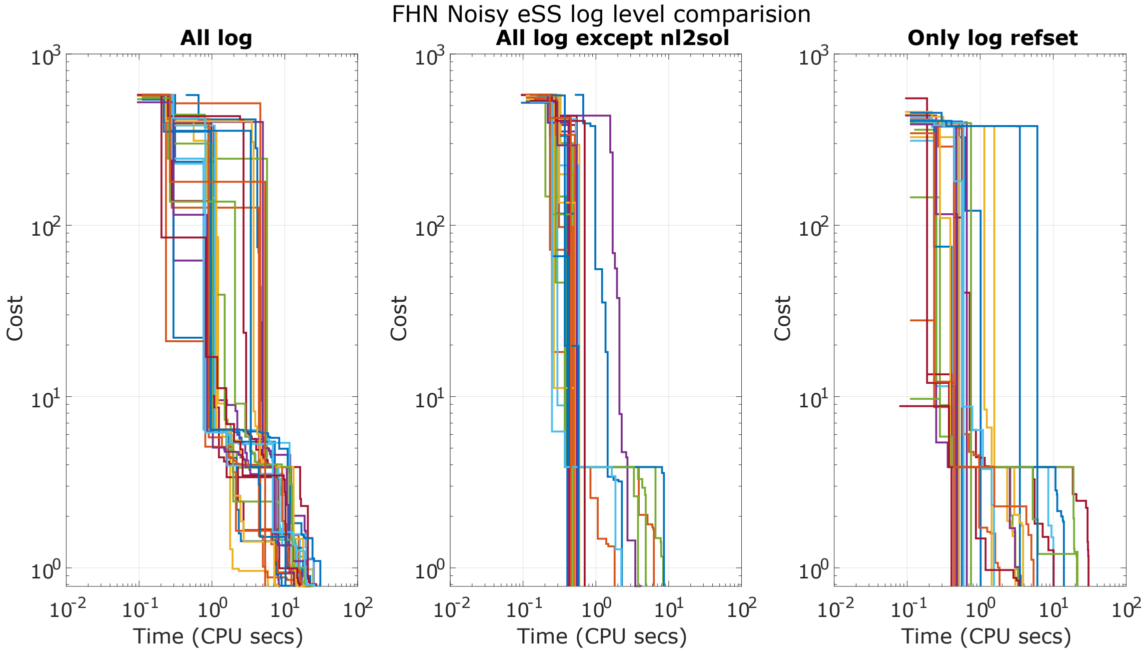

Figure S1.1: FHN case study: A comparison of how the using eSS with different sections of the solver in log scale effects eSS' efficiency when fitting to noisy data.

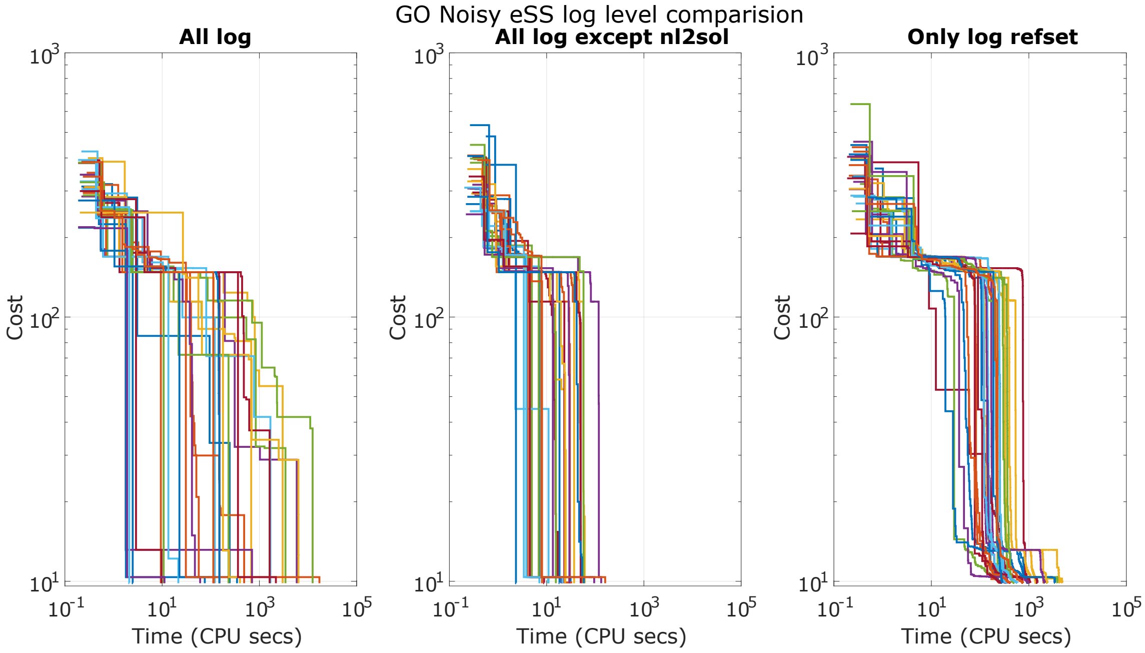

Figure S1.2: GO case study: A comparison of how the using eSS with different sections of the solver in log scale effects eSS' efficiency when fitting to noisy data

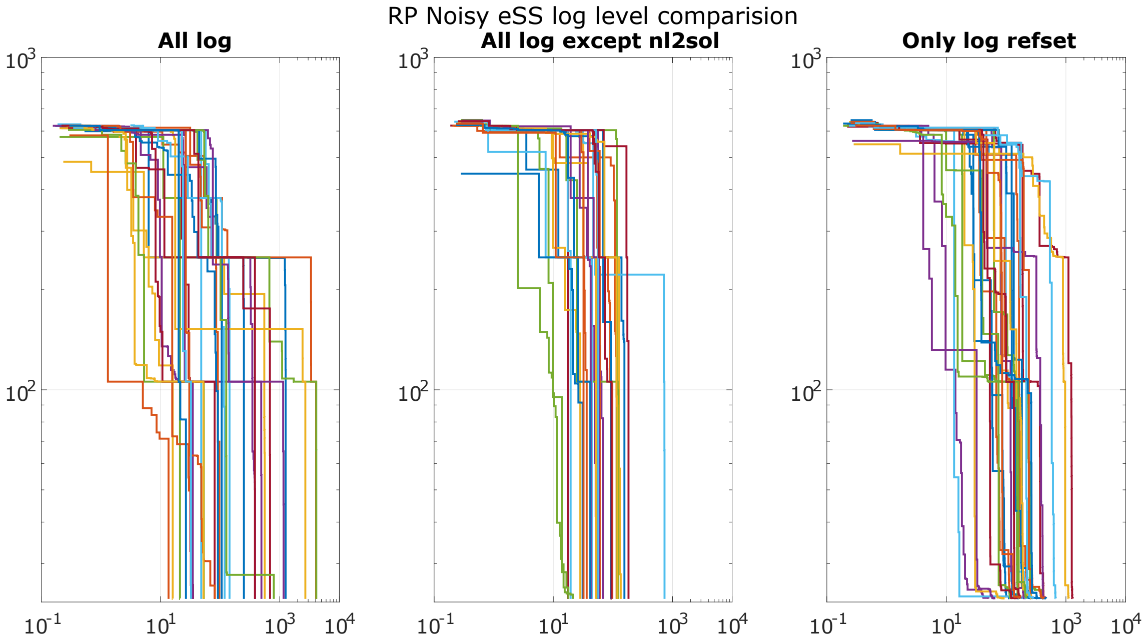

Figure S1.3: RP case study: A comparison of how the using eSS with different sections of the solver in log scale effects eSS' efficiency when fitting to noisy data.

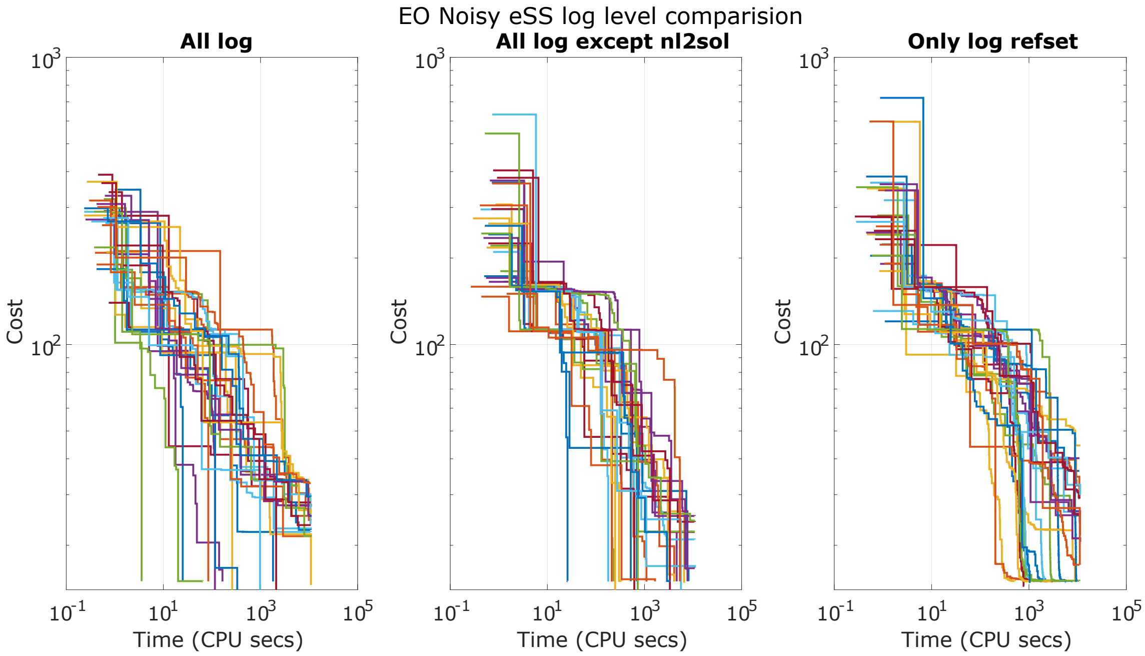

Figure S1.4: EO case study: A comparison of how the using eSS with different sections of the solver in log scale effects eSS' efficiency when fitting to noisy data.

S1.1.2 Analysis with noiseless data

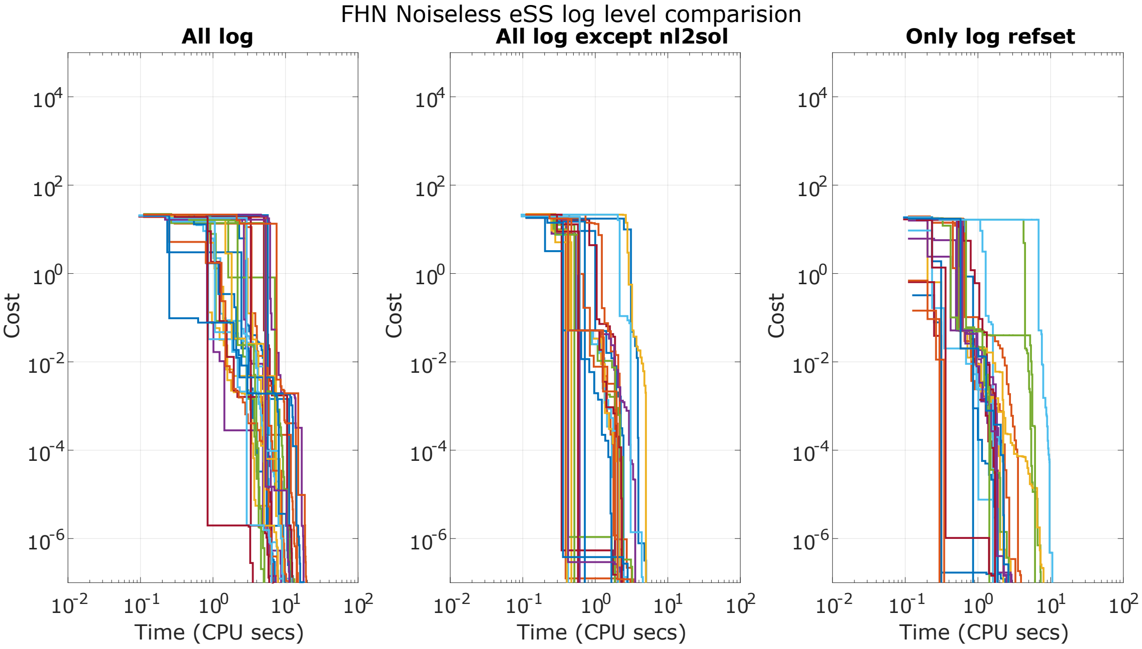

Figure S1.5: FHN case study: A comparison of how the using eSS with different sections of the solver in log scale effects eSS' efficiency when fitting to noiseless data.

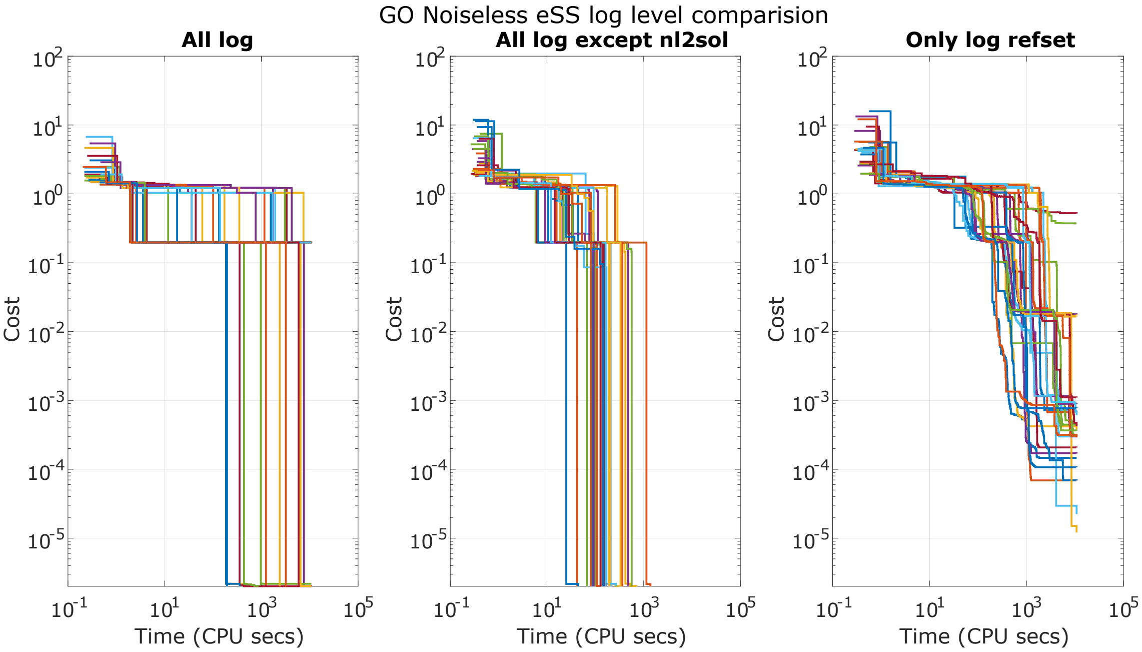

Figure S1.6: GO case study: A comparison of how the using eSS with different sections of the solver in log scale effects eSS' efficiency when fitting to noiseless data.

In summary, we found that, at least for the problems considered here, the most efficient search scaling in eSS to perform the diversification search in logarithm scale but not the local search. We found that this was the most efficient method when fitting to both noisy

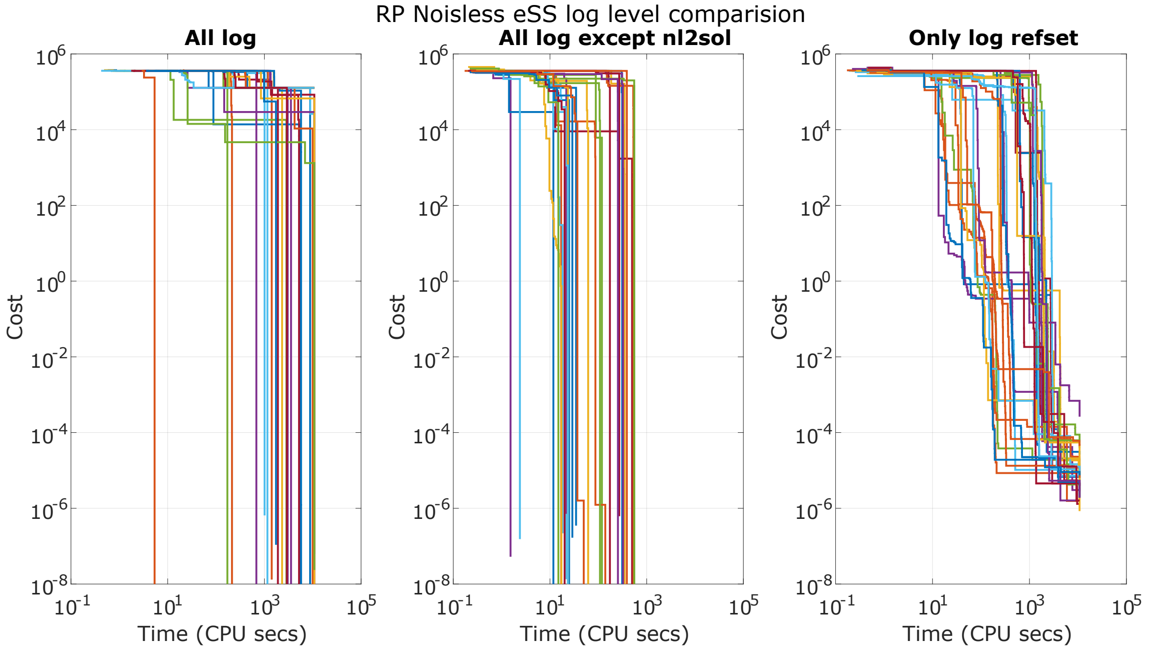

Figure S1.7: RP case study: A comparison of how the using eSS with different sections of the solver in log scale effects eSS' efficiency when fitting to noiseless data.

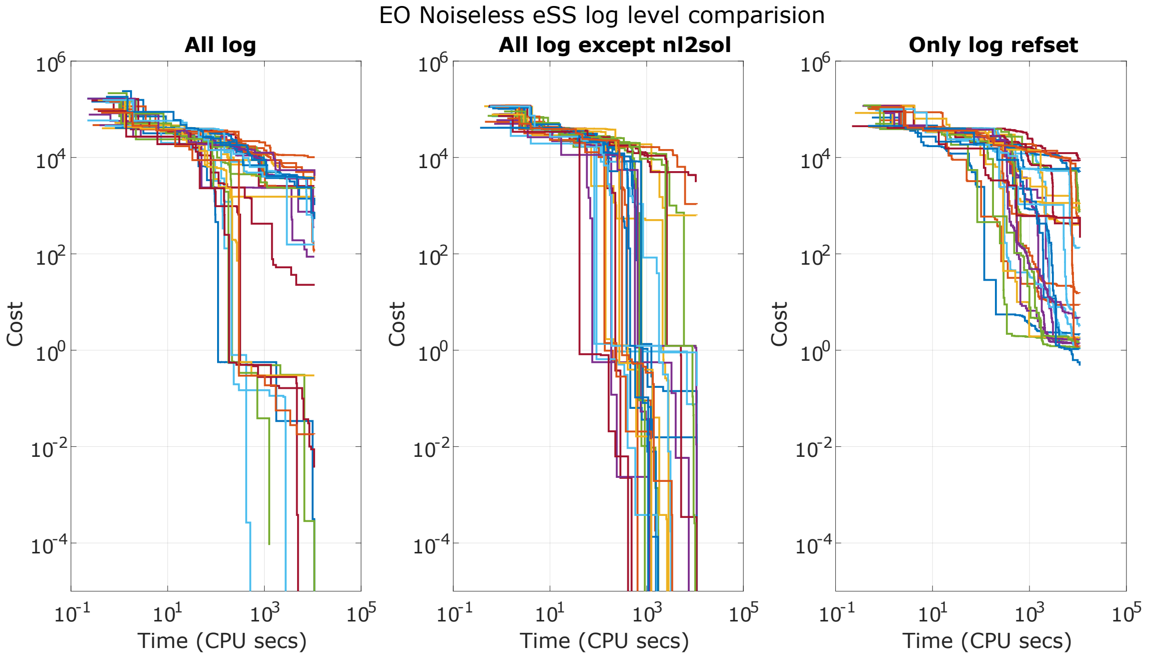

Figure S1.8: EO case study: A comparison of how the using eSS with different sections of the solver in log scale effects eSS' efficiency when fitting to noiseless data.

and noiseless models, for all the oscillators case studies considered.

S1.2 Computational reproducibility of eSS

Reproducibility is a major issue in computational research. The fact that `eSS` is a stochastic solver needs to be taken into account. Stochastic optimisers use some type of random number generator in their sampling of search space. Modern pseudo-random number generators use a seed which, if fixed, determines the sequence of pseudo-random values. Therefore, starting eSS runs with different seeds will result in different optimisation path followed, although most runs will converge to essentially the same final solution (depending on the stopping criteria chosen).

To illustrate this, in Figure S1.9 we plot the contours of the ENSO problem (considering the projection for 2 parameters) and then use eSS to solve the problem starting from the same initial guess multiple times, changing the seed randomly in each run. This results in different optimisation paths which ultimately arrive to essentially the same final solution.

In theory, fixing the seed should result in the same optimisation path. However, in `GEARS` we use all the information from the path taken in the form of parameter-cost distributions, which can lead to different solutions when the procedure is re-run. That is, even when starting from the same seed (so that the same pseudo-random sequence will be generated), slight differences caused by the stopping criteria can affect the result. This is because some types of stopping criteria are not checked during the local search phases. For example, if we have set `eSS` to stop after 2000 function evaluations in one run, it might however stop after 2003 evaluations in another. As all the information for every parameter point is used, these 3 extra points would cause differences that would be passed downstream and cause a lack of strict computational reproducibility.

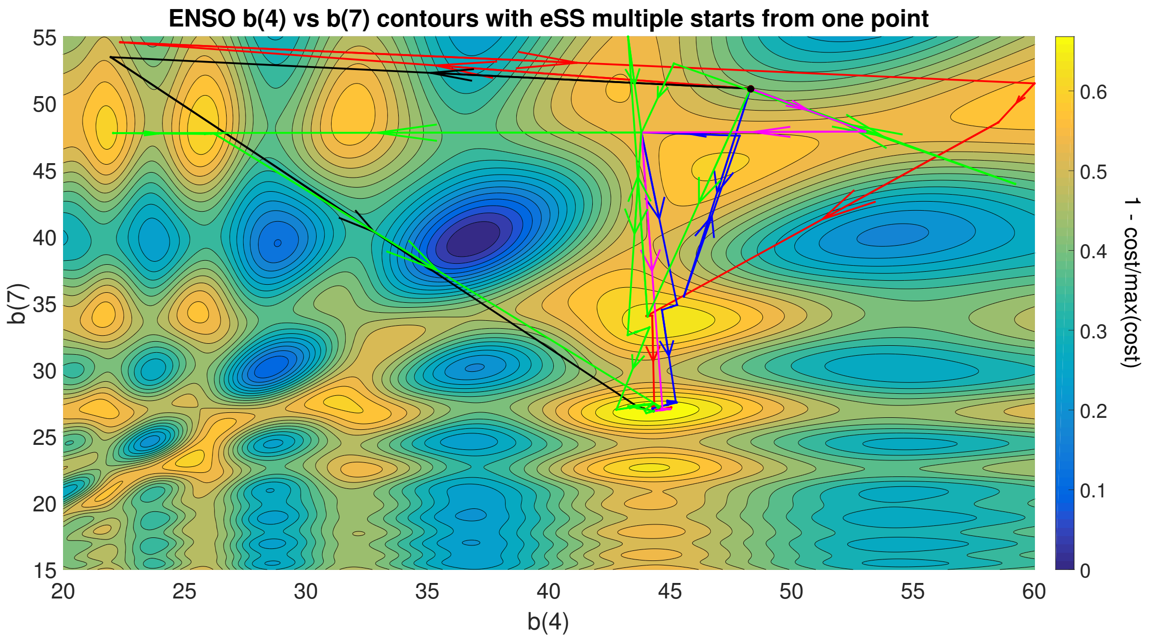

Figure S1.9: ENSO case study: Contour plots with multiple starts of the eSS solver from the same initial point showing that the stochastic nature of the solver results in different paths being taken.

S1.3 Multimodality in the ENSO case study

In the case of the ENSO model by plotting contour plots we can clearly see the multimodality of the model, even when only considering two of the parameters (for visualisation purposes). In Figure S1.10 we can see the existence of many local optima in the search space. This many peak situations is essentially a worst case scenario in parameter estimation, showing extreme mulitmodality, making the parameter estimation problem extremely challenging to solve.

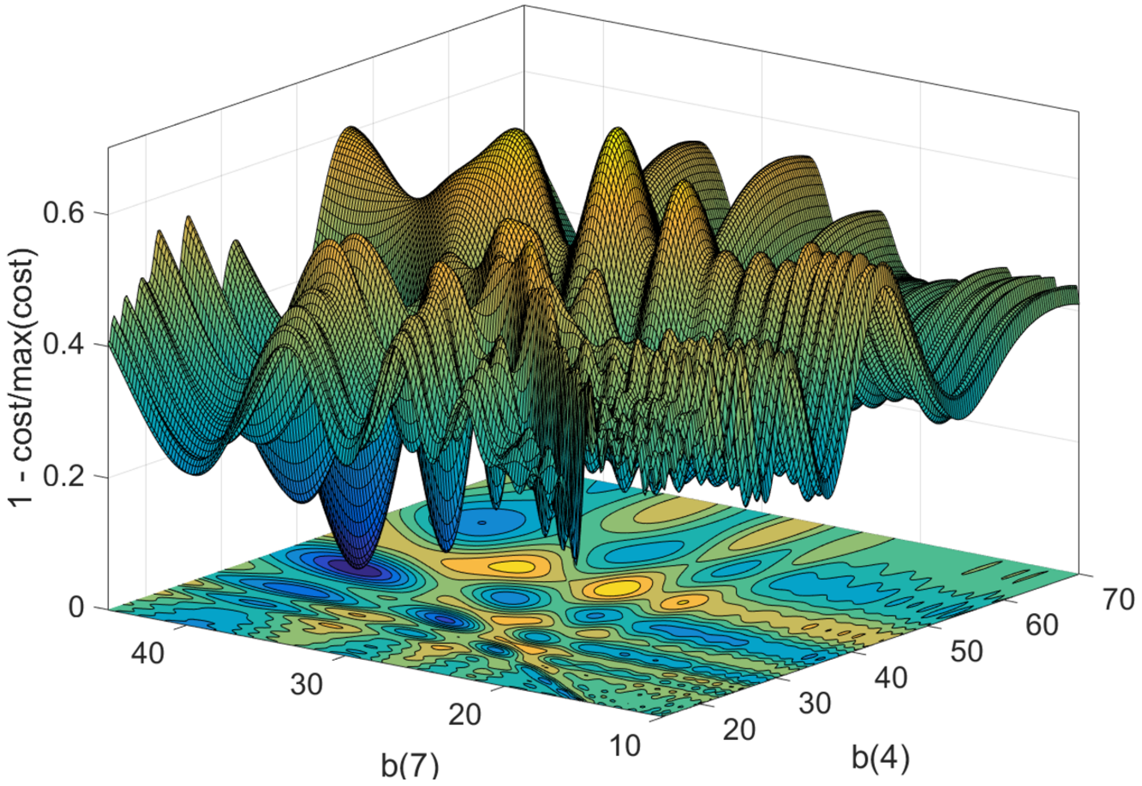

Figure S1.10: ENSO case study: Contour plots of the b(4) and b(7) parameters plotted in three dimensions.

S1.4 GO problem: detailed results

The results for running the analysis on the GO case study can be found here.

Table S1.1: GO case study: A summary of the results for the regularised fit to the first fitting data set.

Parameter	Value	Confidence (95%)	Coeff of variation (%)	Bounds status
k_1	2.4731	± 0.034618	0.714172	Bounds not active
k_2	0.0917	± 0.011088	6.17051	Bounds not active
k_3	0.9477	± 0.13256	7.13619	Bounds not active
k_4	0.1428	± 0.019794	7.07017	Bounds not active
k_5	1.2455	± 0.24837	10.1739	Bounds not active
k_6	0.0861	± 0.015879	9.40842	Bounds not active
K_i	0.9638	± 0.18518	9.80278	Bounds not active
n	11.7721	± 2.1469	9.30473	Bounds not active

Table S1.2: GO case study: NRMSE values for the fitting for each fitting data set, with and without regularisation.

	Regularised	Non-regularised
Fitting set 1	5.96798	4.77509
Fitting set 2	37.3928	4.28762
Fitting set 3	23.0941	5.07253
Fitting set 4	9.13458	7.46763
Fitting set 5	30.7735	30.7977
Fitting set 6	21.6336	5.21221
Fitting set 7	30.5141	30.3792
Fitting set 8	12.699	10.3537
Fitting set 9	29.6946	29.6743
Fitting set10	29.0661	8.60332

Table S1.3: GO case study: NRMSE values for the cross-validation for each regularised fit to the fitting data. Here, CV denotes cross-validation data set and F denotes fitting data set.

	F 1	F 2	F 3	F 4	F 5	F 6	F 7	F 8	F 9	F 10
All CV	49.1531	45.6818	92.87566	82.02807	124.931	43.5472	45.5031	64.29963	132.8635	237.2202
CV 1	90.2766	44.5872	91.60485	98.67496	160.9028	42.7661	44.6539	54.58628	139.1305	81.22797
CV 2	17.4337	55.6168	108.2136	102.4116	137.4336	51.4413	55.3724	75.44783	181.2703	154.689
CV 3	59.2786	41.4897	132.7566	81.33378	190.7445	41.0993	41.7198	100.1812	151.4091	55.42433
CV 4	49.6364	41.3489	56.58716	57.79468	77.52544	37.2885	41.3348	65.96083	50.11638	364.066
CV 5	15.5789	48.1839	103.2592	87.23705	107.1258	48.9272	47.8465	37.84106	135.5939	46.12598
CV 6	33.8966	45.9419	66.3954	64.4661	96.05532	46.6294	45.4043	82.39392	75.67943	85.2841
CV 7	45.3068	40.5613	52.82711	76.48417	70.68241	36.401	40.1927	33.8625	44.96142	396.7472
CV 8	77.0161	45.5434	81.13562	90.45528	127.3902	43.8462	45.1954	55.16384	139.7722	258.7255
CV 9	14.8661	42.0491	111.9855	83.81761	126.1179	38.2899	42.1025	52.71671	186.9935	62.97651
CV10	11.4834	49.3559	91.35434	65.43839	105.2309	46.1197	49.1384	55.64005	135.0444	398.5941

Table S1.4: GO case study: NRMSE values for the cross-validation for each non-regularised fit to the fitting data. Here, CV denotes cross-validation data set and F denotes fitting data set.

	F 1	F 2	F 3	F 4	F 5	F 6	F 7	F 8	F 9	F 10
All CV	52.0968	446.13711	188.8501	157.9609	183.2273	101.2102	45.2463	77.25336	179.9307	379.0375
CV 1	87.4309	70.334685	230.7914	97.35506	234.9919	105.9857	44.8763	62.66137	191.5425	49.92867
CV 2	19.8086	21.002245	206.6961	22.64062	209.0168	108.3317	54.5939	61.06428	245.0328	44.90806
CV 3	85.5105	81.308119	230.8422	86.60813	277.54	104.0653	41.9434	53.90163	207.3404	18.88773
CV 4	45.554	695.14654	120.9753	329.22	85.05097	98.38078	41.7637	138.5818	56.63287	692.9271
CV 5	19.4379	15.11765	202.2991	25.42743	161.2799	109.9085	47.6127	30.18765	182.0685	17.9874
CV 6	58.6734	72.38579	145.8185	17.15099	126.1488	106.6444	44.6755	81.10734	95.45583	39.55832
CV 7	60.3253	1218.8307	103.5804	347.3703	83.11907	90.66787	40.1697	115.7737	48.90935	972.3396
CV 8	39.6467	58.424156	196.2636	36.97415	195.5502	98.56097	44.6943	82.0453	193.4304	50.32223
CV 9	16.1404	15.545342	205.9824	16.43928	197.5051	64.82991	41.7203	34.1444	254.2339	31.86496
CV 10	20.6174	21.998584	197.1481	19.87449	162.6939	115.6165	48.5776	33.53653	184.7171	28.27294

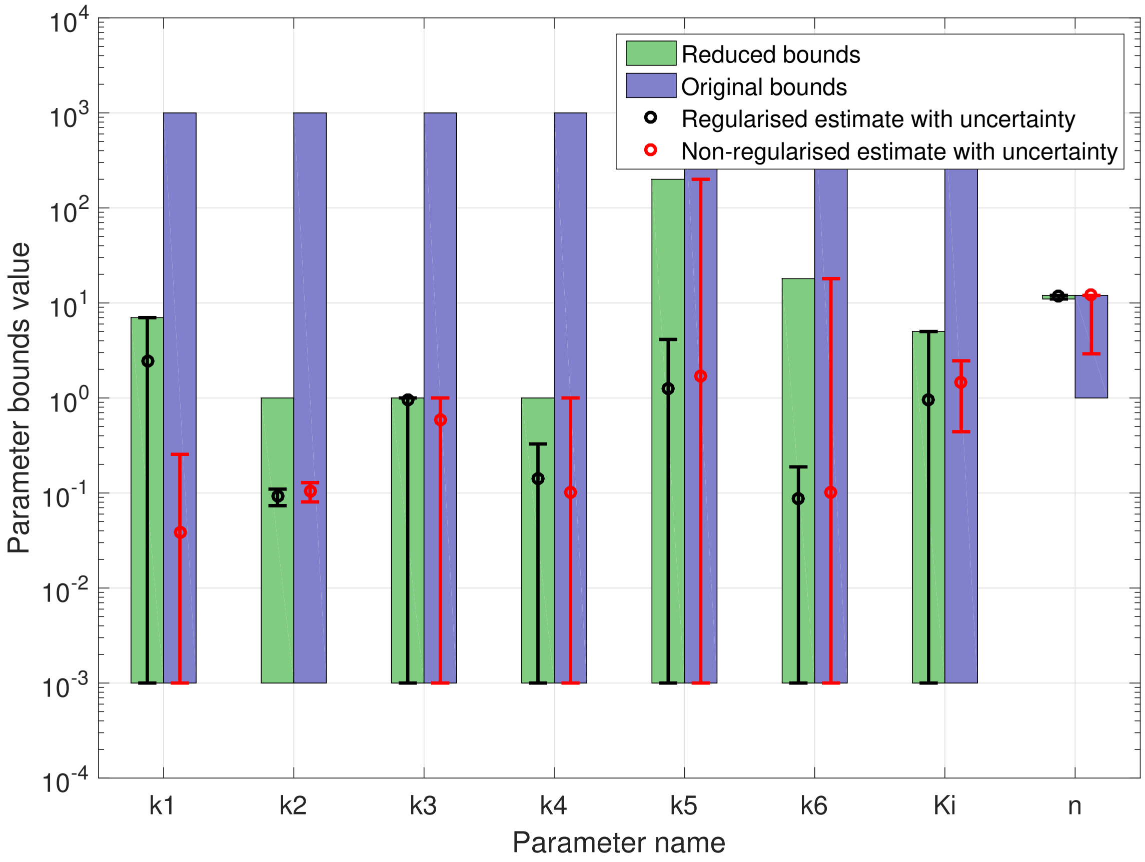

Figure S1.11: GO case study: reduction in the parameter bounds with the estimated values and their 95% confidence intervals for the first fitting data set.

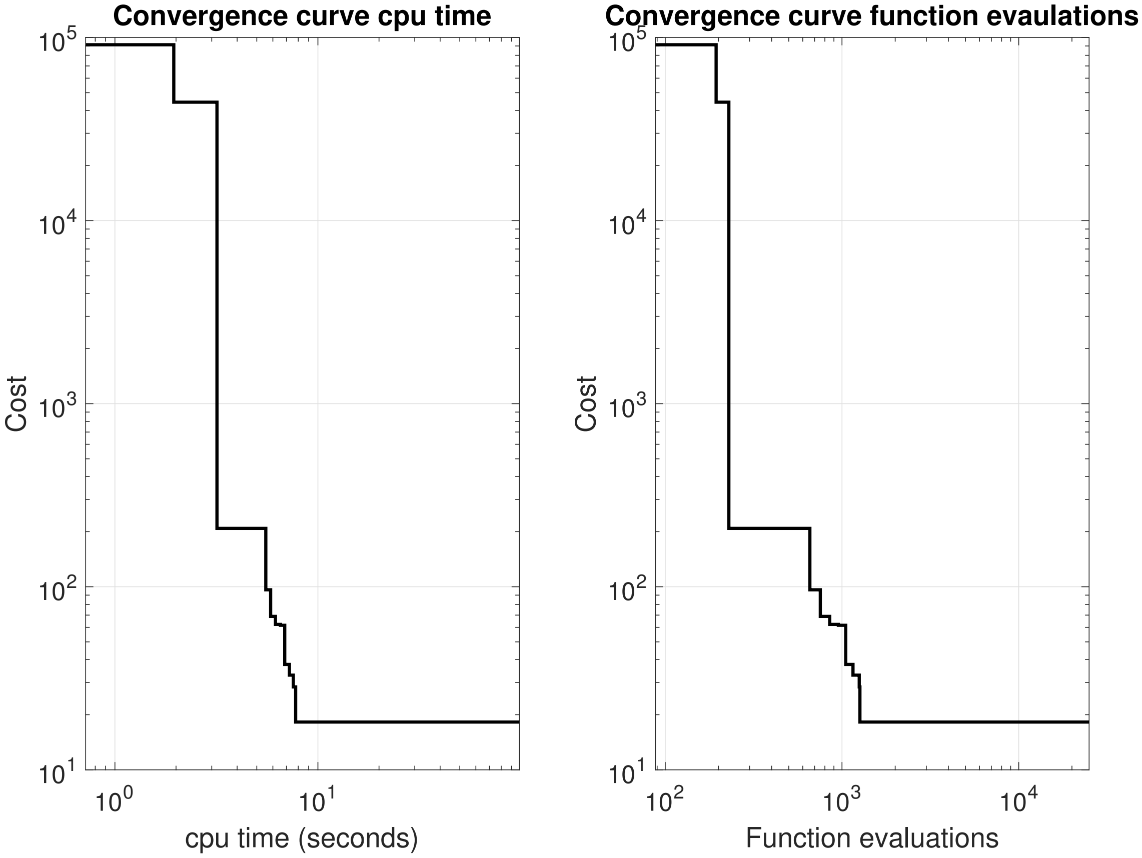

Figure S1.12: GO case study: convergence curve of the final regularised estimation for the first fitting data set.

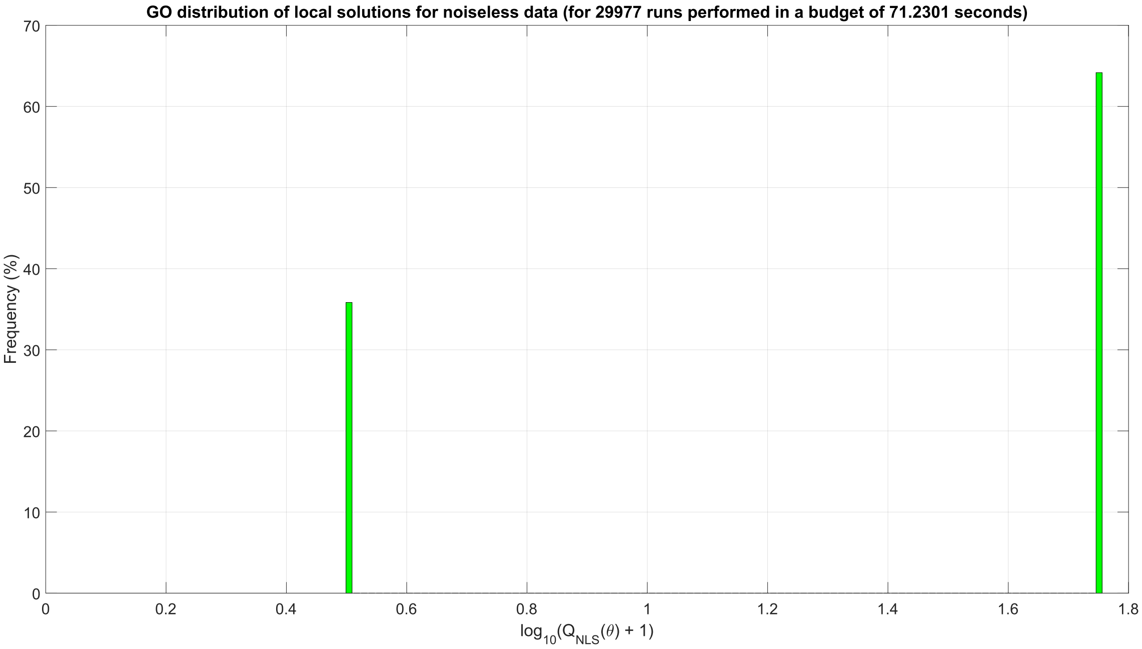

Figure S1.13: GO case study: distribution of local solutions found using the nl2sol local solver fitting to noiseless data.

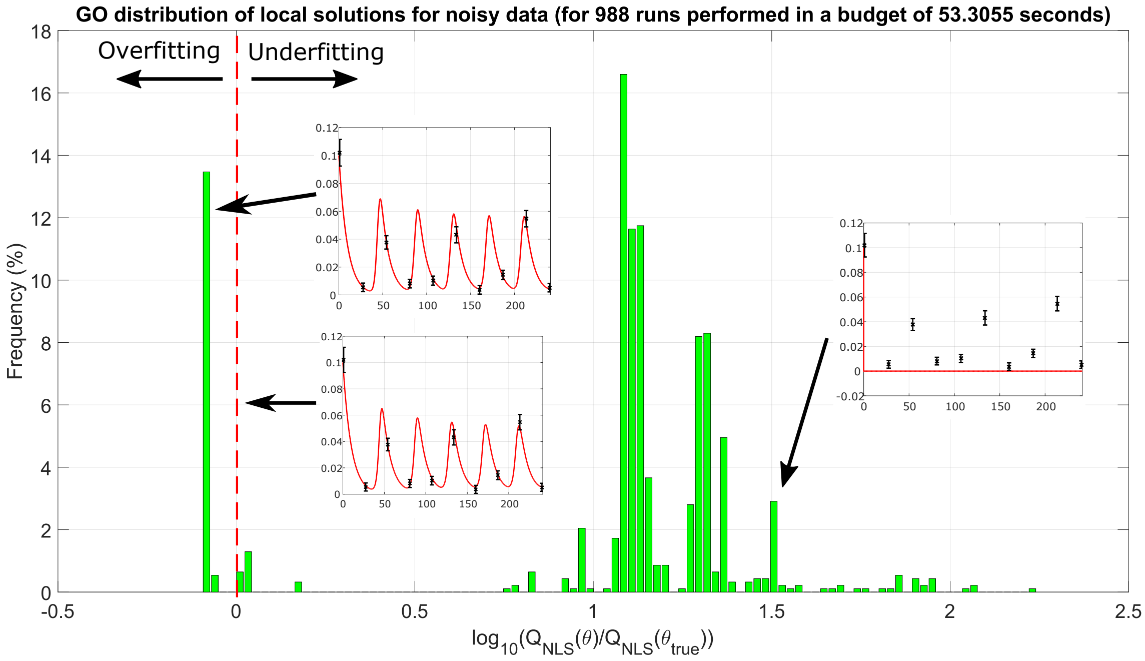

Figure S1.14: GO case study: distribution of local solutions found using the nl2sol local solver, with examples of local solutions and overfitting for the first fitting data set.

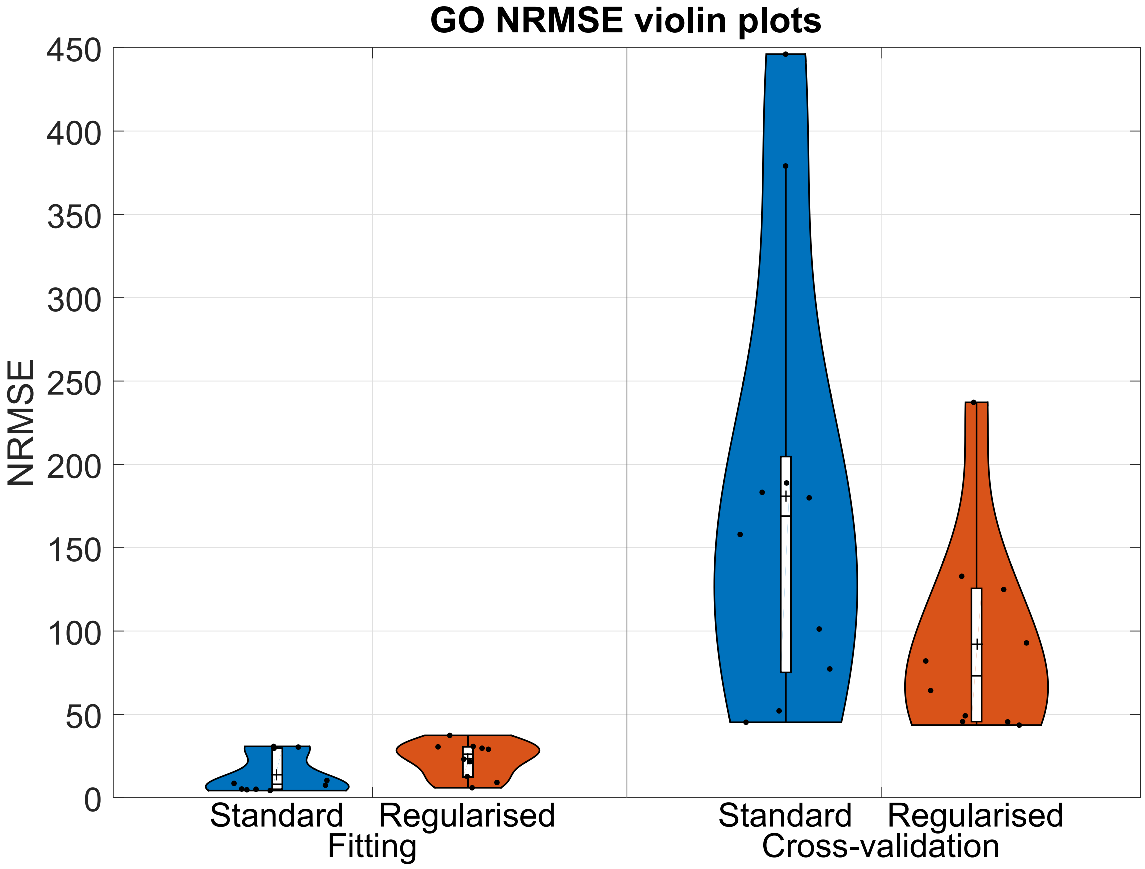

Figure S1.15: GO case study: violin plotss showing the distribution of the NRMSE for the fit and cross-validation for all the data sets considered, both with and without regularisation.

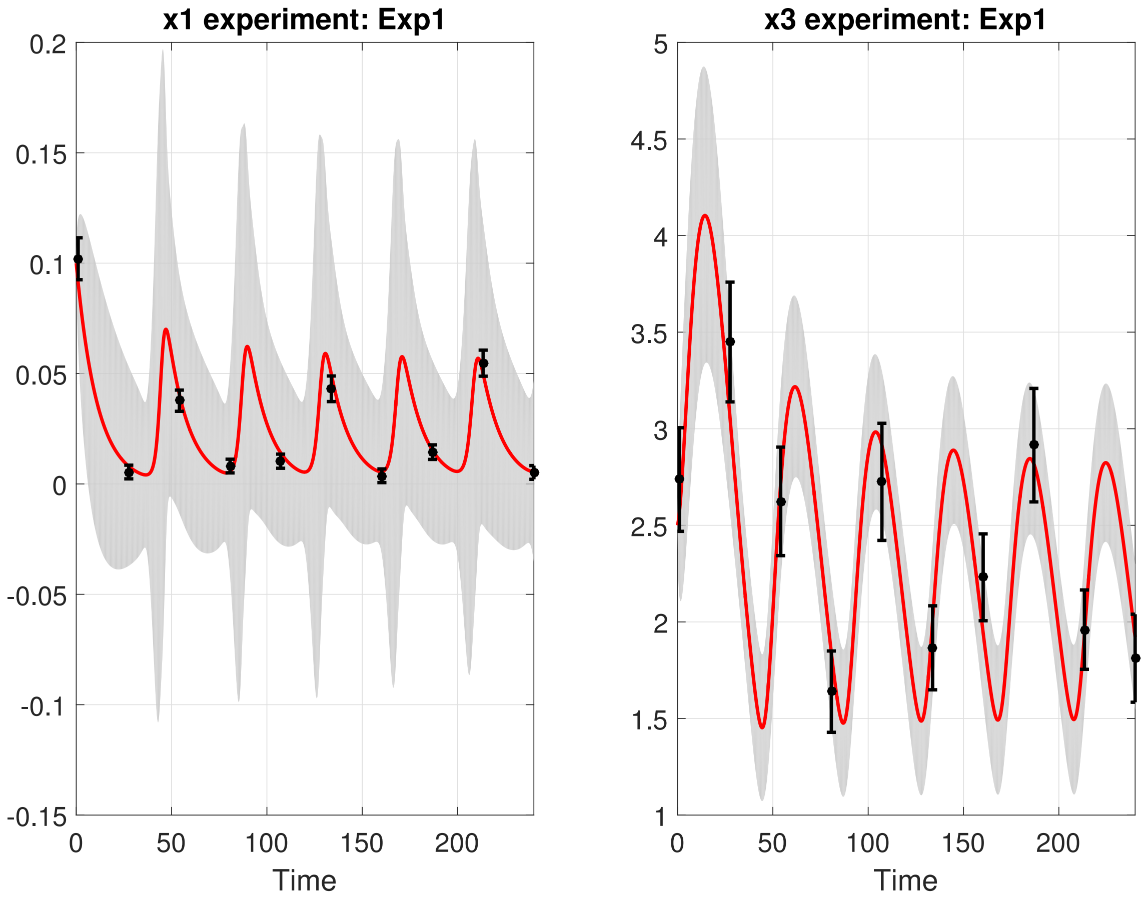

Figure S1.16: GO case study: final regularised fit with uncertainty intervals for the first fitting data set.

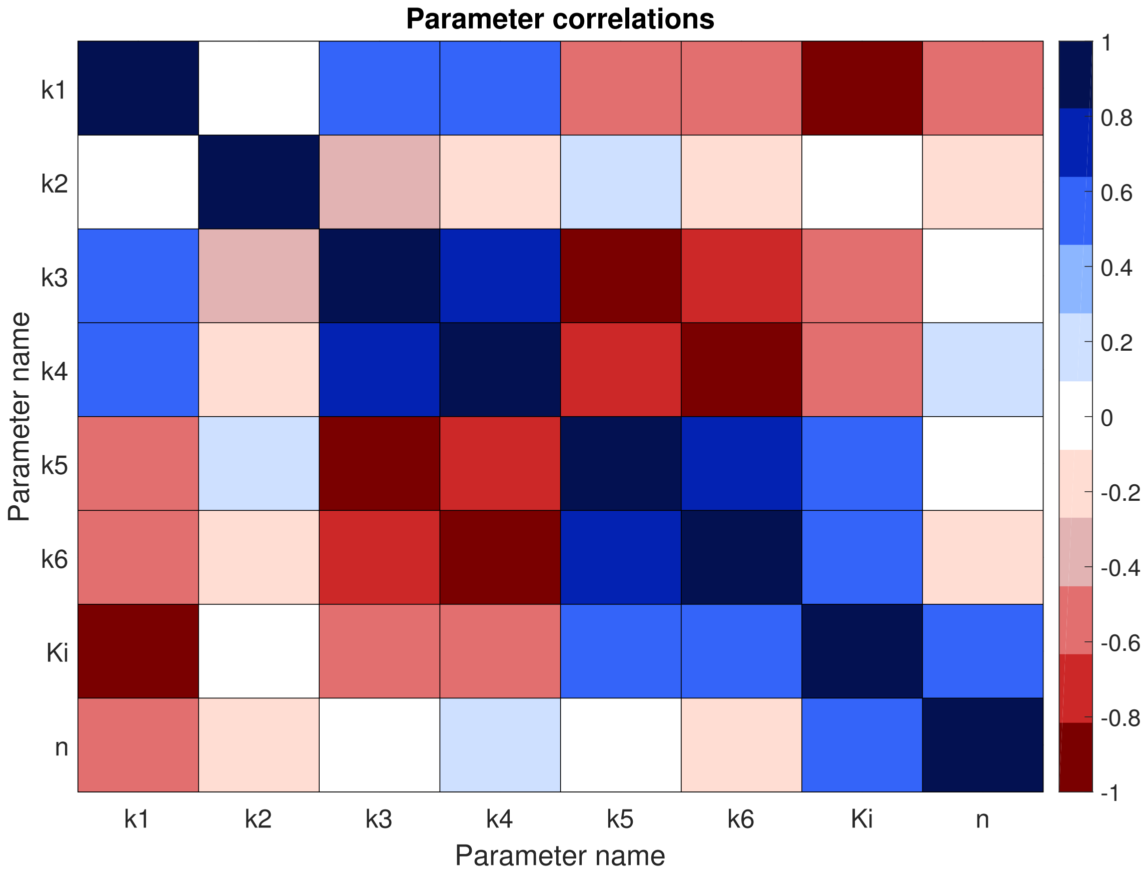

Figure S1.17: GO case study: parameter correlation matrix for the final estimated regularised solution for the first fitting data set.

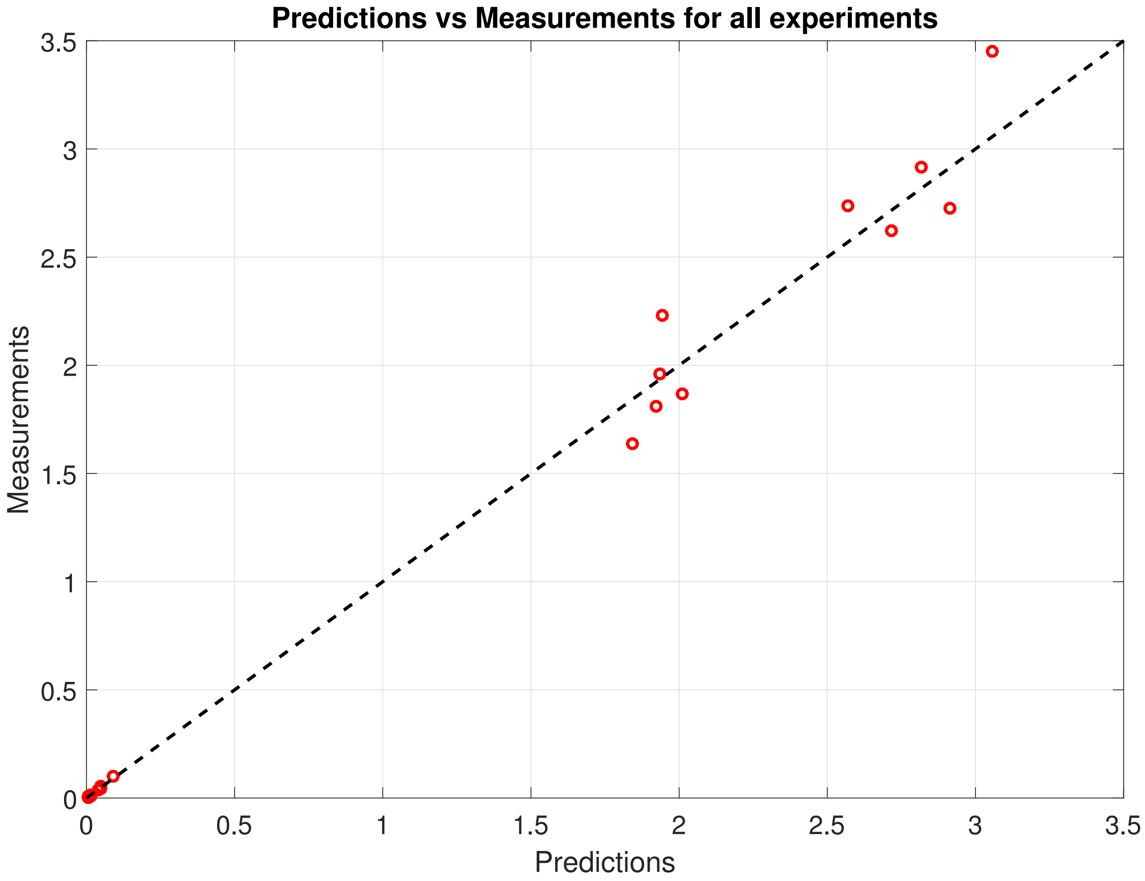

Figure S1.18: GO case study: predicted versus measured values for the first fitting data set.

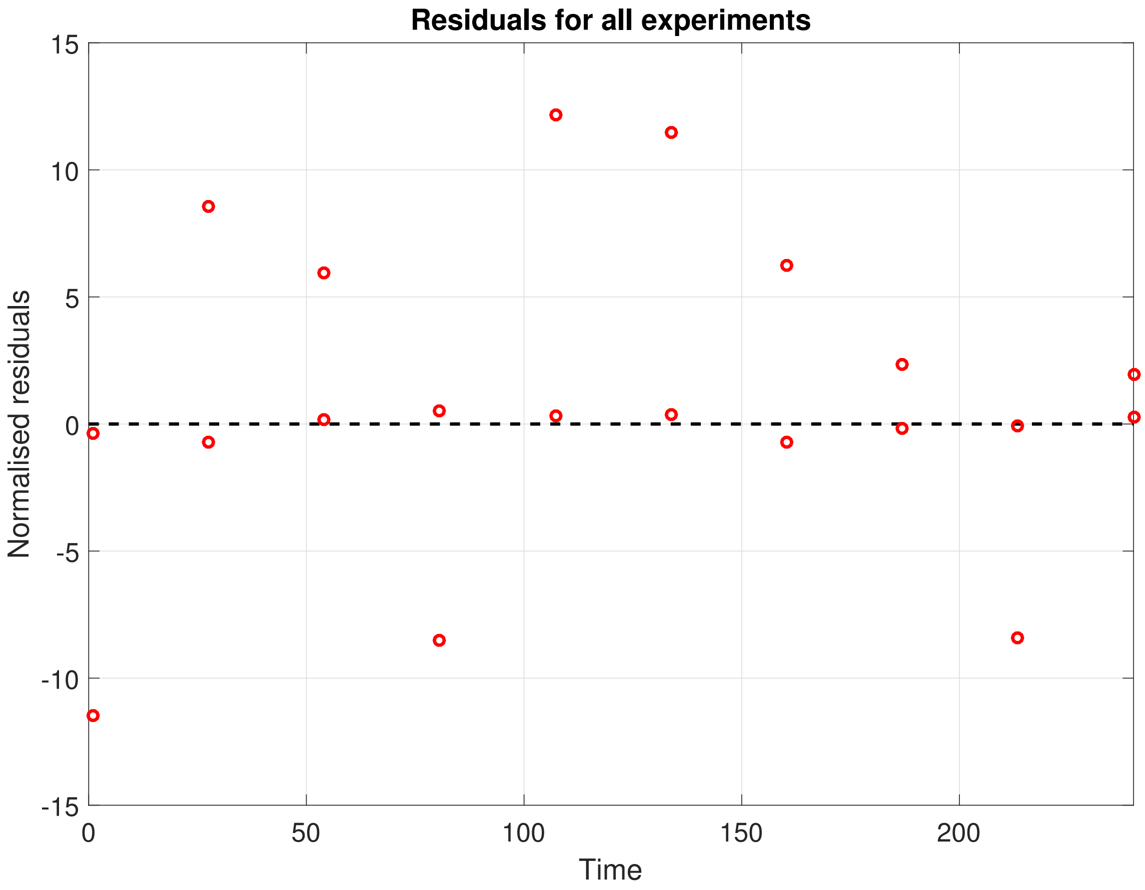

Figure S1.19: GO case study: normalised residuals for the regularised fit for the first fitting data set.

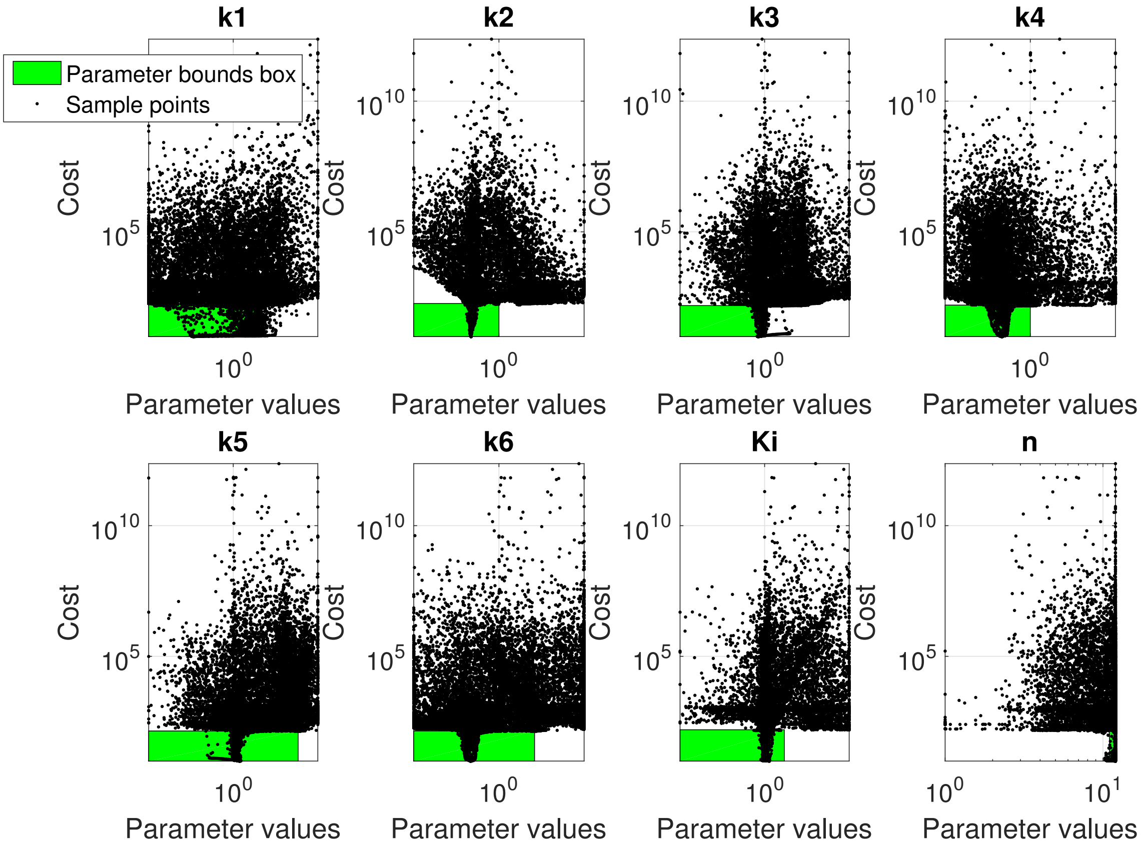

Figure S1.20: GO case study: sampling from the initial estimation with the new parameter bound box, where the height of said box is the cost cut off for the first fitting data set.

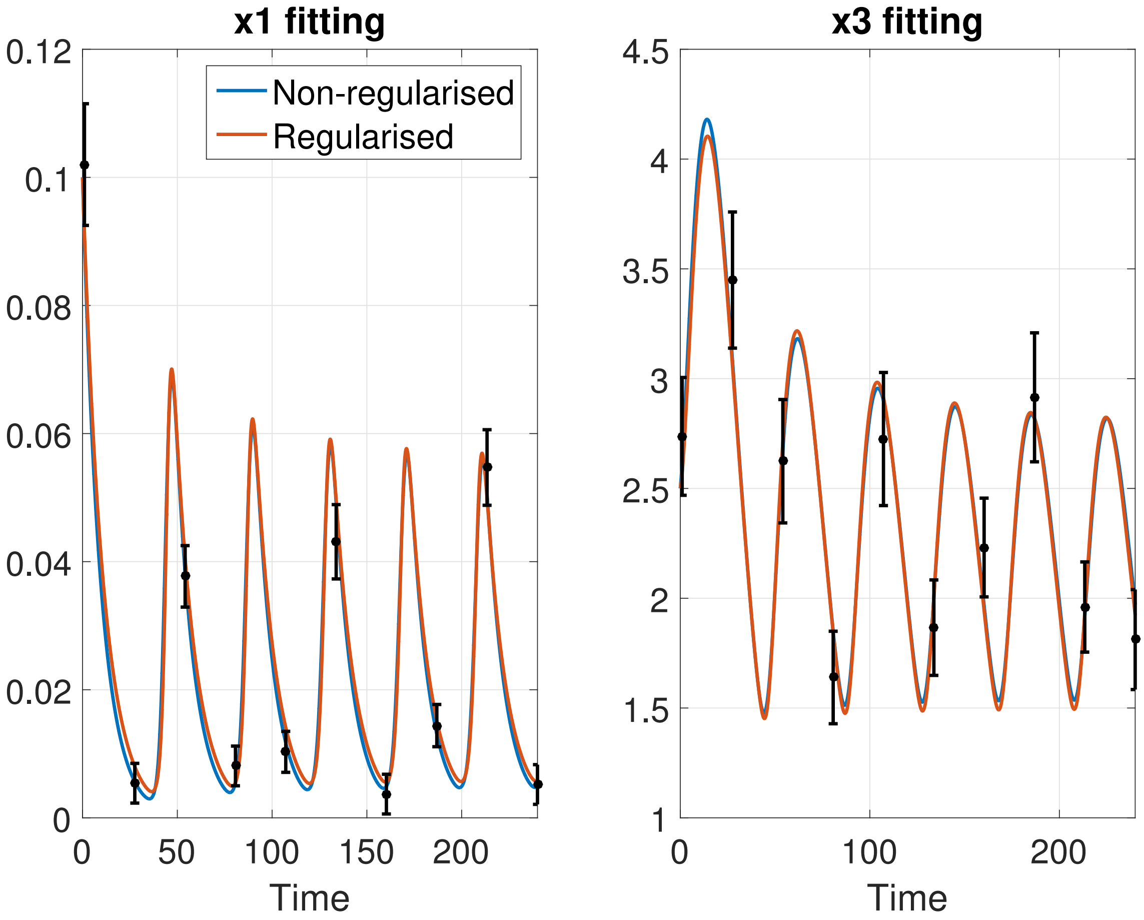

Figure S1.21: GO case study: comparison of the fits with and without regularisation for the first fitting data set.

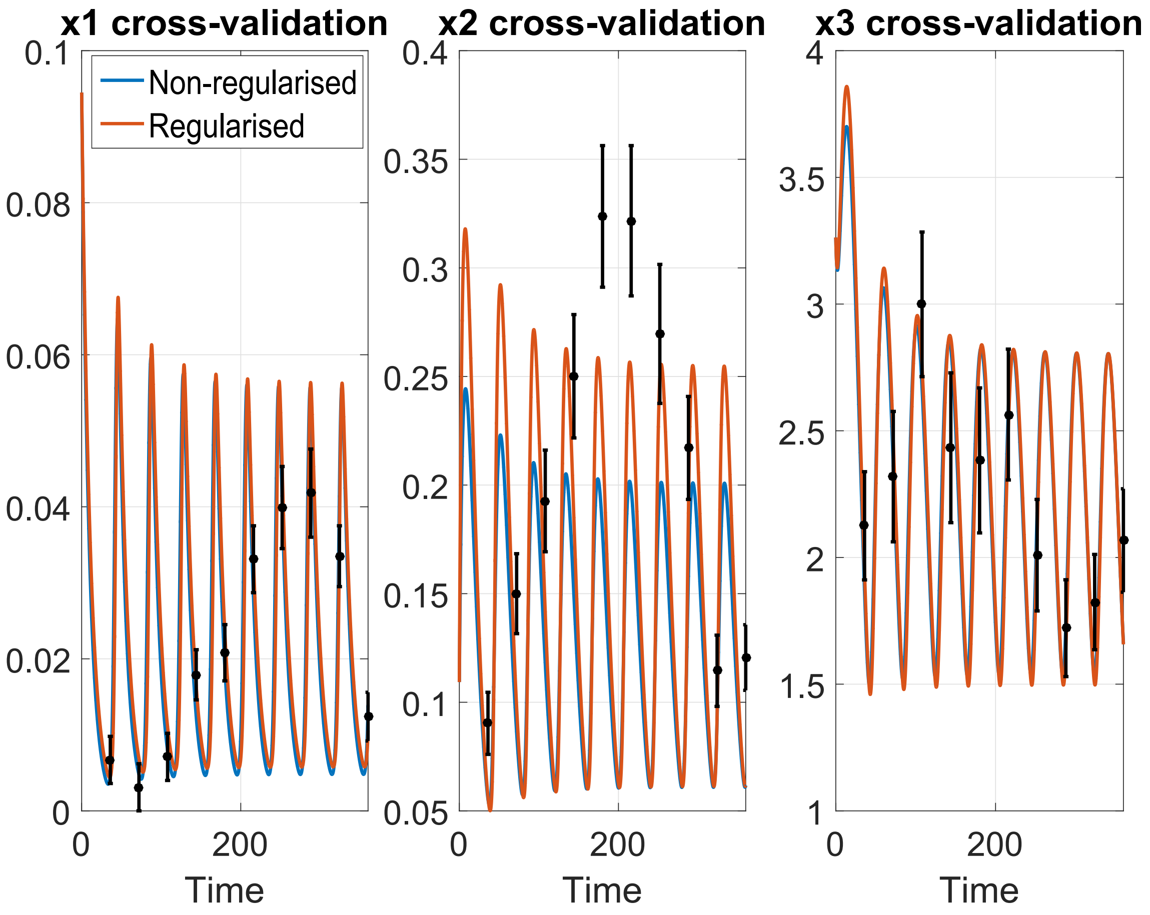

Figure S1.22: GO case study: comparison of the cross-validation with and without regularisation for the fit to the first fitting data set.

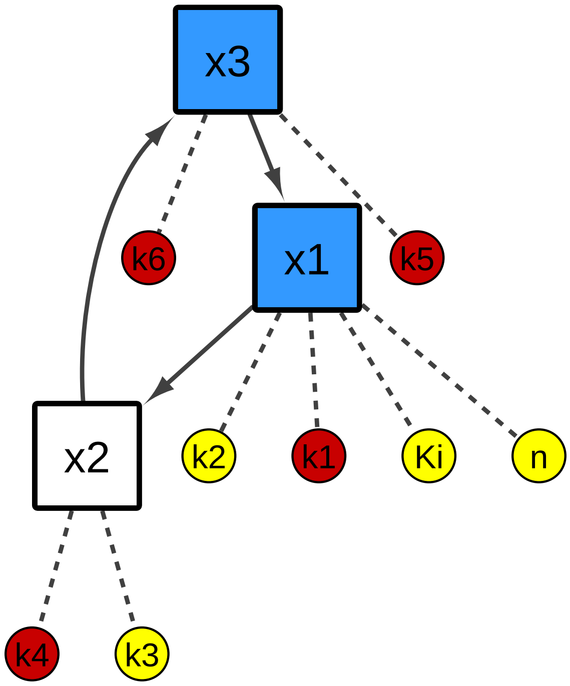

Figure S1.23: GO case study: results of the VisId analysis performed at the regularised solution for the first fitting data set.

References

Fröhlich, F., Theis, F., Rädler, J., and Hasenauer, J. (2017). Parameter estimation for dynamical systems with discrete events and logical operations. *Bioinformatics*, 33(7):1049–1056. cited By 0.

Kreutz, C. (2016). New concepts for evaluating the performance of computational methods. *IFAC-PapersOnLine*, 49(26):63–70.

Raue, A., Schilling, M., Bachmann, J., Matteson, A., Schelke, M., Kaschek, D., Hug, S., Kreutz, C., Harms, B. D., Theis, F. J., Klingmüller, U., and Timmer, J. (2013). Lessons learned from quantitative dynamical modeling in systems biology. *PLoS ONE*, 8(9):e74335.

Rodriguez-Fernandez, M., Egea, J., and Banga, J. (2006). Novel metaheuristic for parameter estimation in nonlinear dynamic biological systems. *BMC Bioinformatics*, 7. cited By 159.

Villaverde, A. F., Froehlich, F., Weindl, D., Hasenauer, J., and Banga, J. R. (2018). Benchmarking optimization methods for parameter estimation in large kinetic models. *Bioinformatics*, page bty736.
