# Supplementary material for: Parameter estimation in models of biological oscillators: an automated regularised estimation approach
Source: BMC Bioinformatics. 2019 Feb 15;20:82. doi: 10.1186/s12859-019-2630-y (PMC6377730; doi:10.1186/s12859-019-2630-y)
Supplement: Supplementary file 2 — Critical comparison of optimisation solvers. In GEARS, the optimisation problems are solved using the hybrid metaheuristic eSS. A comparison of the eSS global optimisation solver used in GEARS with other competitive local and global optimisation solvers. (PDF 880 kb) [file 12859_2019_2630_MOESM2_ESM.pdf]

Additional file 2 for "Parameter estimation in models of biological oscillators: an automated regularised estimation approach."

Jake Alan Pitt[1,2] and Julio R. Banga[1*]

1 (Bio)Process Engineering Group, Spanish National Research Council, IIM-CSIC, C/Eduardo Cabello 6, 36208, Vigo, Spain
2 RWTH-Aachen University Hospital, Joint Research Centre for Computational Biomedicine (JRC-COMBINE), 52074, Aachen, Germany

*Correspondence should be directed to Prof J. R. Banga at julio@iim.csic.es

S2.1 Critical comparison of optimisation solvers

In GEARS, the optimisation problems are solved using the hybrid metaheuristic eSS. In order to justify the selection of this method, we present a comparison with several state-of-the-art local and global optimisation solvers.

S2.2 Comparison with the lsqnonlin local solver

The Levenberg-Marquardt [1] algorithm is probably the most well known local optimisation method for nonlinear least squares problems. Here we have used the implementation in the lsqnonlin solver which is part of the Optimisation Toolbox of Matlab.

We have solved the four case studies with a multi-start of lsqnonlin (in order to take into account the influence of initial guesses), and below we compare it with the eSS as implemented in GEARS. For each case study, we present two figures: a comparison of convergence curves (value of cost function versus computation time) and a comparison of the spread of solutions obtained with each solver (shown as frequency histograms).

For all these case studies, lsqnonlin converged rapidly to local solutions (it only achieved the global solution in 2 runs out of 30 for the FHN problem), while GEARS always converged to the global solution with excellent performance.

These results clearly show the need of using gobal optimisers for calibrating oscillators models due to the multimodality of the associated estimation problems.

S2.2.1 FHN results

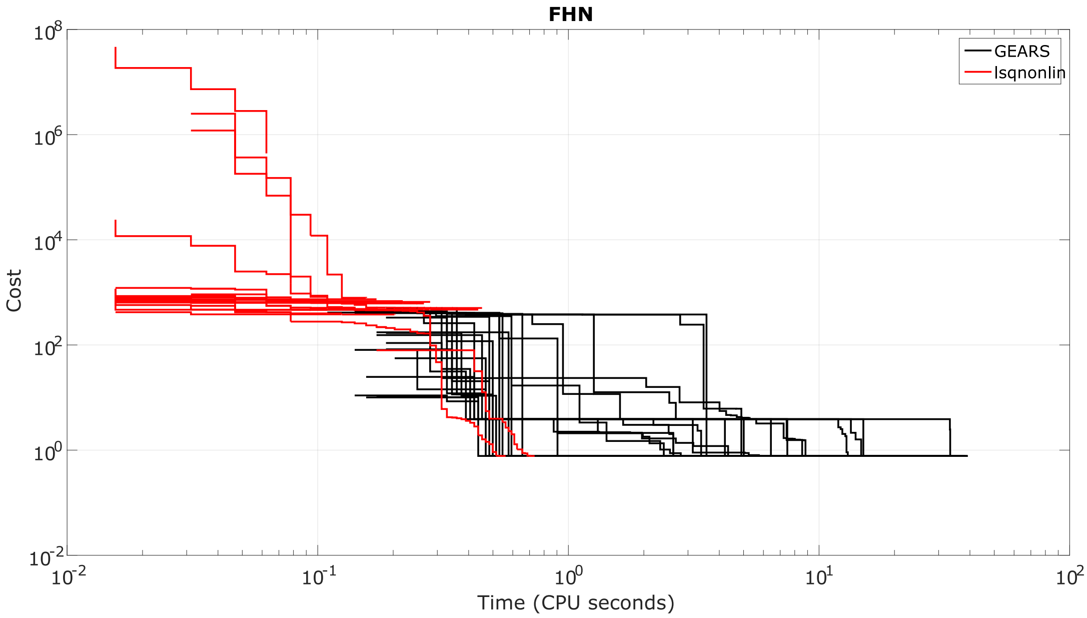

Figure S2.1: Convergence curves for 30 runs of `lsqnonlin` and `GEARS` (without regularisation) for the FHN problem.

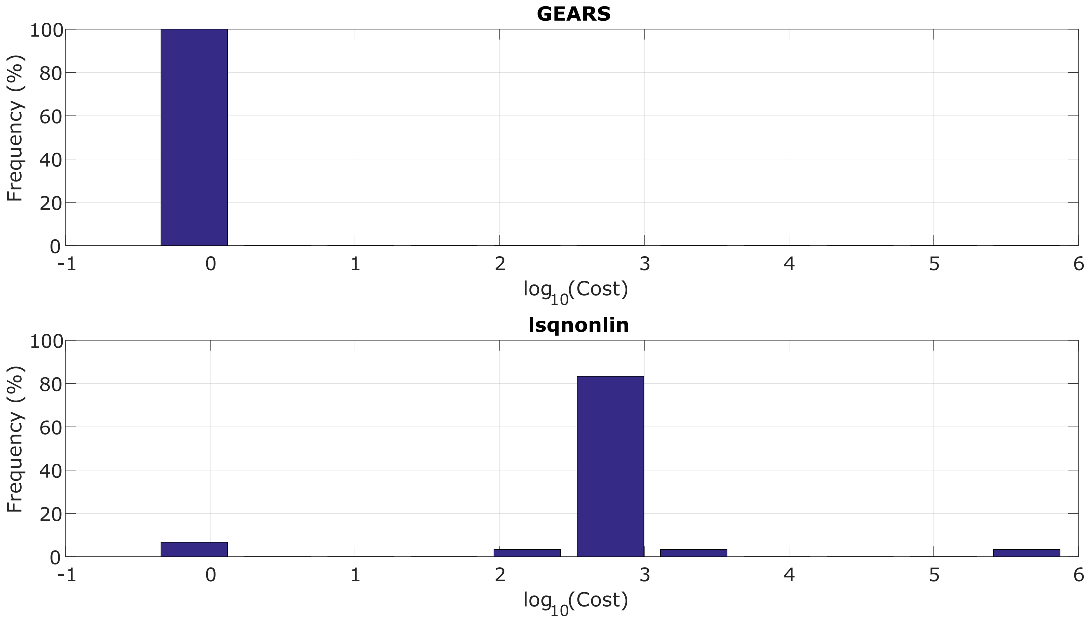

Figure S2.2: Histograms showing the final costs achieved by `lsqnonlin` and `GEARS` (without regularisation) solutions in 30 runs for the FHN problem.

S2.2.2 GO results

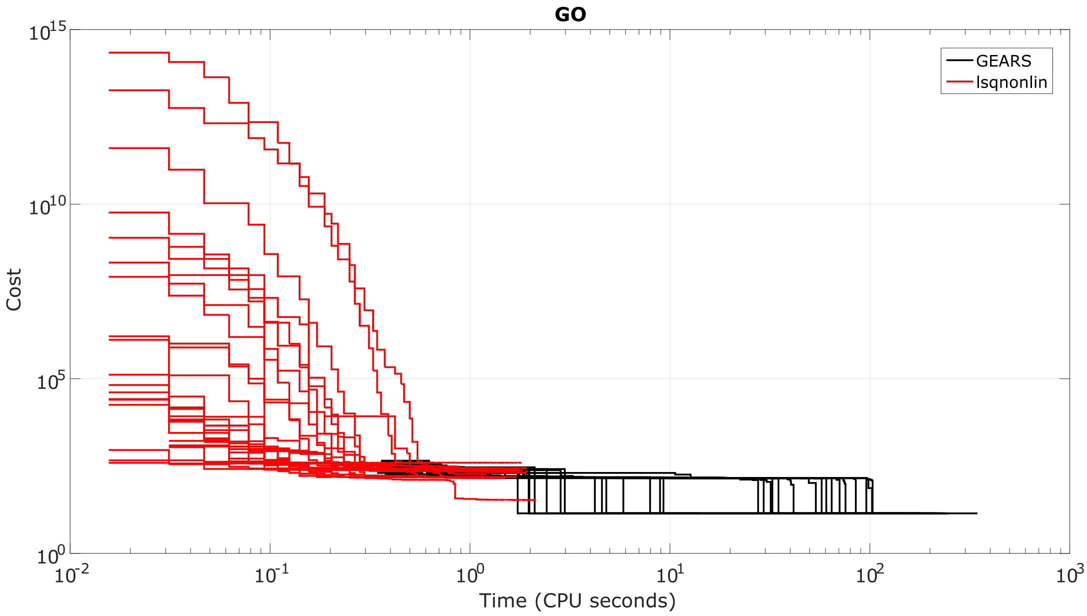

Figure S2.3: Convergence curves for 30 runs of `lsqnonlin` and `GEARS` (without regularisation) for the GO problem.

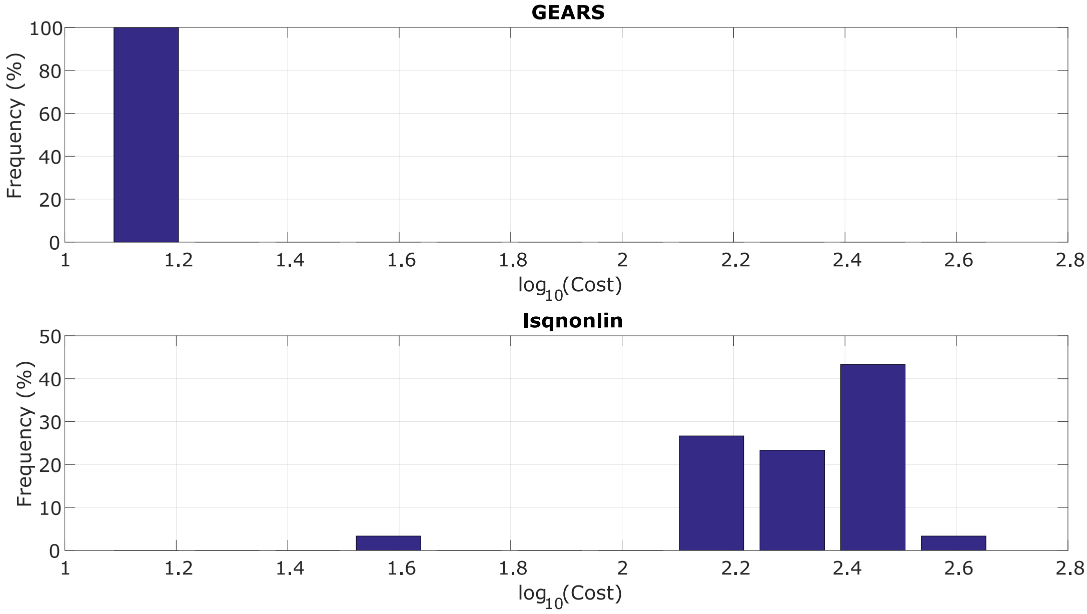

Figure S2.4: Histograms showing the final costs achieved by `lsqnonlin` and `GEARS` (without regularisation) in 30 runs for the GO problem.

S2.2.3 RP result

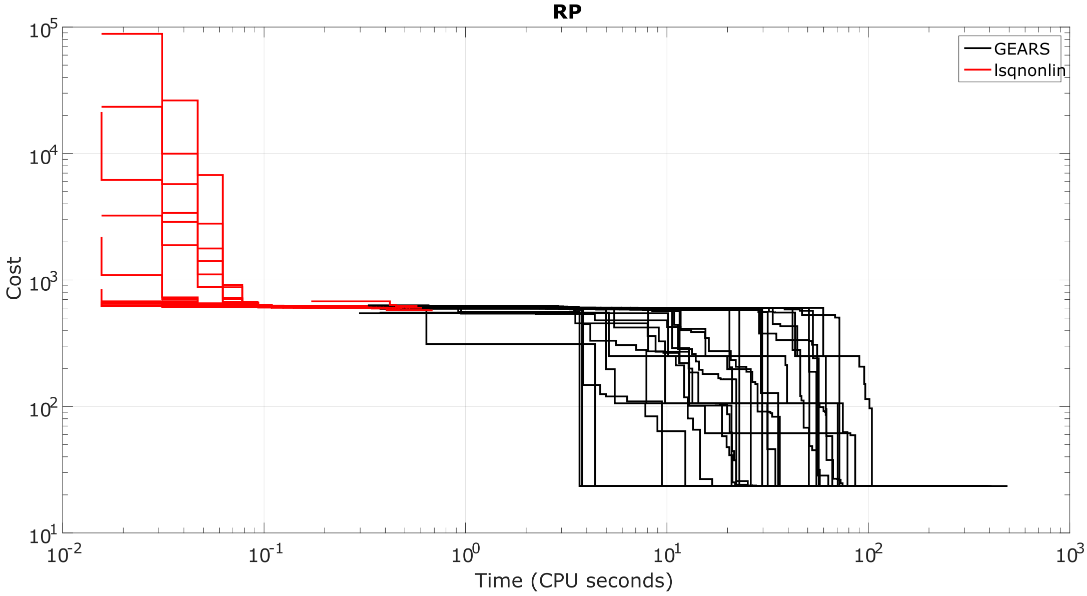

Figure S2.5: Convergence curves for 30 runs of `lsqnonlin` and `GEARS` (without regularisation) for the RP problem.

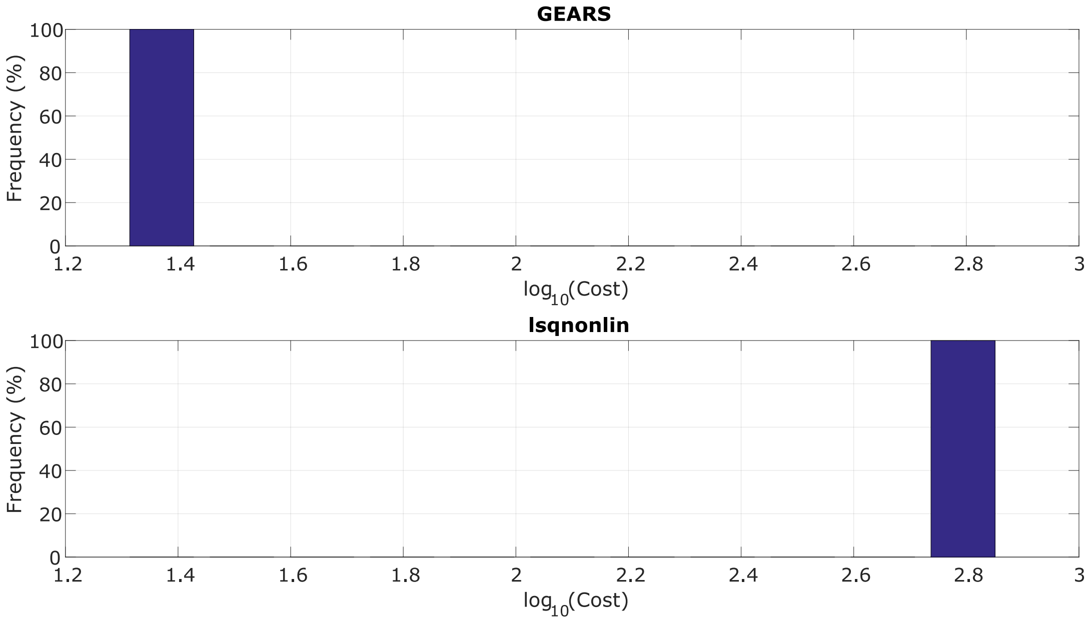

Figure S2.6: Histograms showing the final costs achieved by `lsqnonlin` and `GEARS` (without regularisation) in 30 runs for the RP problem.

S2.2.4 EO results

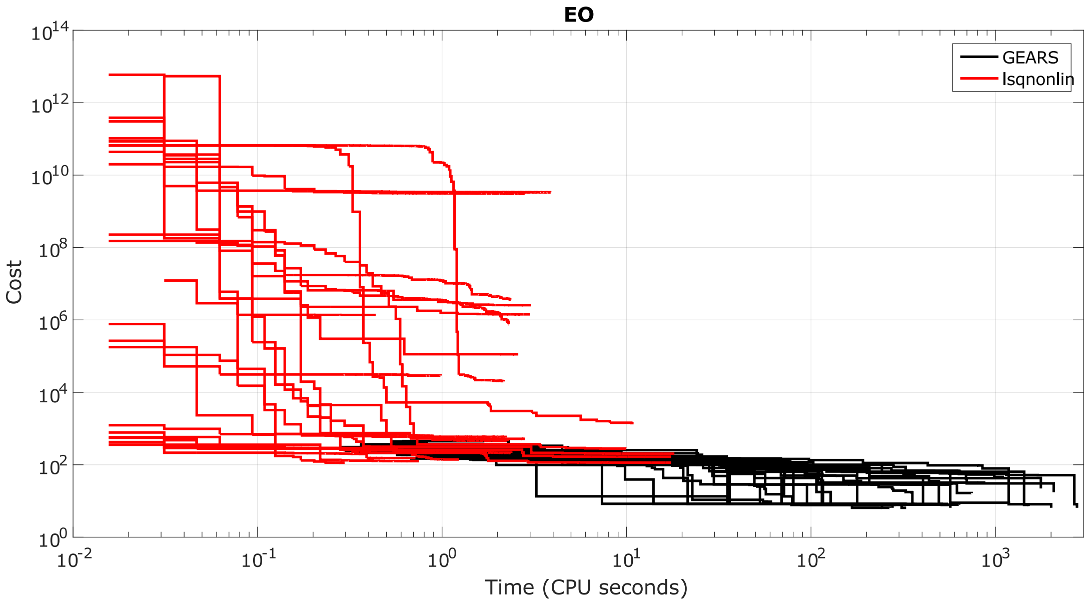

Figure S2.7: Convergence curves for 30 runs of `lsqnonlin` and `GEARS` (without regularisation) for the EO problem.

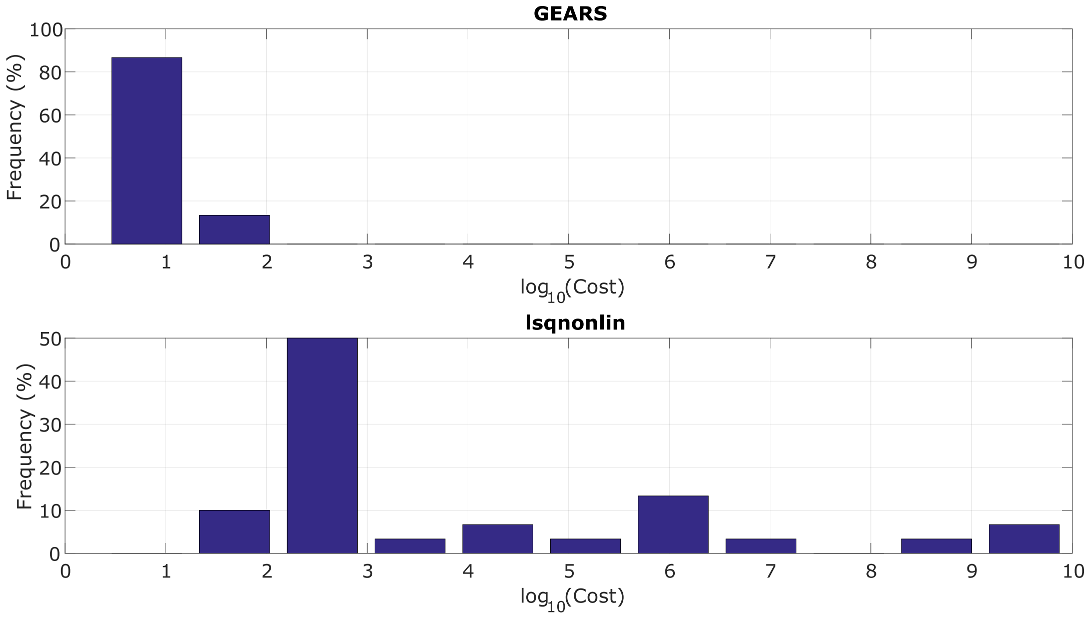

Figure S2.8: Histograms showing the final costs achieved by `lsqnonlin` and `GEARS` (without regularisation) in 30 runs for the RP problem.

S2.3 Comparison with other heuristic global optimisation solvers

In order to illustrate the advantages of `eSS`, the global optimisation heuristic used in `GEARS`, over other competitive global solvers (such as certain types of genetic algorithms, GAs, and evolution strategies, ES), we have performed a comparison with 3 state-of-the-art methods, namely:

- Differential Evolution (DE) [2], a method closely related with GAs and one of the most cited global optimisation methods of all time (near 20,000 citations at the time of writing).
- Stochastic Ranking Evolution Strategy (SRES) [3]: an evolution strategy (also closely related to GAs) and the best performer in several previous comparisons in this field. It is also highly cited (almost 1600 citations).
- GLOBALm [4]: a different type of global optimiser, based on multistart clustering global search.

The above methods were selected because: (i) these methods are widely used and highly cited, (ii) they have been top performers in many published comparisons, and (iii) there are public software implementations available so the results are reproducible.

We solved all the case studies in our manuscript with the above methods and we present below the detailed results in terms of convergence curves and frequency histrograms of the final solutions found by each solver. The conclusions from this comparison are clear: `GEARS` (using `eSS`) clearly outperforms all the above heuristics, both in terms of performance and robustness. These results, obtained for the biological oscillators considered in our study, are in agreement with other recent comparative assessment studies [5] regarding parameter estimation in complex models of biological systems.

S2.3.1 FHN results

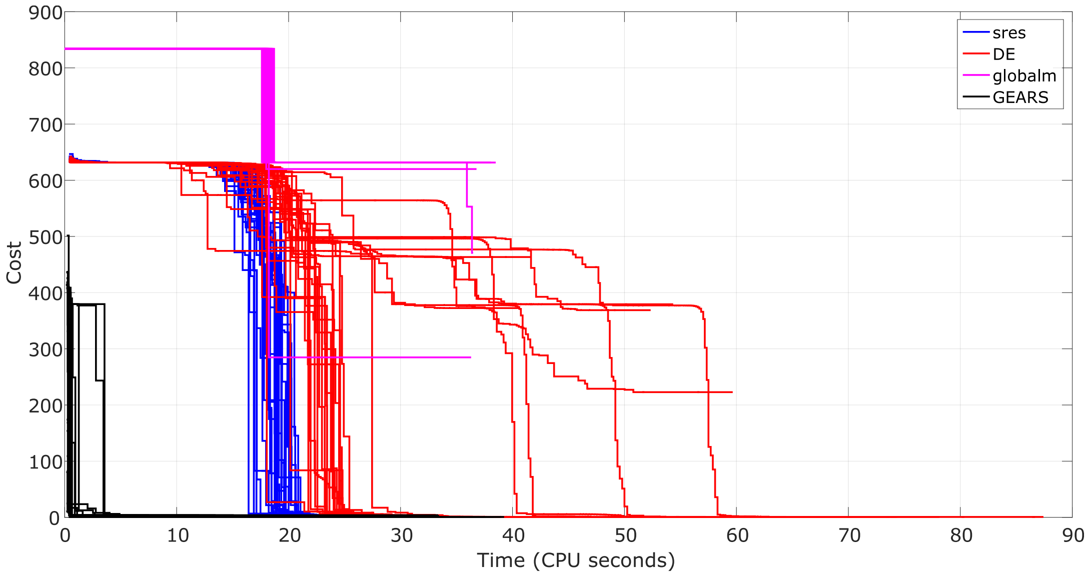

Figure S2.9: Convergence curves comparing the performance of the different global solvers for 30 runs of the FHN case.

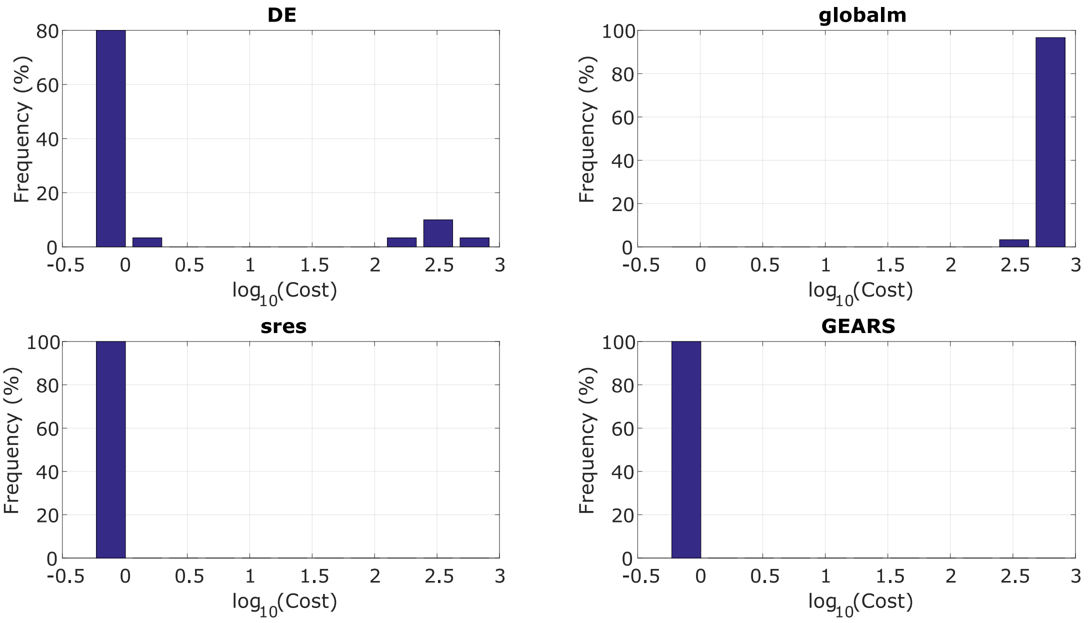

Figure S2.10: Histograms showing the final costs achieved in 30 runs of the different methods for the FHN problem.

S2.3.2 GO results

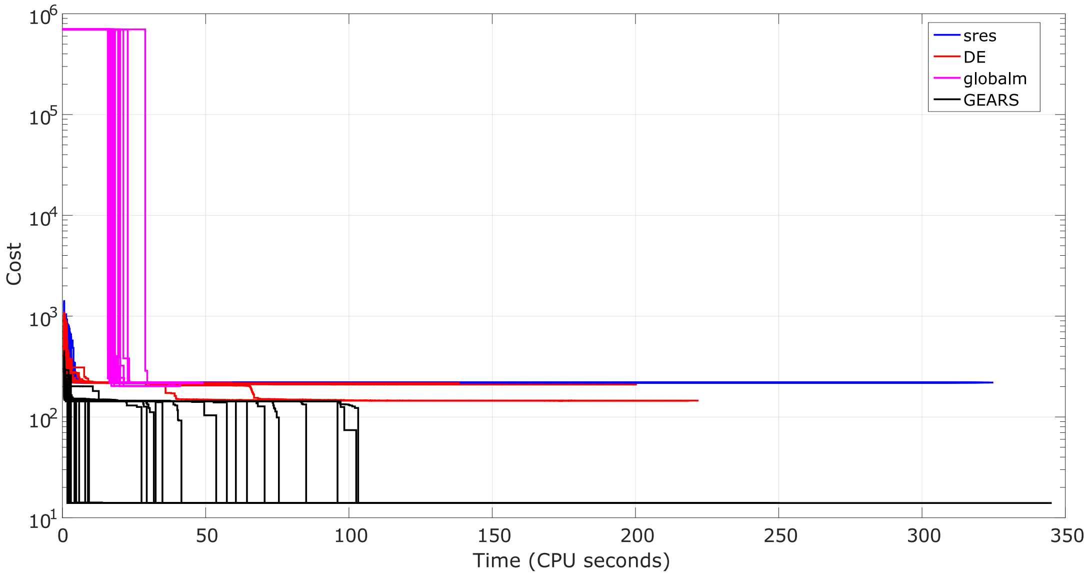

Figure S2.11: Convergence curves comparing the performance of the different global solvers for 30 runs of the GO problem.

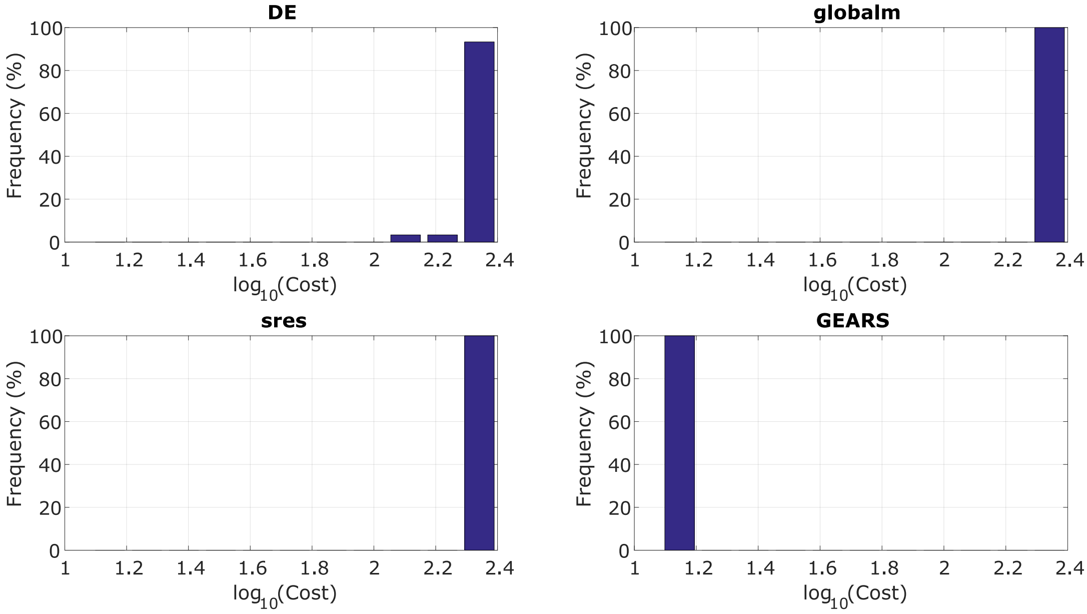

Figure S2.12: Histograms showing the final costs achieved in 30 runs of the different methods for the GO problem.

S2.3.3 RP results

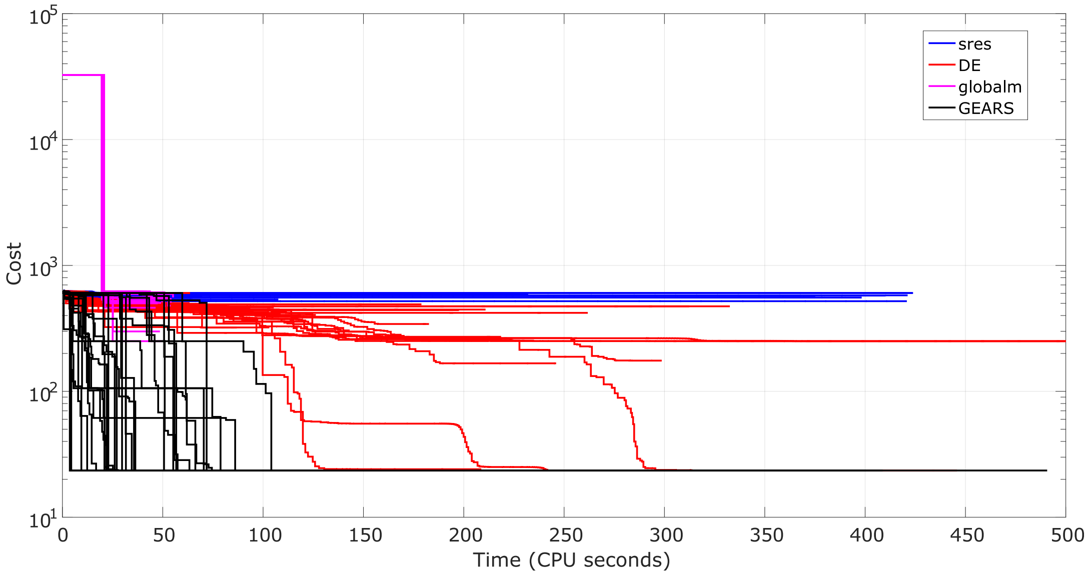

Figure S2.13: Convergence curves comparing the performance of the different global solvers for 30 runs of the the RP case.

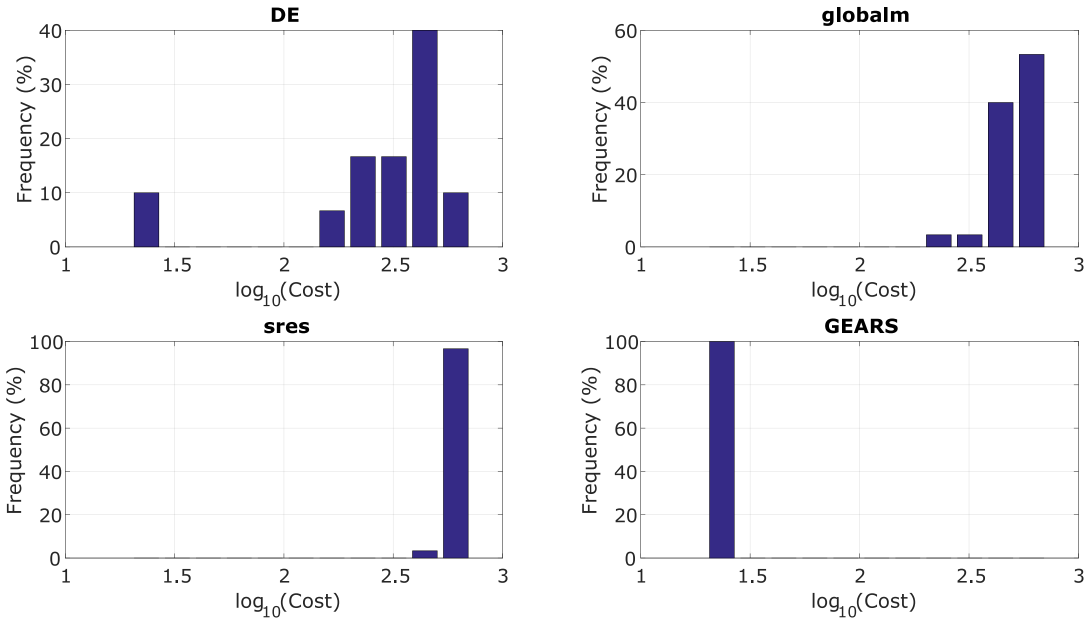

Figure S2.14: Histograms showing the final costs achieved in 30 runs of the different methods for the RP problem.

S2.3.4 EO results

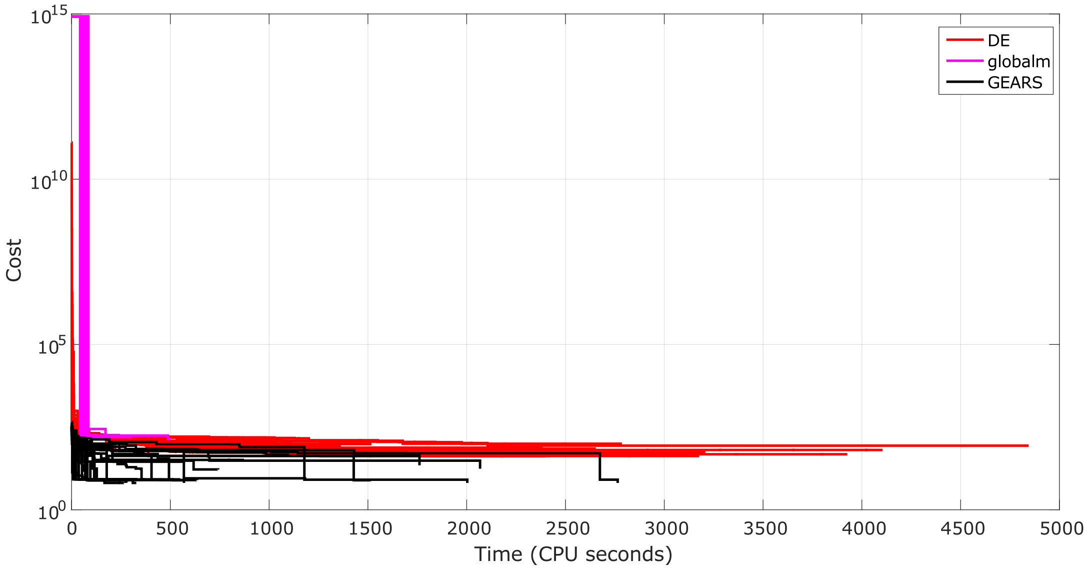

Figure S2.15: Convergence curves comparing the performance of the different global solvers for 30 runs of the EO problem. Results from `SRES` are not shown since it failed in this problem.

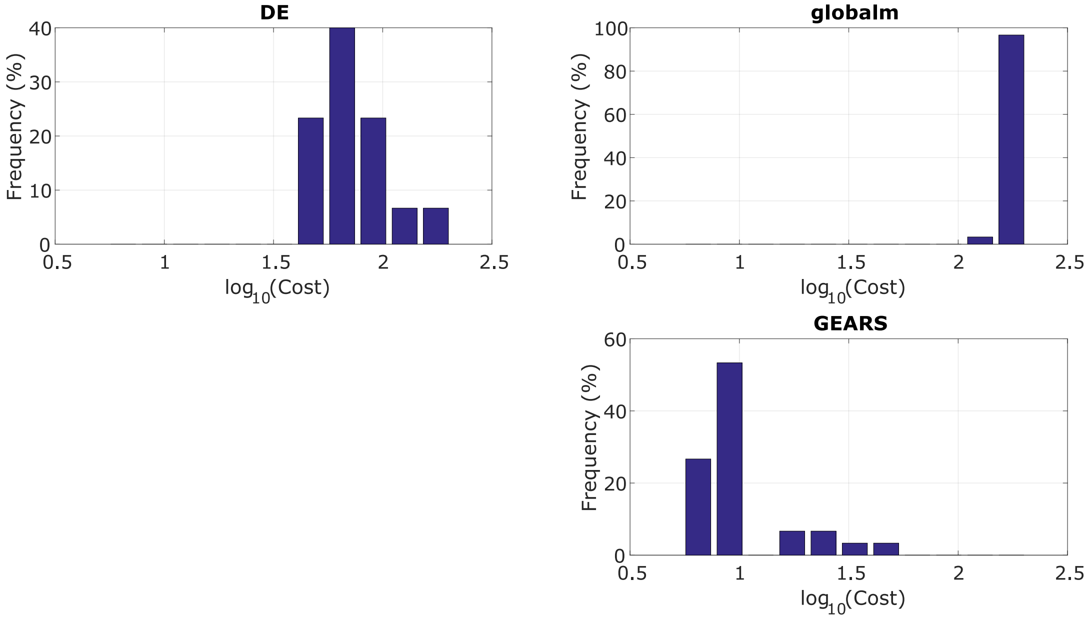

Figure S2.16: Histograms showing the final costs achieved in 30 runs of the different methods for the EO problem. Results from `SRES` are not shown since it failed in this problem.

References

[1] Jorge J Moré. The levenberg-marquardt algorithm: implementation and theory. In *Numerical analysis*, pages 105–116. Springer, 1978.

[2] R. Storn and K. Price. Differential evolution - a simple and efficient heuristic for global optimization over continuous spaces. *Journal of Global Optimization*, 11(4):341–359, 1997. cited By 11219.

[3] T.P. Runarsson and X. Yao. Stochastic ranking for constrained evolutionary optimization. *IEEE Transactions on Evolutionary Computation*, 4(3):284–294, 2000. cited By 1059.

[4] Tibor Csendes, László Pál, J. Oscar H. Sendín, and Julio R. Banga. The global optimization method revisited. *Optimization Letters*, 2(4):445–454, Aug 2008.

[5] Alejandro F Villaverde, Fabian Froehlich, Daniel Weindl, Jan Hasenauer, and Julio R Banga. Benchmarking optimization methods for parameter estimation in large kinetic models. *Bioinformatics*, page bty736, 2018.
