# Supplementary material for: Parameter estimation in models of biological oscillators: an automated regularised estimation approach
Source: BMC Bioinformatics. 2019 Feb 15;20:82. doi: 10.1186/s12859-019-2630-y (PMC6377730; doi:10.1186/s12859-019-2630-y)
Supplement: Supplementary file 3 — Detailed results for the Fitzhugh-Nagumo, Repressilator and Enzymatic Oscillator problems. This file contains tables and figures showing the detailed results for the Fitzhugh-Nagumo, Repressilator and Enzymatic Oscillator problems. (PDF 1680 kb) [file 12859_2019_2630_MOESM3_ESM.pdf]

Additional file 3 for "Parameter estimation in models of biological oscillators: an automated regularised estimation approach."

Jake Alan Pitt[1,2] and Julio R. Banga[1*]

1 (Bio)Process Engineering Group, Spanish National Research Council, IIM-CSIC, C/Eduardo Cabello 6, 36208, Vigo, Spain
2 RWTH-Aachen University Hospital, Joint Research Centre for Computational Biomedicine (JRC-COMBINE), 52074, Aachen, Germany

[*]Correspondence should be directed to Prof J. R. Banga at julio@iim.csic.es

S3.1 FHN case study results

Detailed results for the FHN problem can be found here.

Table S3.1: FHN case study: summary of the regularised results for the fit to the first fitting data set.

Parameter	Value	Confidence (95%)	Coeff of variation (%)	Bounds status
a	0.1069	± 0.0129	6.1732	bounds not active
b	0.6230	± 0.0551	4.5130	bounds not active
g	2.8490	± 0.1528	2.7370	bounds not active

Table S3.2: FHN case study: NRMSE values for the fitting for each fitting data set, with and without regularisation.

	Regularised	Non-regularised
Fitting set 1	0.25758	0.10946
Fitting set 2	0.15195	0.13496
Fitting set 3	0.13739	0.10707
Fitting set 4	0.36033	0.35877
Fitting set 5	0.15546	0.15081
Fitting set 6	0.33357	0.19166
Fitting set 7	0.16416	0.14013
Fitting set 8	0.14951	0.14013
Fitting set 9	0.33509	0.23065
Fitting set 10	0.14415	0.092268

Table S3.3: FHN case study: NRMSE values for the cross-validation for each regularised fit to the fitting data. Here, CV denotes cross-validation data set and F denotes fitting data set.

	F 1	F 2	F 3	F 4	F 5	F 6	F 7	F 8	F 9	F 10
All CV	1.4054	0.91342	1.568	1.4546	1.2945	1.1086	0.86025	0.86489	1.1237	1.1708
CV 1	0.28611	0.26098	0.36268	0.2937	0.33965	1.488	0.43269	0.3478	0.86801	0.55886
CV 2	0.36751	0.26046	0.80114	0.72955	0.28107	1.0501	0.26559	0.25614	0.25908	0.25826
CV 3	3.0809	1.1165	1.714	1.6787	2.638	0.43453	0.73987	0.89282	1.771	2.0556
CV 4	0.34174	0.26721	1.6694	1.1571	1.1178	1.1642	0.22251	0.24208	0.73597	0.60221
CV 5	0.20409	0.59013	1.1545	0.97321	0.55936	0.29394	0.5212	0.55169	0.43116	0.26306
CV 6	0.41283	0.26985	0.47756	0.41991	0.28595	0.45085	0.8041	0.53152	0.22888	0.25147
CV 7	0.42833	0.32626	0.59312	0.50113	0.49843	1.123	0.41085	0.36075	0.65653	0.50676
CV 8	1.768	1.6869	2.8185	2.5564	1.811	1.7919	1.6536	1.6749	1.7928	1.7777
CV 9	2.0913	1.7997	2.3005	2.3232	1.5431	1.2677	1.5819	1.643	1.7503	1.8216
CV 10	1.4248	0.52681	1.7146	1.7539	1.432	1.0294	0.47094	0.46519	1.0868	1.369

Table S3.4: FHN case study: NRMSE values for the cross-validation for each non-regularised fit to the fitting data. Here, CV denotes cross-validation data set and F denotes fitting data set.

	F 1	F 2	F 3	F 4	F 5	F 6	F 7	F 8	F 9	F 10
All CV	1.4487	1.014	1.8203	1.477	1.3133	1.4451	0.91002	0.91003	1.3498	1.2337
CV 1	1.8314	0.20565	1.0443	1.8248	0.41676	1.9123	0.267	0.26699	1.8291	1.5215
CV 2	1.2531	0.28248	1.5143	1.8397	0.27679	1.4588	0.25943	0.25943	1.3418	0.84382
CV 3	1.2542	1.3358	1.8885	1.7733	2.6426	0.96563	1.1034	1.1034	0.49304	1.2277
CV 4	1.1977	0.27866	1.8943	1.3328	1.0492	1.3448	0.26551	0.26551	1.2445	1.1271
CV 5	0.41586	0.62773	1.7784	0.46052	0.49988	0.46931	0.58233	0.58234	0.27744	0.20587
CV 6	1.0829	0.17287	0.60911	1.4717	0.27555	1.3408	0.28431	0.28429	1.1618	0.38201
CV 7	1.77	0.32797	0.78361	1.3261	0.49309	1.9461	0.32668	0.32668	1.7165	1.2299
CV 8	2.088	1.6977	2.8868	0.42949	1.794	1.7651	1.6845	1.6845	1.8181	1.7687
CV 9	1.7618	1.9688	2.4778	1.3499	1.7549	1.2703	1.7922	1.7922	1.5445	1.7259
CV 10	1.0548	1.0038	1.9941	2.0094	1.43	1.3496	0.52789	0.52788	1.1039	1.2681

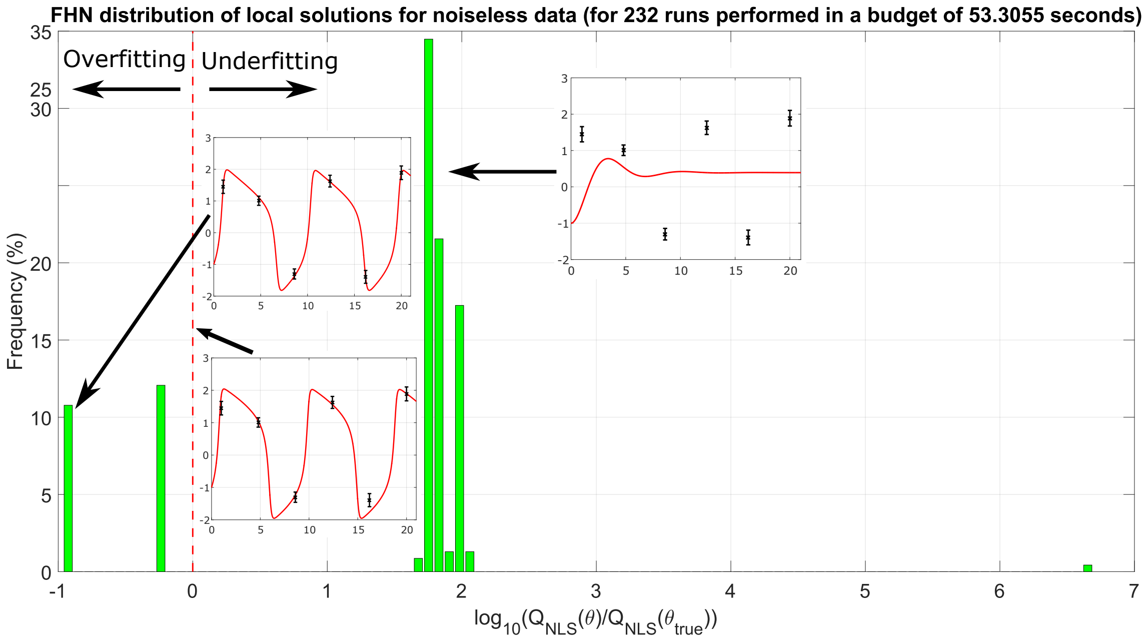

Figure S3.1: FHN case study: distribution of solutions found using the nl2sol local solver with examples of local solutions and overfitting for the first fitting data set.

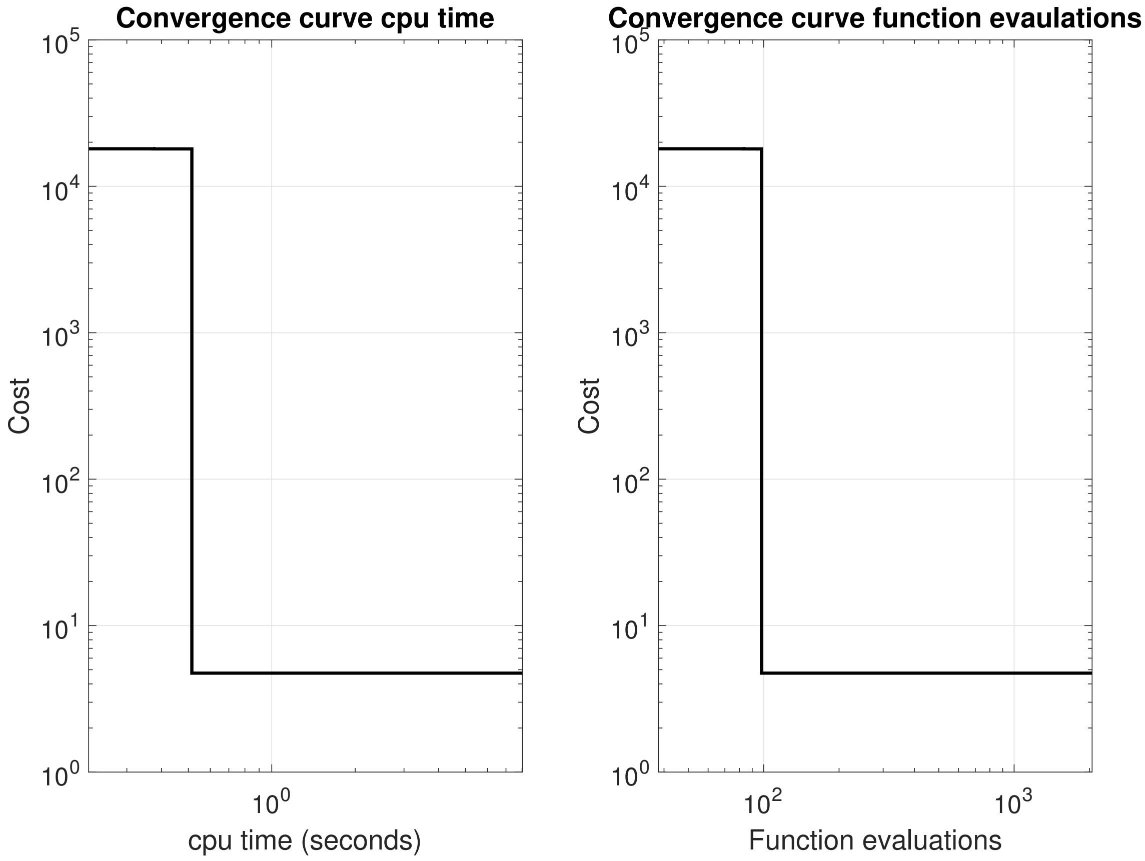

Figure S3.2: FHN case study: convergence curve of the final regularised estimation for the first fitting data set.

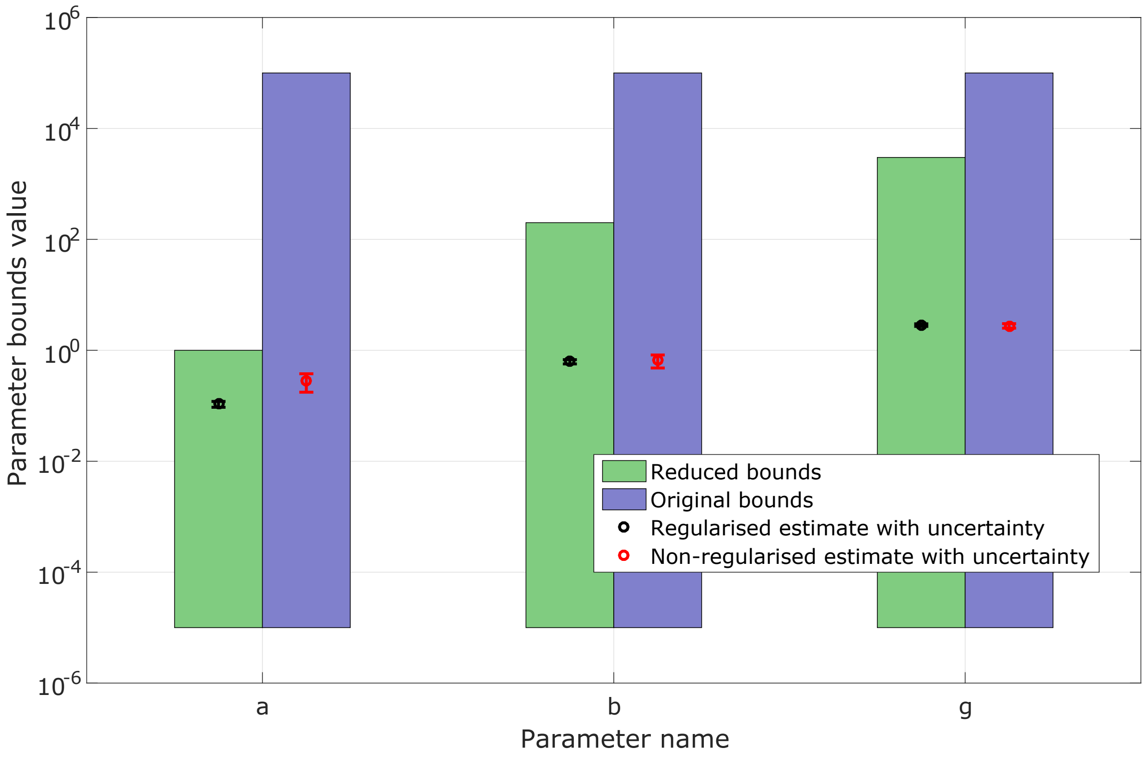

Figure S3.3: FHN case study: reduction in the parameter bounds with the estimated values and their 95% confidence intervals for the first fitting data set.

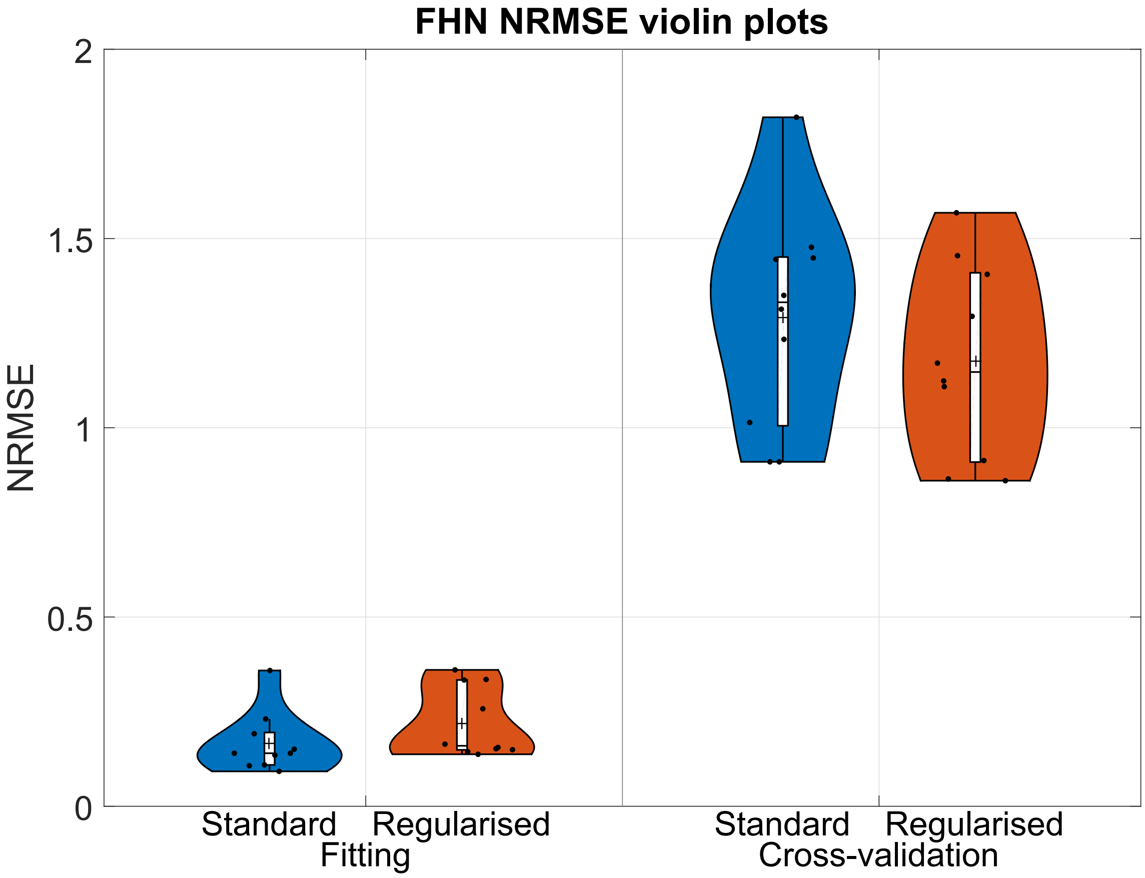

Figure S3.4: FHN case study: violin plots showing the distribution of the NRMSE values for the fit and cross-validation for all the data sets considered, both with and without regularisation.

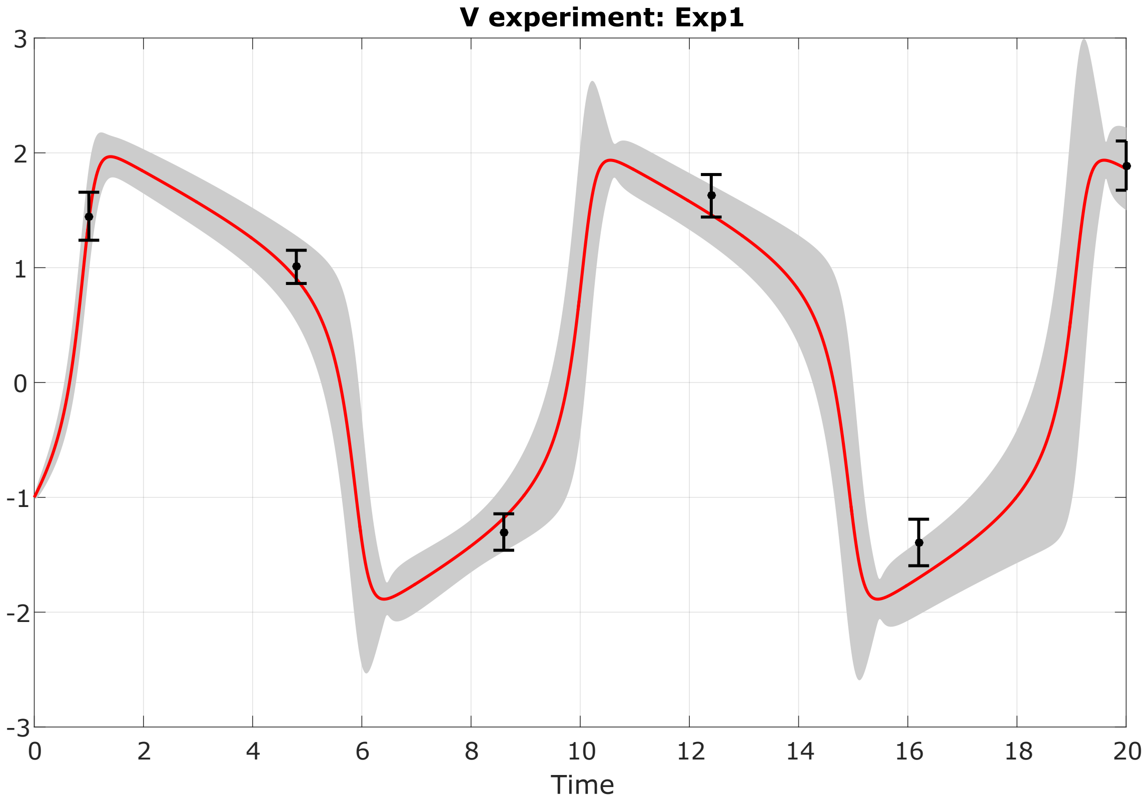

Figure S3.5: FHN case study: final regularised fit with uncertainty intervals for the first fitting data set.

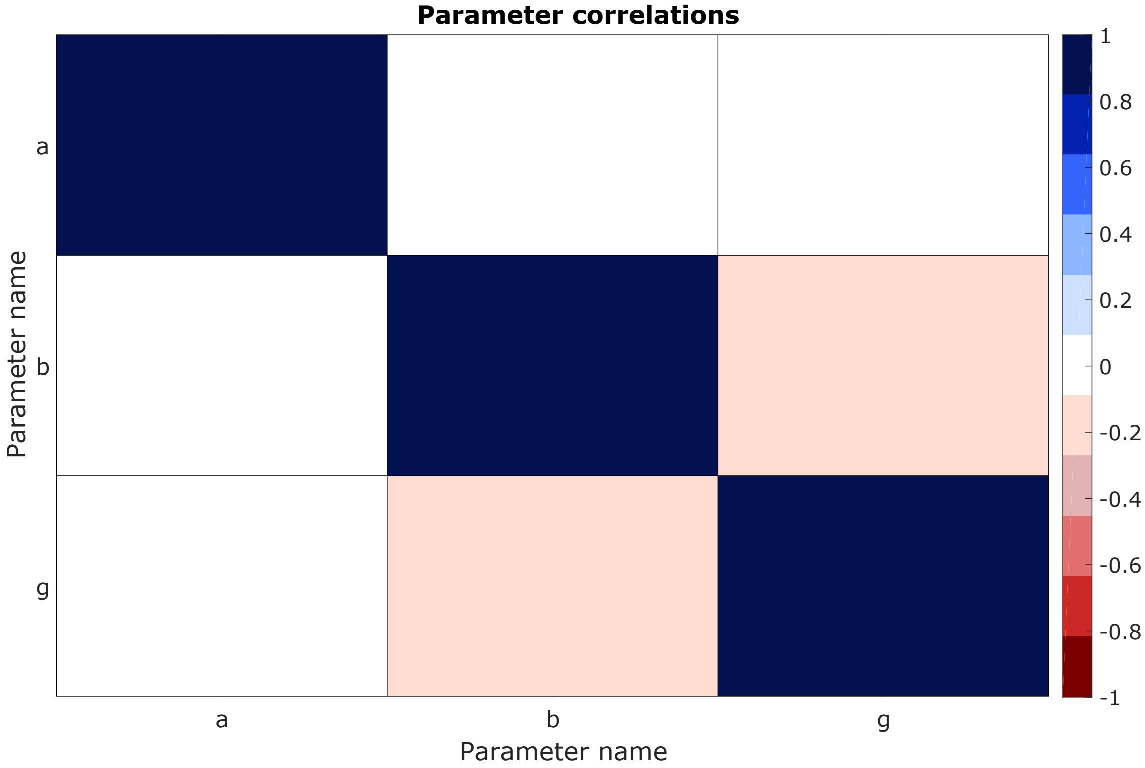

Figure S3.6: FHN case study: parameter correlation matrix for the final estimated regularised solution for the first fitting data set.

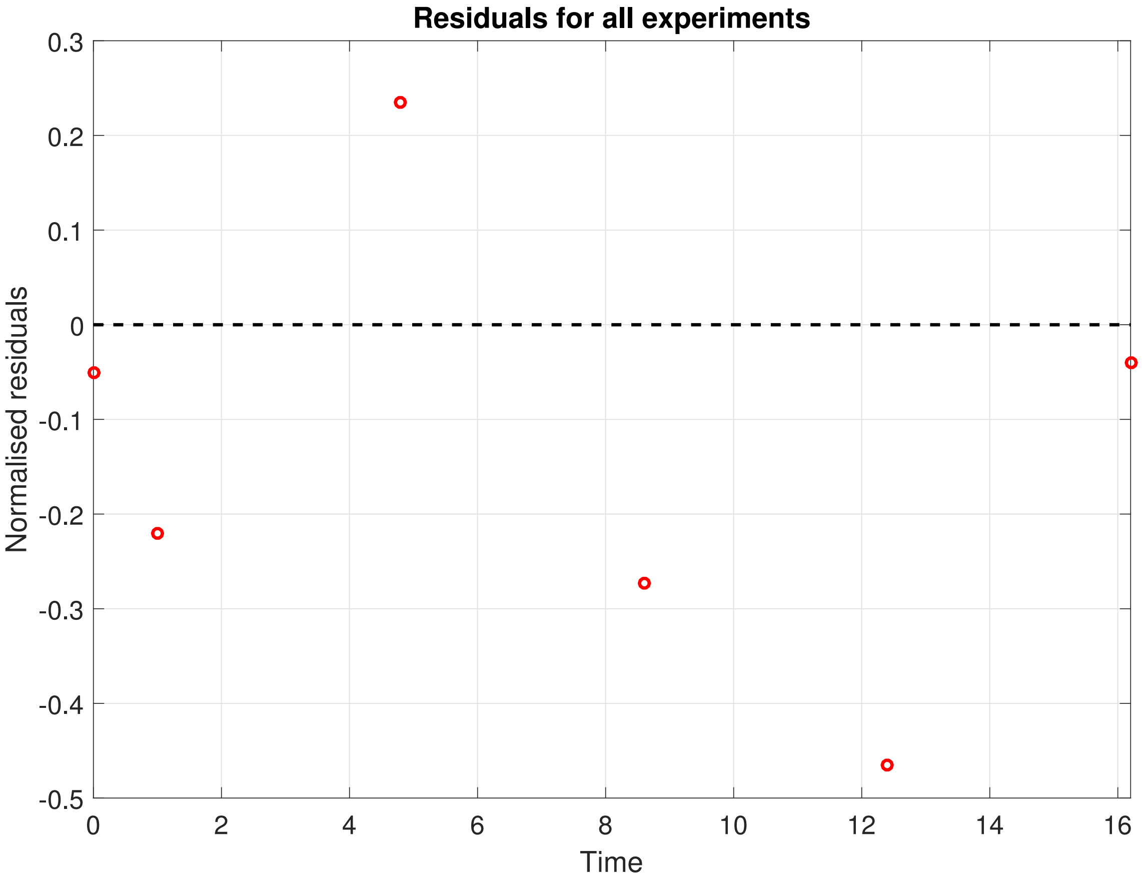

Figure S3.7: FHN case study: normalised residuals for the regularised fit for the first fitting data set.

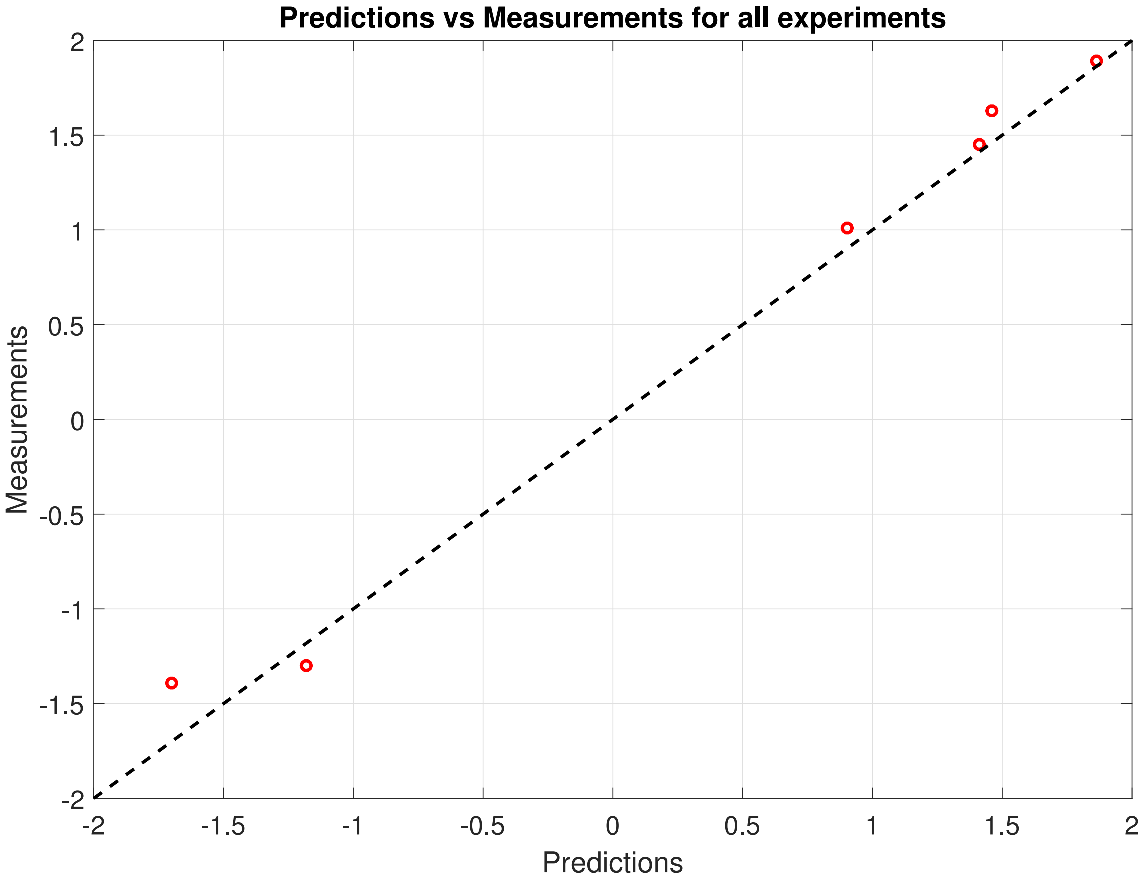

Figure S3.8: FHN case study: predicted versus observed values for the first fitting data set.

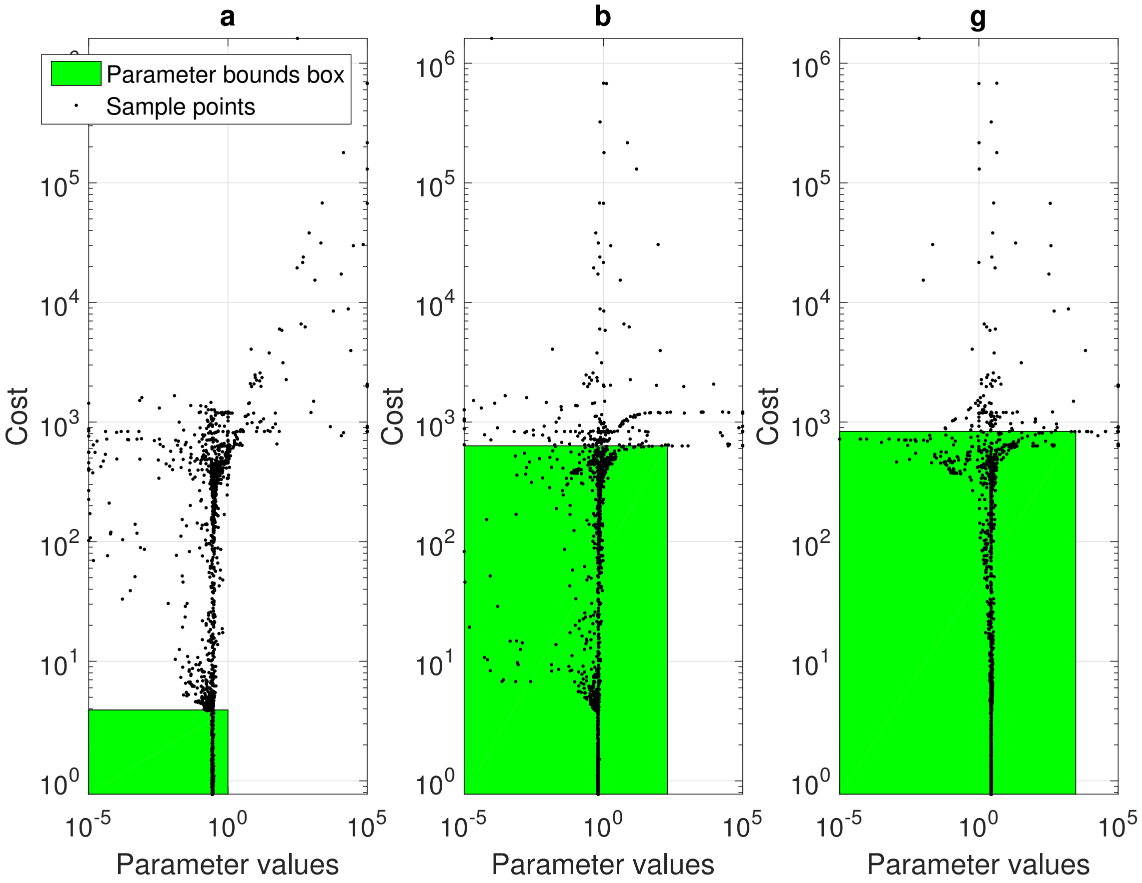

Figure S3.9: FHN case study: sample from the initial estimation with the new parameter bound box, where the height of said box is the cost cut off for the first fitting data set.

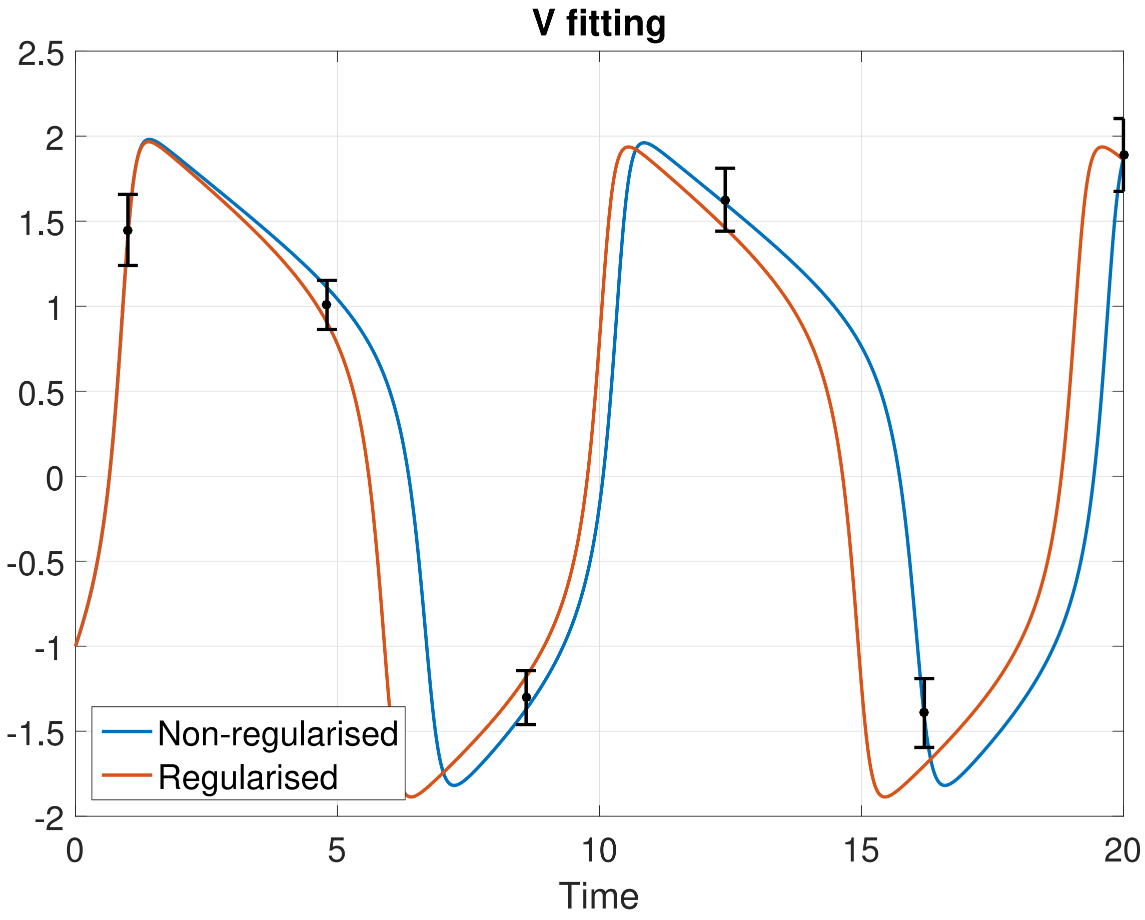

Figure S3.10: FHN case study: comparison of the fits with and without regularisation for the first fitting data set.

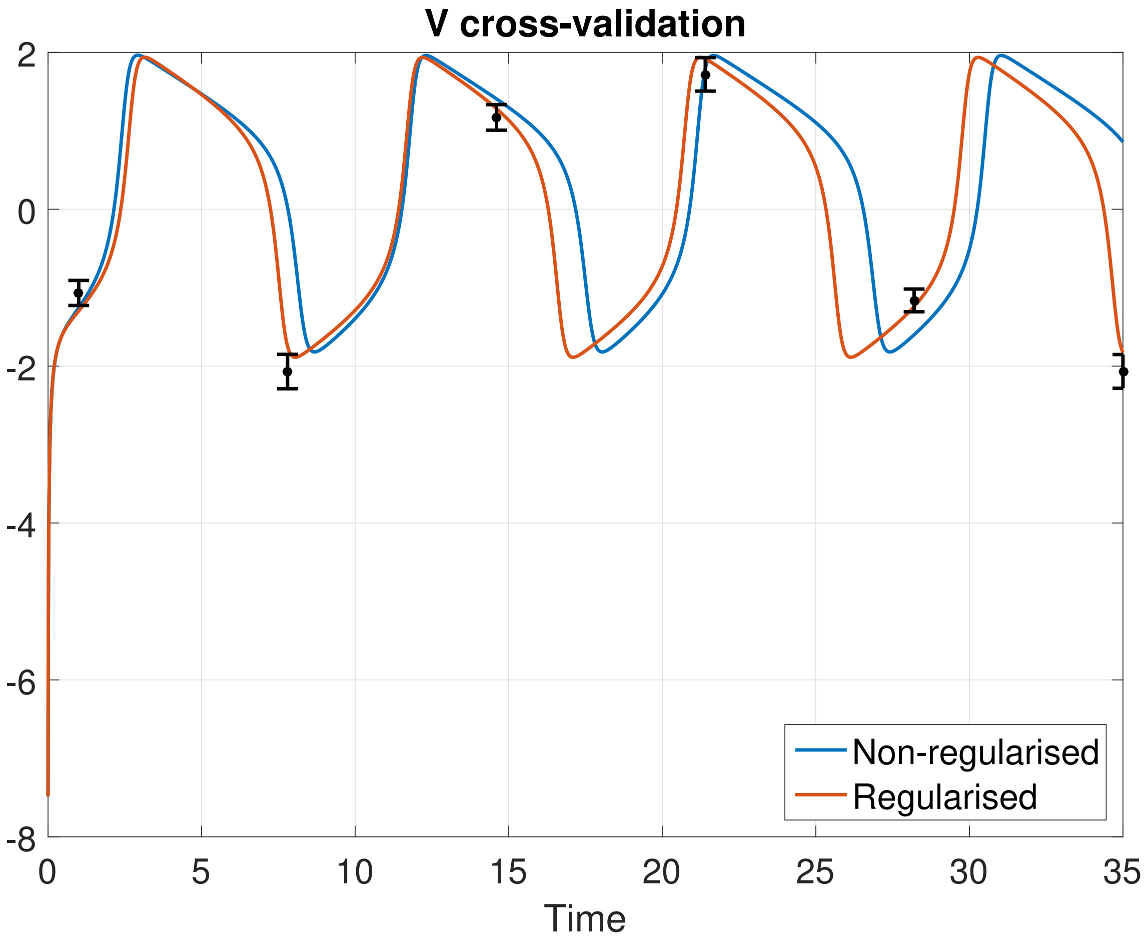

Figure S3.11: FHN case study: comparison of the cross-validation with and without regularisation for the fit to the first fitting data set.

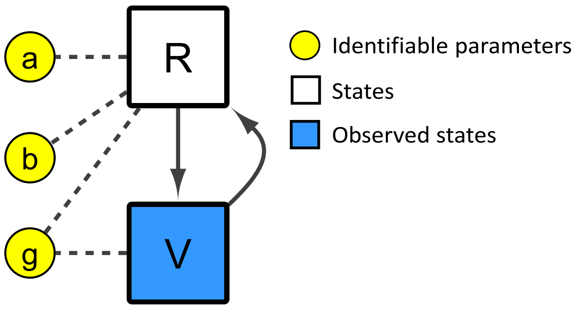

Figure S3.12: FHN case study: results of the VisId analysis performed at the regularised solution for the first fitting data set.

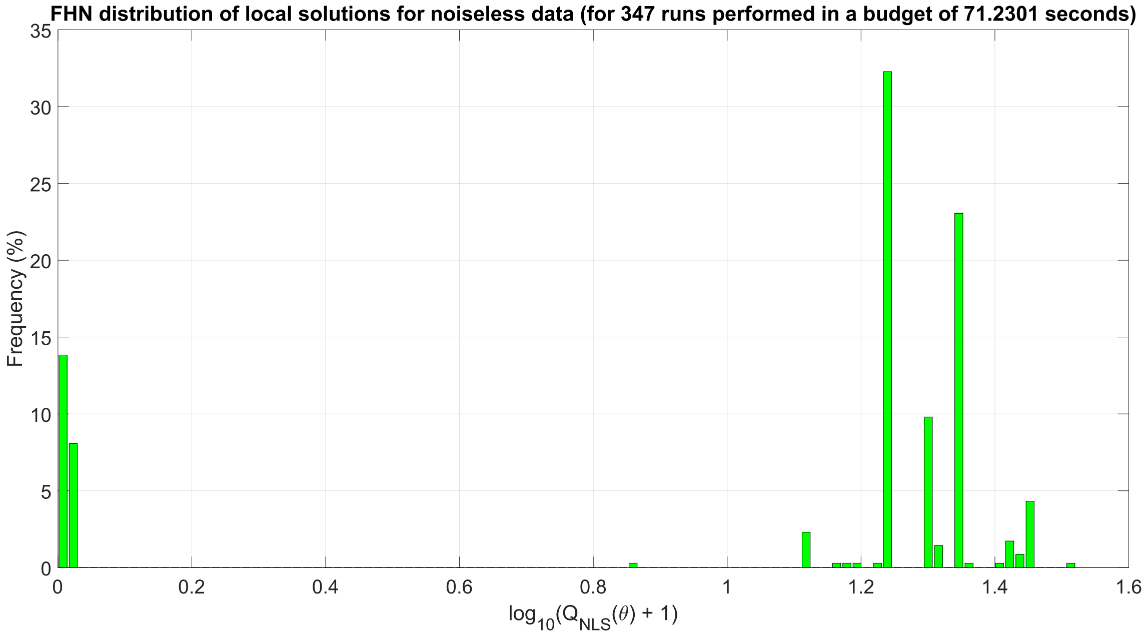

Figure S3.13: FHN case study: distribution of solutions found using the nl2sol local solver fitting to noiseless data.

S3.2 RP problem results

Detailed results for the RP case study can be found here.

Table S3.5: RP case study: summary of the regularised results for the regularised fit to the first fitting data set.

Parameter	Value	Confidence (95%)	Coeff of variation (%)	Bounds status
α_0	1.0000e-03	± 0.0339072	1729.9568	Lower bound active
α	305.8607	± 23.1333	3.8588457	Bounds not active
n	8.7759	± 1.9681	11.441934	Bounds not active
β	0.2979	± 0.0110613	1.8941222	Bounds not active

Table S3.6: RP case study: NRMSE values for the fitting for each fitting data set, with and without regularisation.

	Regularised	Non-regularised
Fitting set 1	0.003058	0.0029454
Fitting set 2	0.0037345	0.0028245
Fitting set 3	0.0028395	0.0020954
Fitting set 4	0.0041512	0.0028835
Fitting set 5	0.0032896	0.0031589
Fitting set 6	0.0026429	0.0022547
Fitting set 7	0.0025042	0.0024369
Fitting set 8	0.0043533	0.0032509
Fitting set 9	0.0025147	0.0024138
Fitting set 10	0.0042353	0.0031773

Table S3.7: RP case study: NRMSE values for the cross-validation for each regularised fit to the fitting data. Here, CV denotes cross-validation data set and F denotes fitting data set.

	F 1	F 2	F 3	F 4	F 5	F 6	F 7	F 8	F 9	F 10
All CV	0.010209	0.011113	0.013345	0.013072	0.0073395	0.006618	0.0067622	0.011761	0.016007	0.014158
CV 1	0.011846	0.010615	0.011641	0.010473	0.011112	0.011585	0.011339	0.011809	0.0046423	0.011804
CV 2	0.003409	0.0051609	0.0055662	0.0055686	0.0042063	0.004572	0.0048925	0.0074889	0.0031253	0.0086631
CV 3	0.0067989	0.01077	0.0075154	0.012043	0.0047154	0.0080207	0.0076951	0.0093117	0.013872	0.011688
CV 4	0.0040396	0.0050636	0.0063911	0.006645	0.0047813	0.0048151	0.0052343	0.008304	0.0031452	0.0096835
CV 5	0.0037767	0.0036489	0.0052823	0.0067779	0.0036562	0.0031405	0.0034838	0.0050485	0.0054189	0.0079453
CV 6	0.0052017	0.0039615	0.0073469	0.011046	0.0040974	0.0034755	0.0037729	0.0058516	0.0045806	0.011809
CV 7	0.013594	0.0063302	0.013315	0.012401	0.012545	0.0059882	0.0094579	0.013114	0.016906	0.013142
CV 8	0.01212	0.026341	0.020987	0.018921	0.0067046	0.0062312	0.0054291	0.020987	0.036561	0.019947
CV 9	0.0043431	0.0051425	0.0058978	0.0059951	0.0048345	0.0049428	0.0052041	0.007158	0.0039455	0.0081923
CV 10	0.020878	0.012829	0.027975	0.025753	0.009826	0.0086195	0.0067658	0.017567	0.025305	0.02682

Table S3.8: RP case study: NRMSE values for the cross-validation for each non-regularised fit to the fitting data. Here, CV denotes cross-validation data set and F denotes fitting data set.

	F 1	F 2	F 3	F 4	F 5	F 6	F 7	F 8	F 9	F 10
All CV	0.013751	0.0063153	0.00884	0.013575	0.013899	0.0078714	0.0059517	0.010741	0.022843	0.015516
CV 1	0.012583	0.011669	0.013604	0.011654	0.013971	0.012114	0.011467	0.012384	0.017198	0.012536
CV 2	0.0040153	0.0041922	0.0040289	0.0039908	0.0044718	0.0040891	0.0041888	0.0041068	0.0049564	0.0063077
CV 3	0.003735	0.0078143	0.0038953	0.003637	0.0067991	0.0041322	0.0062124	0.012375	0.013713	0.018775
CV 4	0.0064884	0.0044918	0.0081186	0.0060726	0.0083841	0.0046418	0.0045809	0.0048579	0.009421	0.0072704
CV 5	0.0068183	0.0030585	0.0038377	0.006389	0.0083726	0.0034079	0.0030915	0.0036191	0.019308	0.0071956
CV 6	0.012703	0.0034079	0.0030831	0.013105	0.012003	0.0039186	0.0033124	0.0041216	0.021459	0.011236
CV 7	0.022215	0.0059678	0.010137	0.022083	0.021136	0.013575	0.0060142	0.013878	0.03472	0.014371
CV 8	0.0074986	0.0051428	0.0058176	0.0055961	0.012635	0.0091876	0.0044004	0.021362	0.032742	0.022216
CV 9	0.013894	0.0047527	0.01791	0.012572	0.016314	0.0048289	0.0047168	0.0047013	0.0157	0.006553
CV 10	0.02664	0.0076313	0.0041814	0.027412	0.022659	0.0099254	0.006878	0.010276	0.035549	0.029736

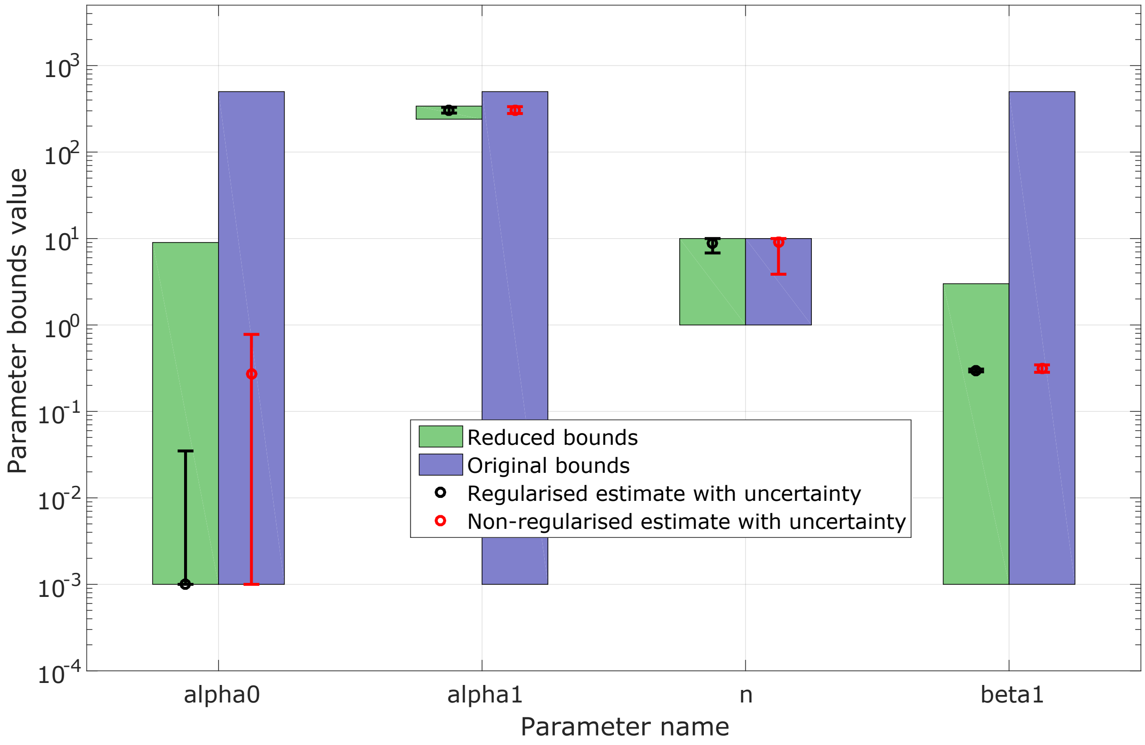

Figure S3.14: RP case study: reduction in the parameter bounds with the estimated values and their 95% confidence intervals for the first fitting data set.

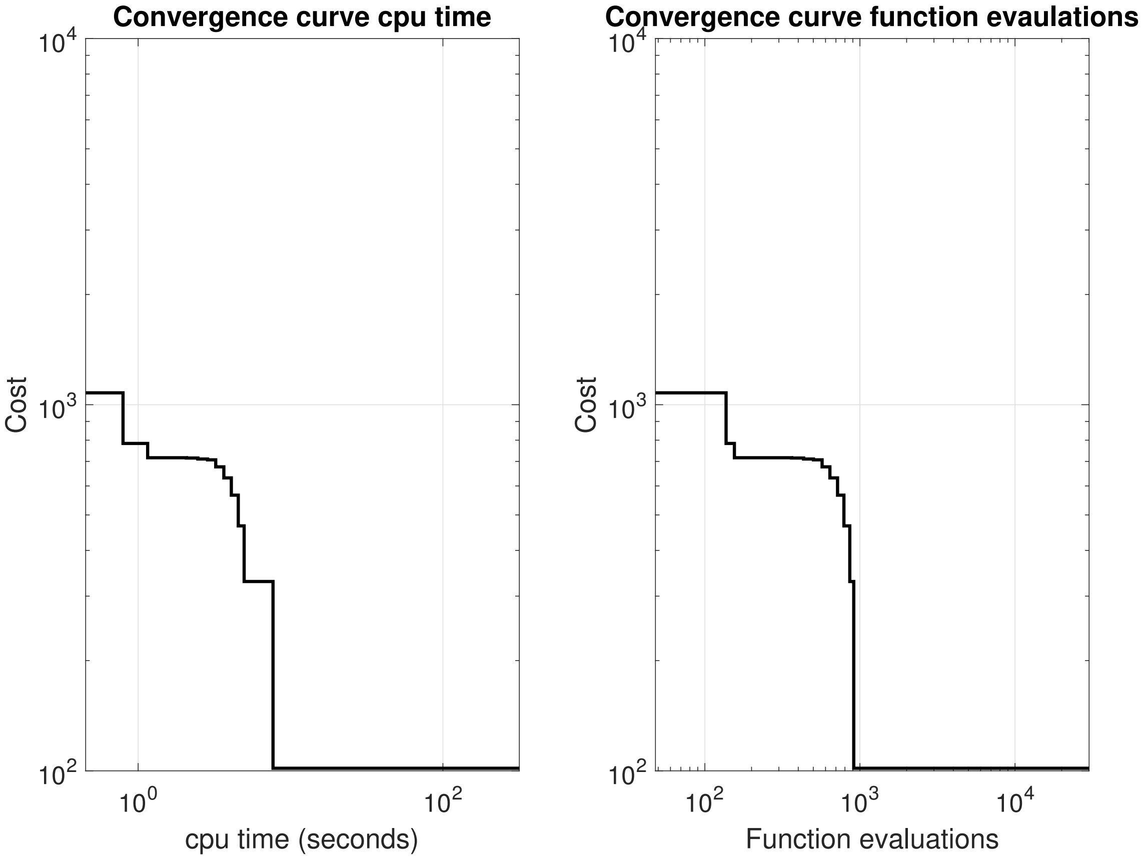

Figure S3.15: RP case study: convergence curve of the final regularised estimation for the first fitting data set.

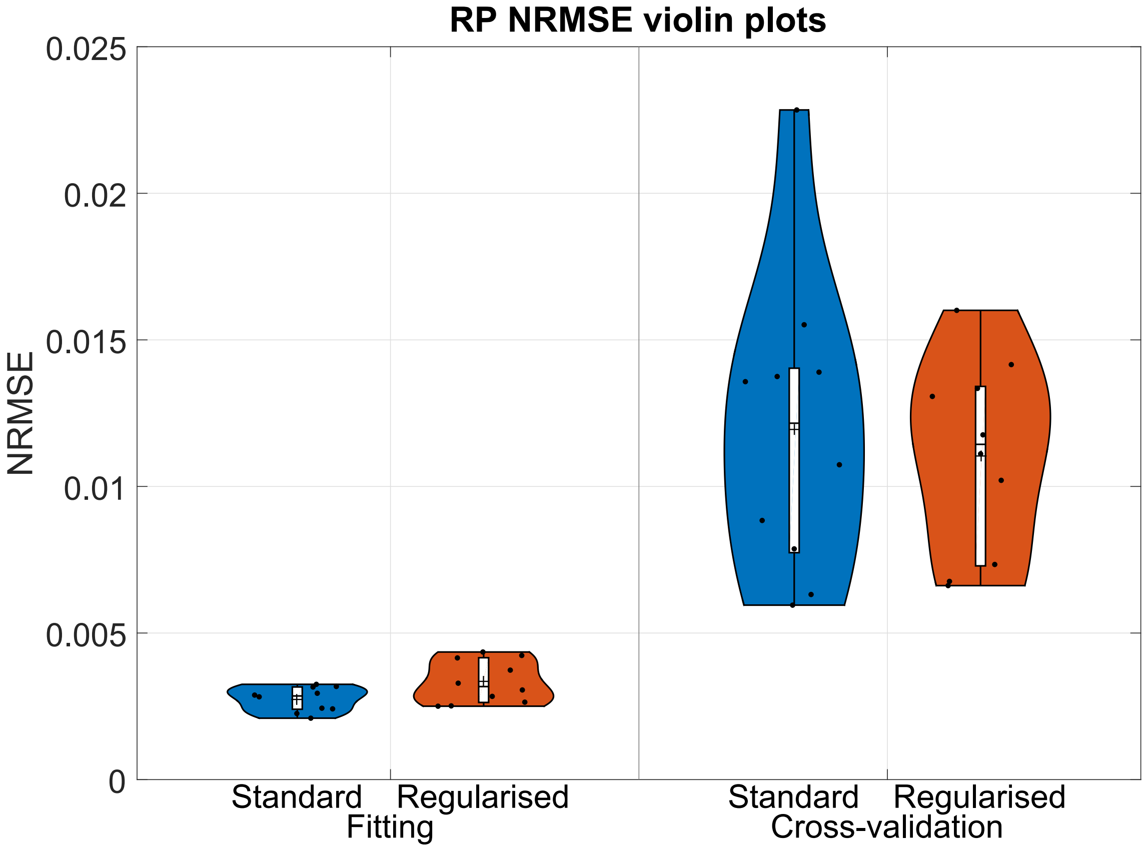

Figure S3.16: RP case study: violin plots showing the distribution of the NRMSE for the fit and cross-validation for all the data sets considered, both with and without regularisation.

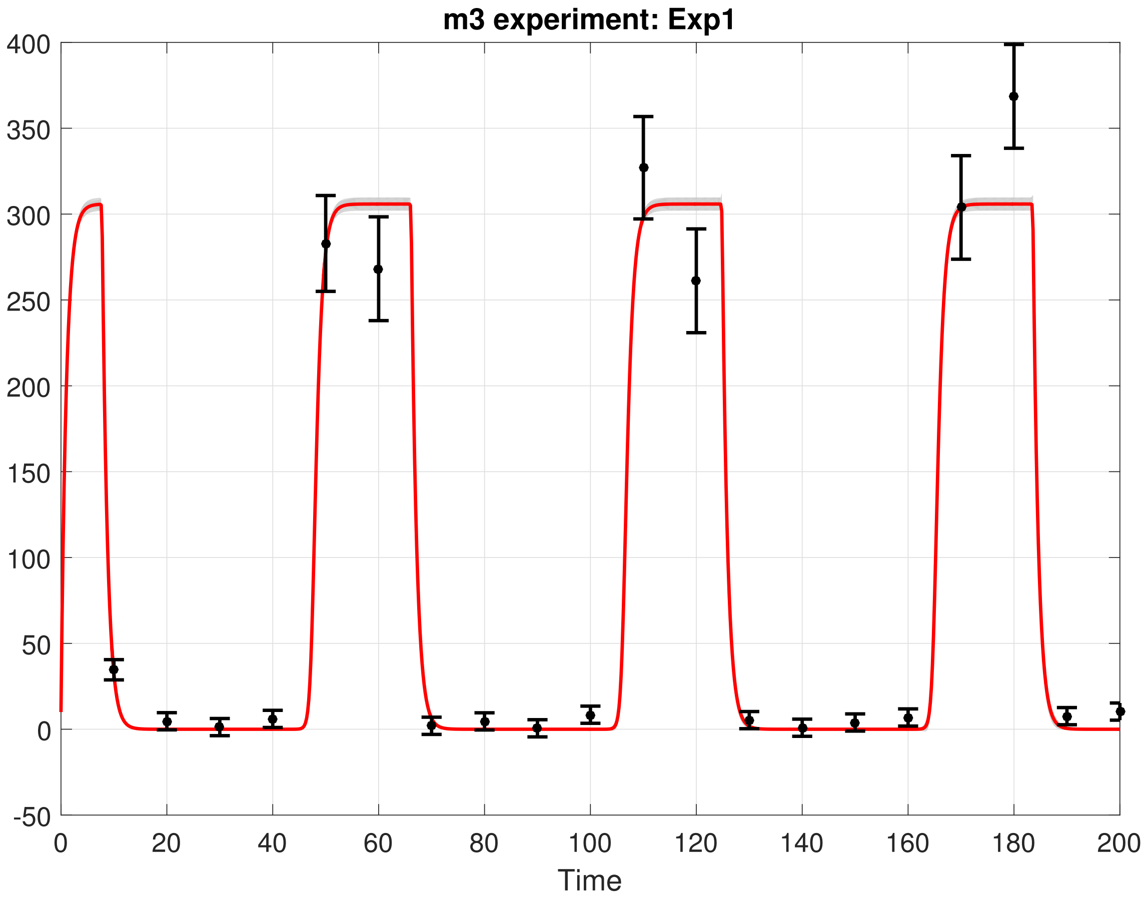

Figure S3.17: RP case study: final regularised fit with uncertainty intervals for the first fitting data set.

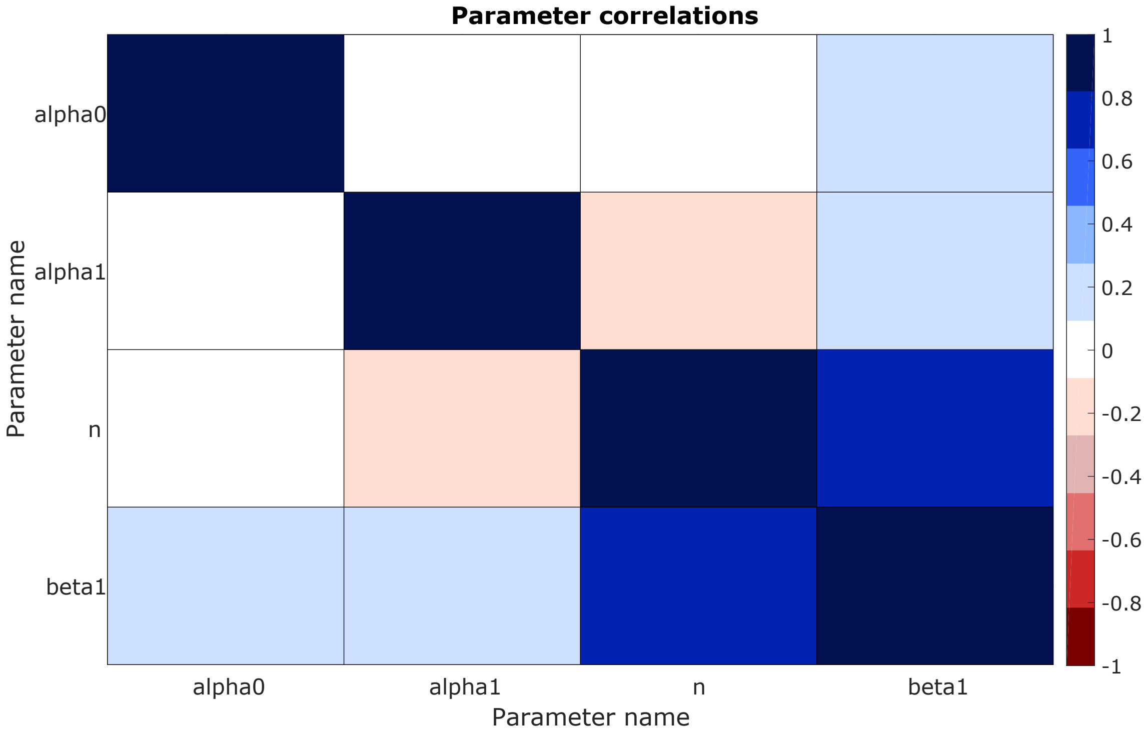

Figure S3.18: RP case study: parameter correlation matrix for the final estimated regularised solution for the first fitting data set.

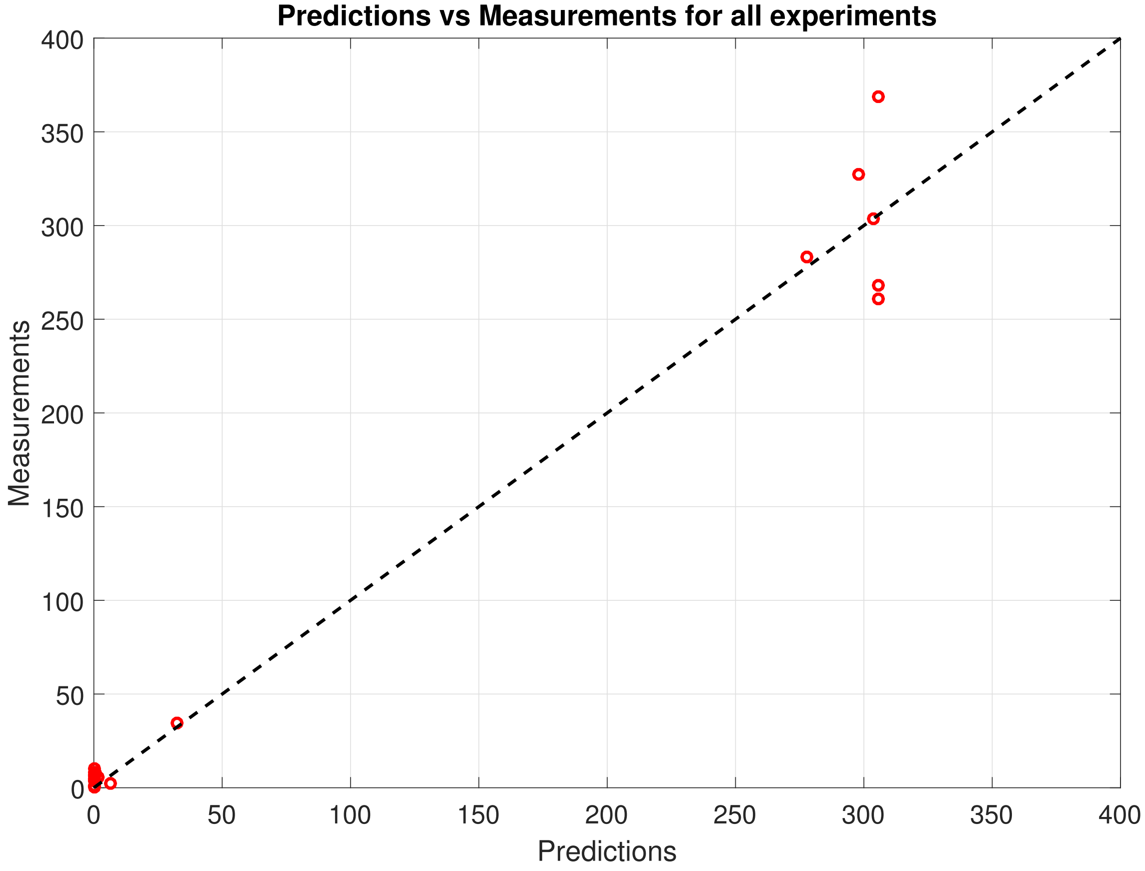

Figure S3.19: RP case study: predicted versus measured values for the first fitting data set.

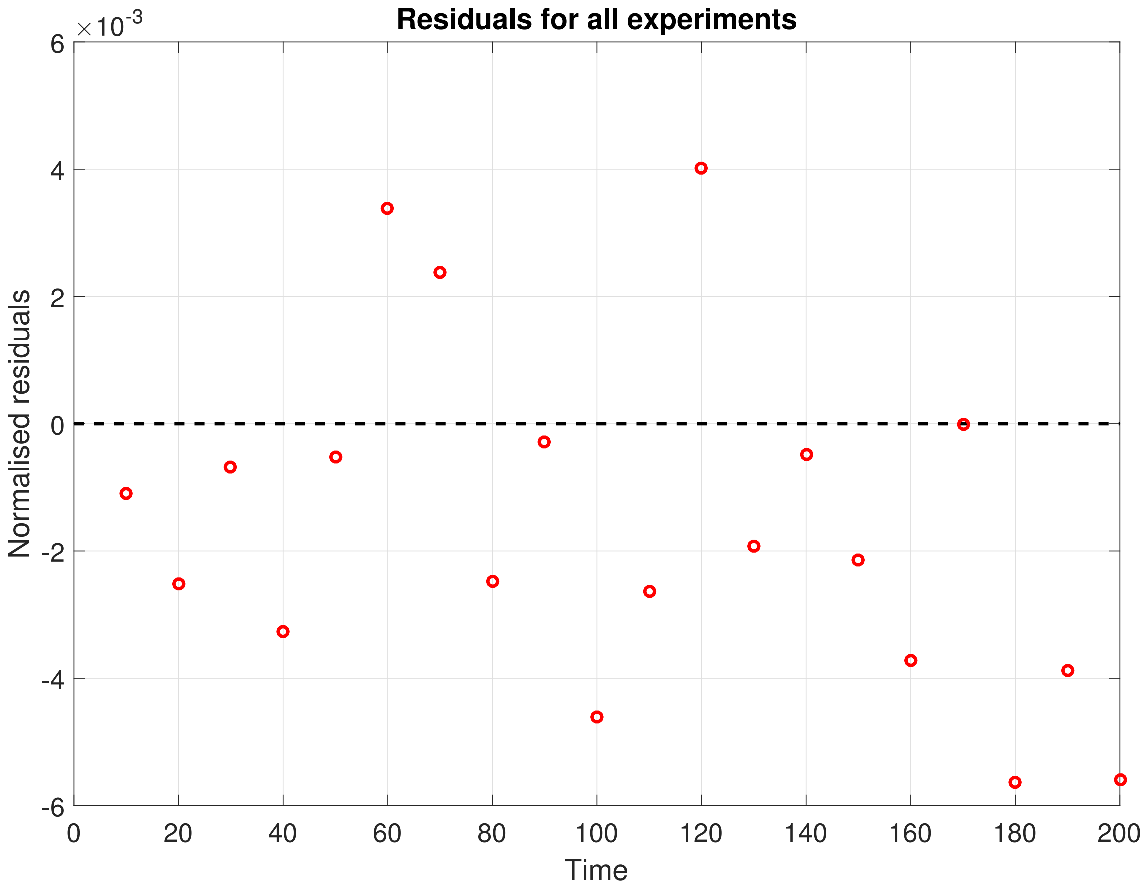

Figure S3.20: RP case study: normalised residuals for the regularised fit for the first fitting data set.

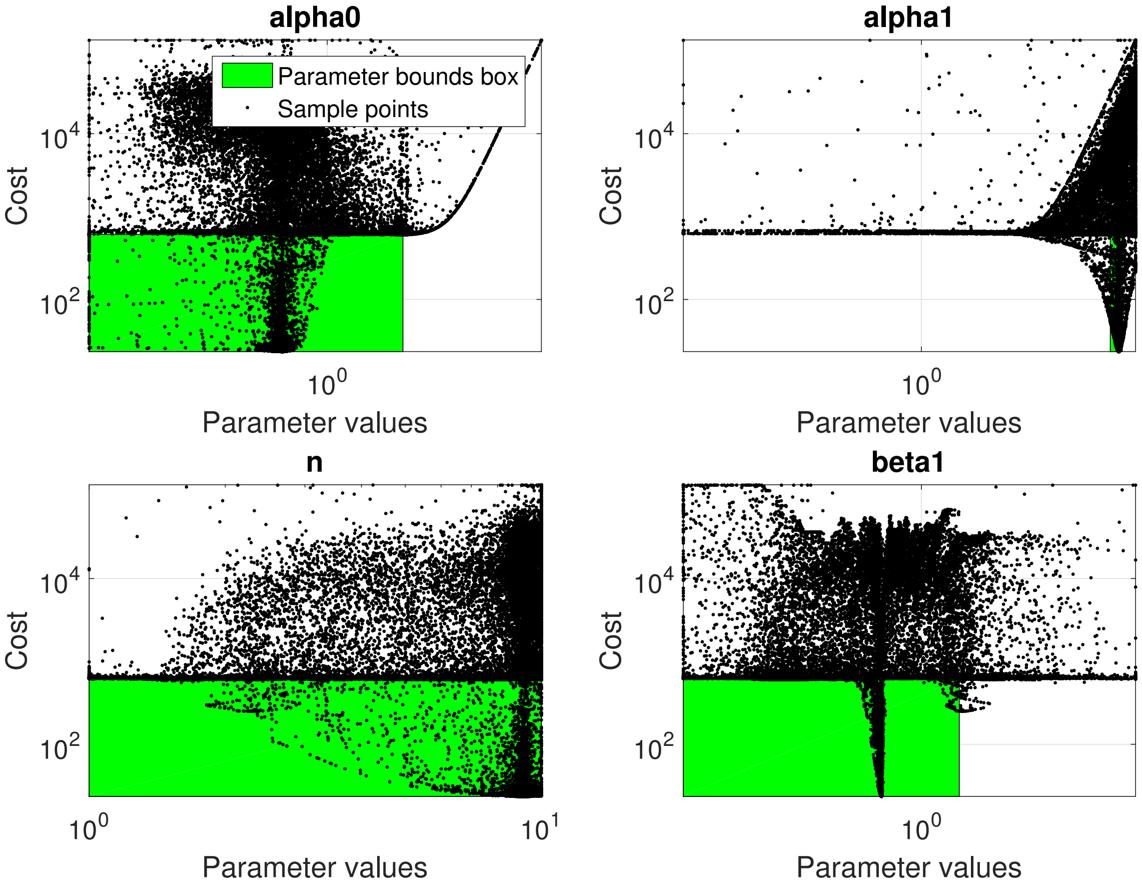

Figure S3.21: RP case study: sample from the initial estimation with the new parameter bound box, where the height of said box is the cost cut off for the first fitting data set.

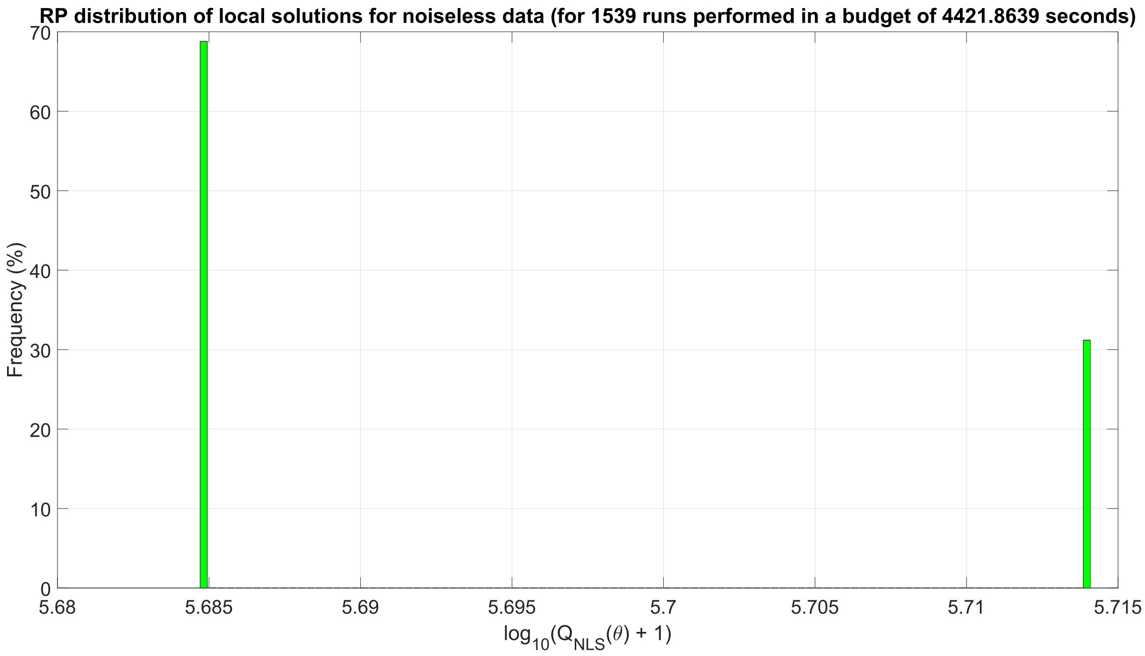

Figure S3.22: RP case study: distribution of solutions found using the nl2sol local solver fitting to noiseless data.

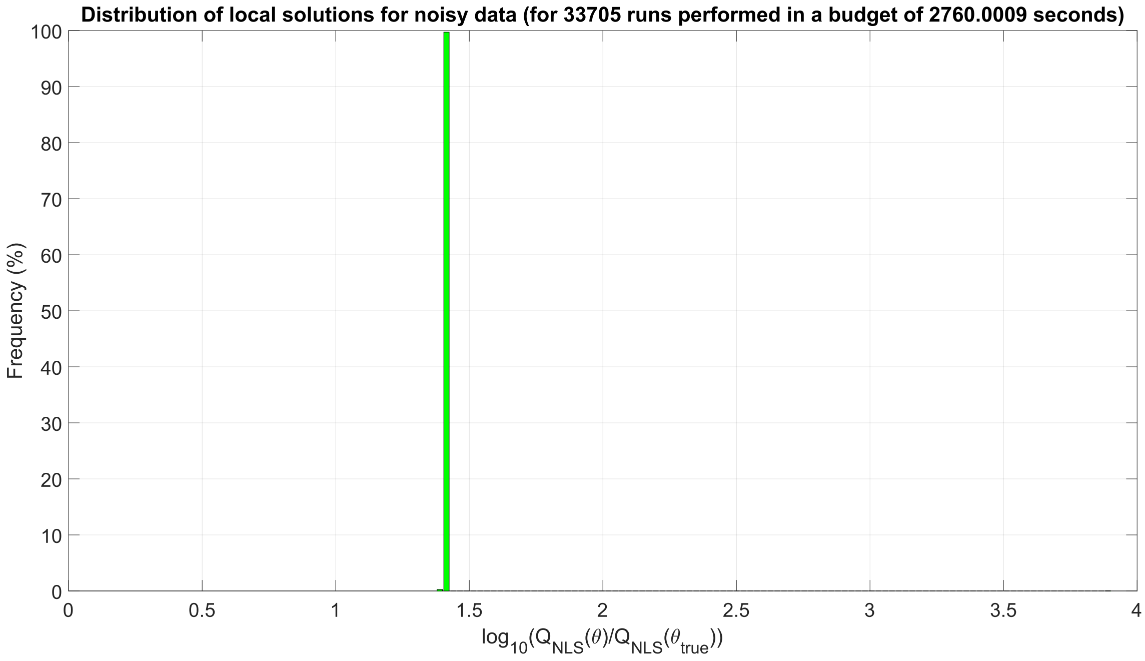

Figure S3.23: RP case study: distribution of solutions found using the nl2sol local solver fitting to noisy data for the first fitting data set.

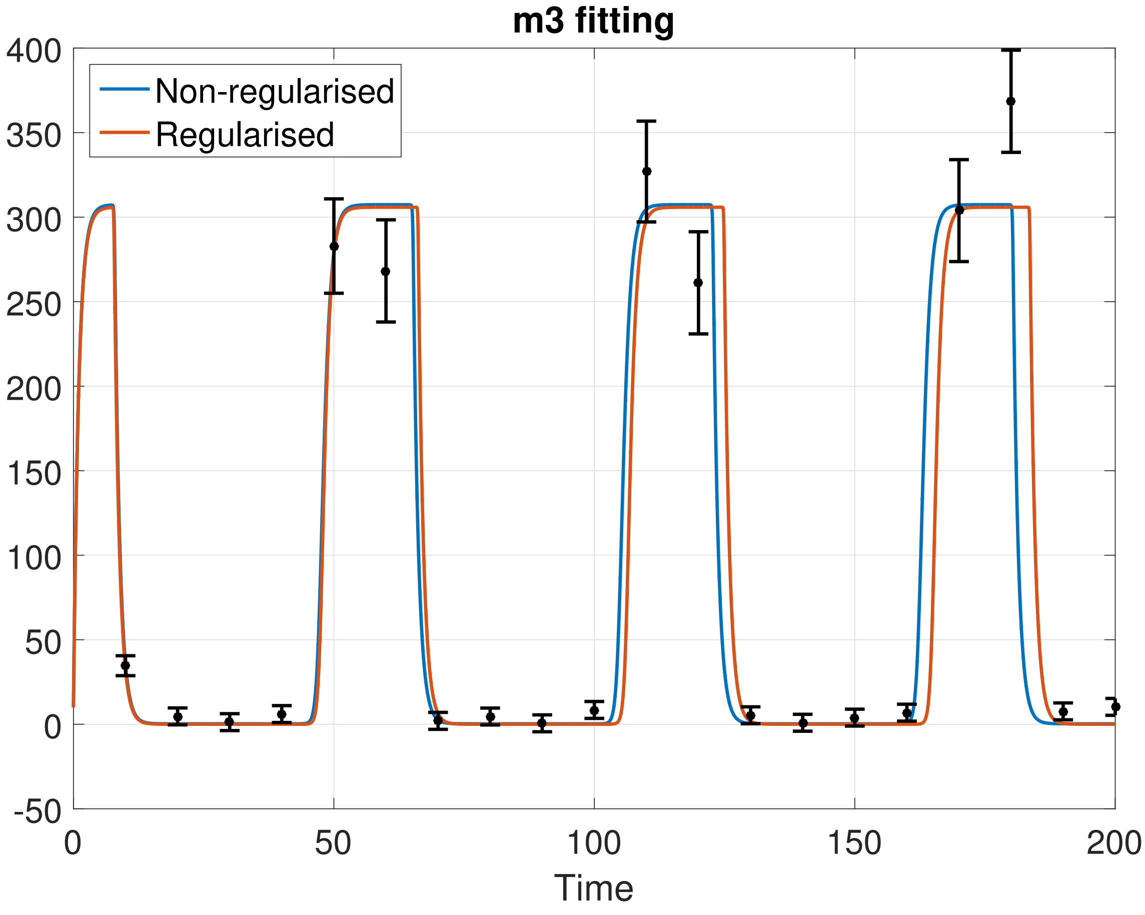

Figure S3.24: RP case study: comparison of the fits with and without regularisation for the first fitting data set.

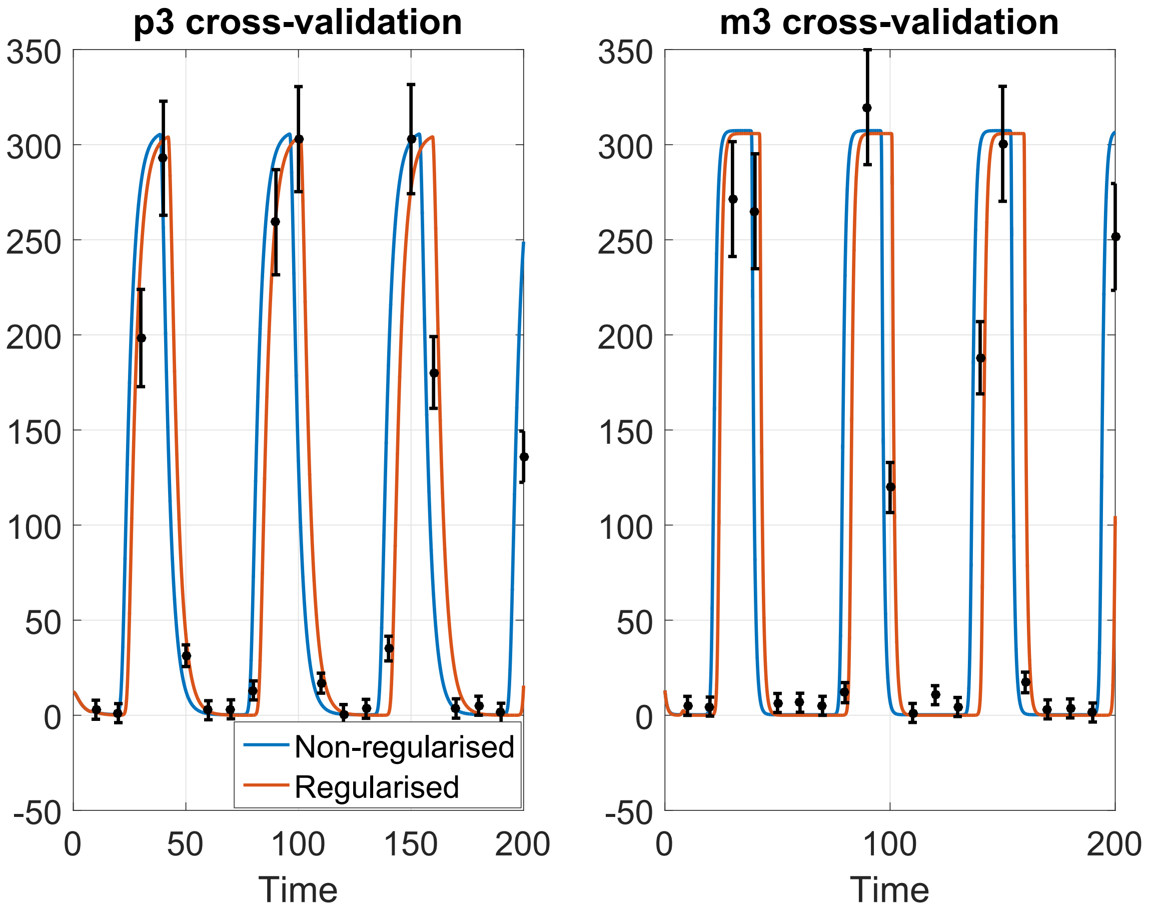

Figure S3.25: RP case study: comparison of the cross-validation with and without regularisation for the first fitting data set.

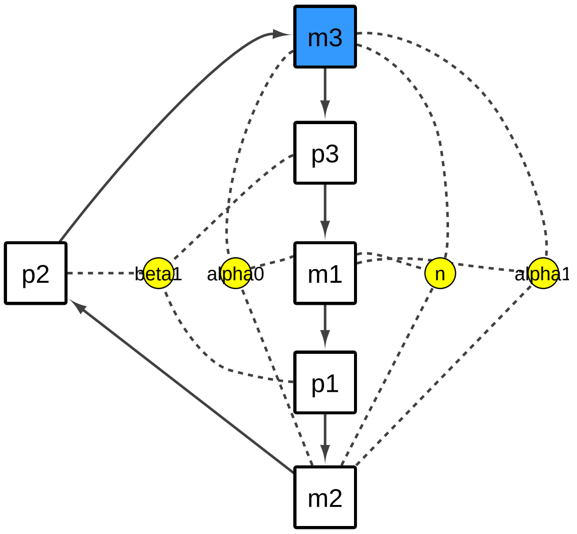

Figure S3.26: RP case study: comparison of the cross-validation with and without regularisation for the first fitting data set.

S3.3 EO problem results

Detailed results for the EO case study can be found here.

Table S3.9: EO case study: summary of the regularised results for the regularised fit to the first fitting experiment.

Parameter	Value	Confidence (95%)	Coeff of variation (%)	Bounds status
$v_{Km1_{r1}}$	0.3691	± 0.0401572	5.55112	Bounds not active
$L1_{r2}$	14.2721	± 44.3378	158.4996	Bounds not active
σ_{r2}	1.3350	± 0.886679	33.88626	Bounds not active
$L2_{r3}$	1.9281	± 13.7542	363.9648	Bounds not active
d_{r3}	7.9994	± 2.9484	18.80504	Bounds not active
$\sigma_{2_{r3}}$	6	± 5.15811	43.86151	Bounds not active
ks_{r4}	2.1013	± 1.65248	40.12207	Bounds not active

Table S3.10: EO case study: NRMSE values for the fitting for each fitting data set, with and without regularisation.

	Regularised	Non-regularised
Fitting set 1	0.01618	0.015716
Fitting set 2	0.007837	0.0077182
Fitting set 3	0.0081427	0.007838
Fitting set 4	0.010618	0.0045591
Fitting set 5	0.0054577	0.0042112
Fitting set 6	0.010091	0.0084268
Fitting set 7	0.043215	0.037391
Fitting set 8	0.021049	0.0088273
Fitting set 9	0.025143	0.020857
Fitting set 10	0.0088735	0.0064687

Table S3.11: EO case study: NRMSE values for the cross-validation for each regularised fit to the fitting data. Here, CV denotes cross-validation data set and F denotes fitting data set.

	F 1	F 2	F 3	F 4	F 5	F 6	F 7	F 8	F 9	F 10
All CV	0.075949	0.041773	0.074722	0.11311	0.2159	0.16357	0.048767	0.069949	0.07087	0.091781
CV 1	0.010495	0.0073621	0.010288	0.055885	0.063361	0.011585	0.031522	0.038265	0.033157	0.03294
CV 2	0.19835	0.076991	0.15847	0.14053	0.34867	0.34051	0.064481	0.14309	0.18031	0.23738
CV 3	0.024179	0.047758	0.023027	0.023362	0.1378	0.047815	0.037918	0.047011	0.0478	0.048011
CV 4	0.012877	0.0095845	0.0096689	0.016139	0.0085784	0.010928	0.036482	0.04508	0.024547	0.030581
CV 5	0.015042	0.012526	0.015787	0.054148	0.064641	0.013926	0.031876	0.04334	0.033136	0.032888
CV 6	0.011184	0.0223	0.020721	0.023283	0.067129	0.023689	0.035123	0.019457	0.028716	0.022605
CV 7	0.11098	0.048859	0.036935	0.083606	0.15534	0.16512	0.041104	0.047357	0.063121	0.1218
CV 8	0.066309	0.067321	0.16432	0.28146	0.51942	0.34567	0.098093	0.12307	0.067199	0.067379
CV 9	0.01584	0.014522	0.014766	0.059726	0.072066	0.016461	0.03176	0.042572	0.034571	0.035993
CV 10	0.012739	0.036204	0.027003	0.10492	0.11695	0.036063	0.035935	0.035893	0.045926	0.036079

Table S3.12: EO case study: NRMSE values for the cross-validation for each non-regularised fit to the fitting data. Here, CV denotes cross-validation data set and F denotes fitting data set.

	F 1	F 2	F 3	F 4	F 5	F 6	F 7	F 8	F 9	F 10
All CV	0.12834	0.040675	0.068498	0.17703	0.23943	0.19182	0.048399	0.086123	0.10175	0.13844
CV 1	0.0078941	0.0076341	0.0082981	0.062903	0.0068656	0.065486	0.033209	0.032536	0.038088	0.054944
CV 2	0.22338	0.077549	0.14615	0.25931	0.46443	0.30199	0.078843	0.20939	0.1598	0.16492
CV 3	0.072992	0.047717	0.075537	0.046739	0.11988	0.11796	0.048357	0.13418	0.041548	0.026543
CV 4	0.0103	0.010541	0.0092106	0.0087533	0.019601	0.0097001	0.040514	0.0096242	0.026578	0.032316
CV 5	0.011825	0.012733	0.012482	0.061876	0.012746	0.070974	0.034511	0.033156	0.036845	0.055268
CV 6	0.012605	0.022573	0.022117	0.067082	0.014338	0.059174	0.0408	0.022621	0.030608	0.0088072
CV 7	0.020195	0.048913	0.03302	0.11964	0.022308	0.12665	0.050023	0.049515	0.07295	0.081966
CV 8	0.32878	0.067523	0.13123	0.43853	0.5843	0.46659	0.064643	0.067231	0.24315	0.3618
CV 9	0.013482	0.014178	0.014267	0.094821	0.01336	0.070209	0.035888	0.035598	0.050558	0.085043
CV 10	0.018098	0.016118	0.02321	0.12639	0.018409	0.10603	0.036072	0.036347	0.069487	0.10817

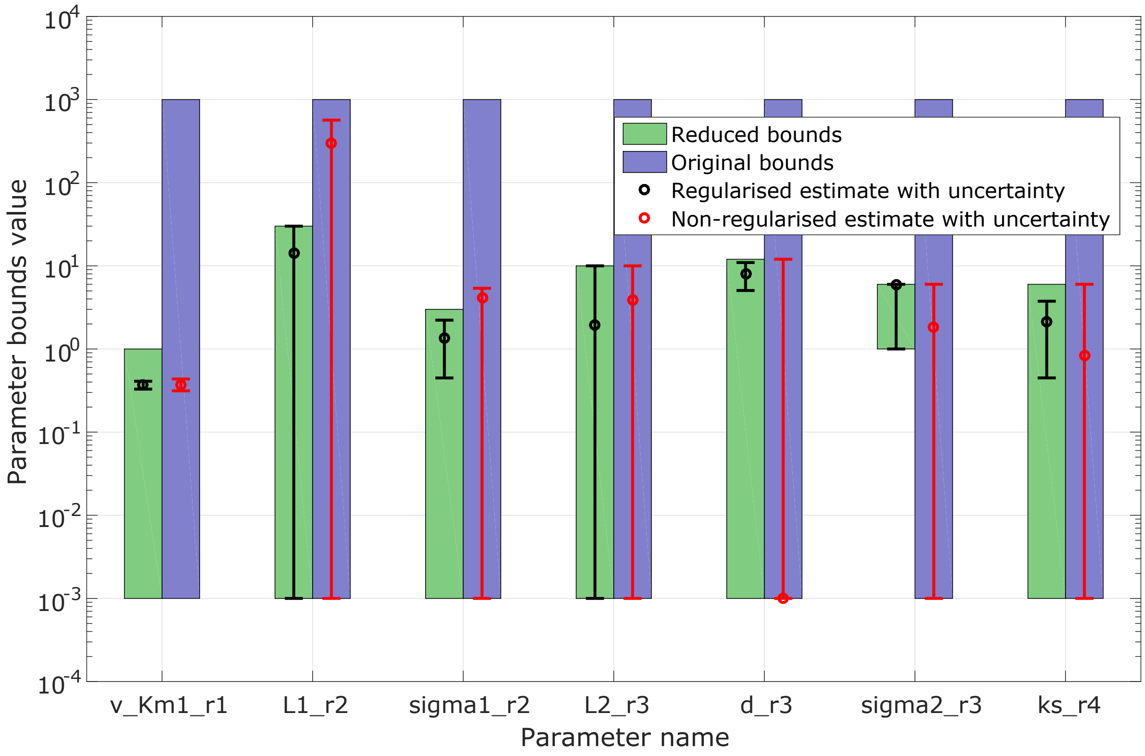

Figure S3.27: EO case study: reduction in the parameter bounds with the estimated values and their 95% confidence intervals for the first fitting data set.

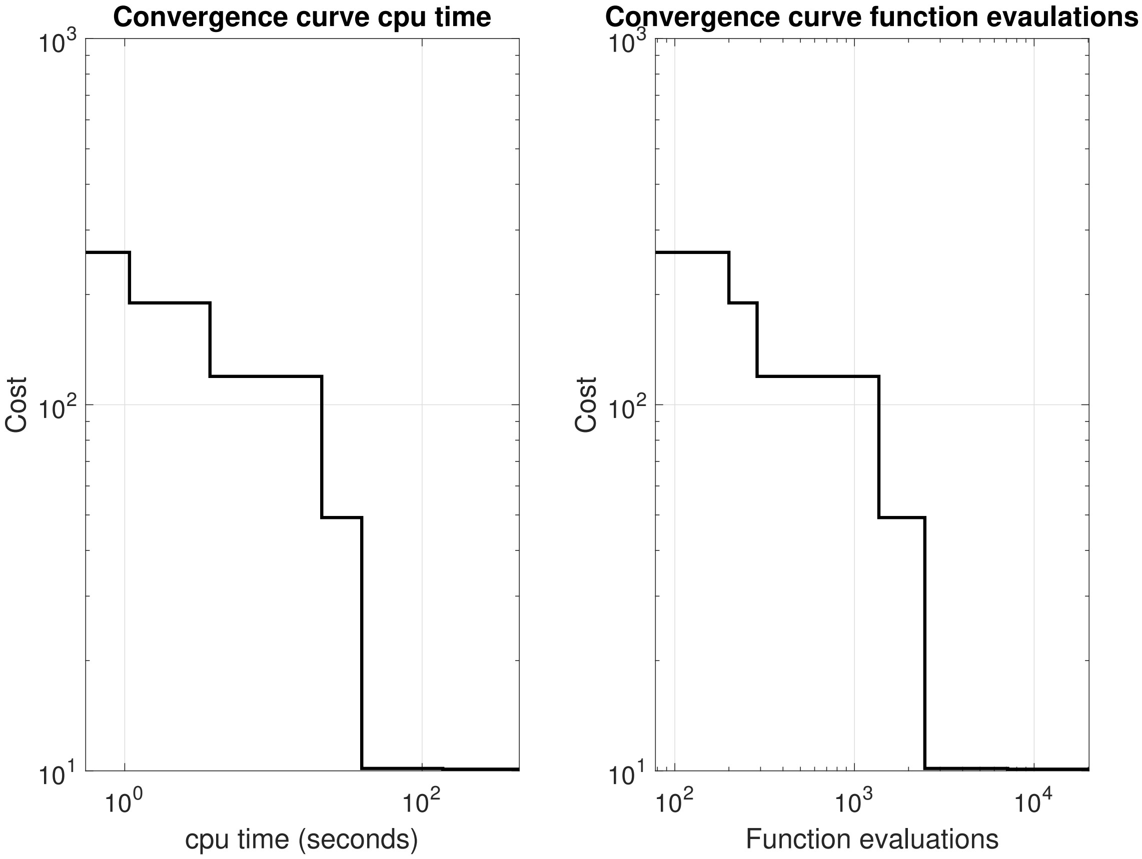

Figure S3.28: EO case study: convergence curve of the final regularised estimation for the first fitting data set.

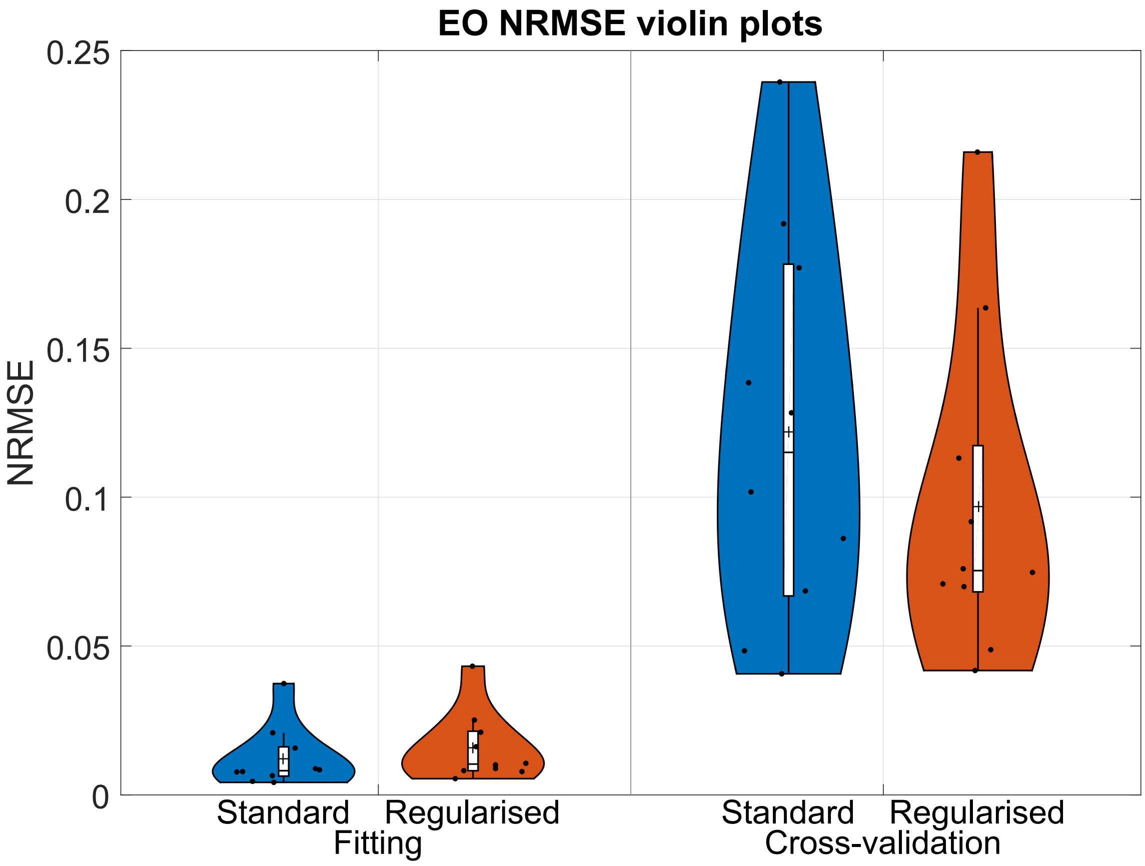

Figure S3.29: EO case study: violin plots showing the distribution of the NRMSE for the fit and cross-validation for all the data sets considered, both with and without regularisation.

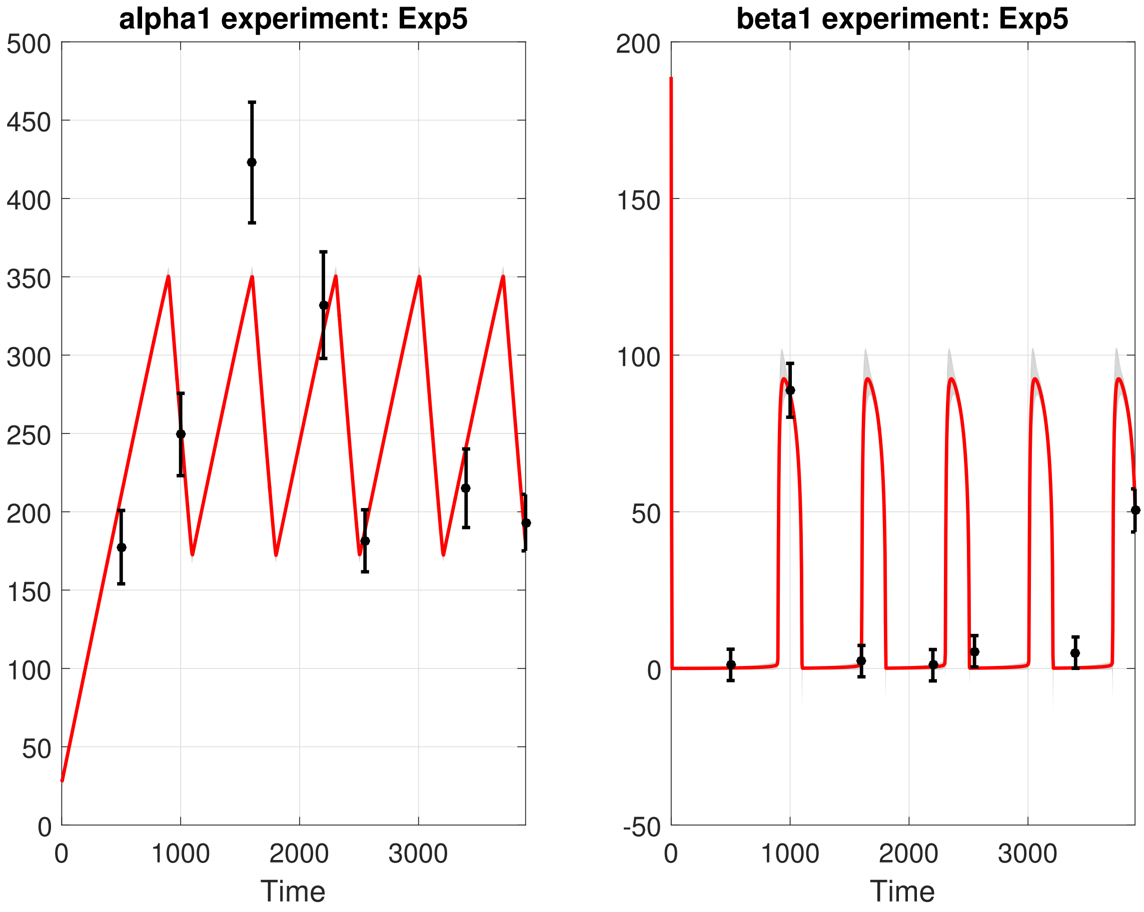

Figure S3.30: EO case study: final regularised fit with uncertainty intervals for the first fitting data set.

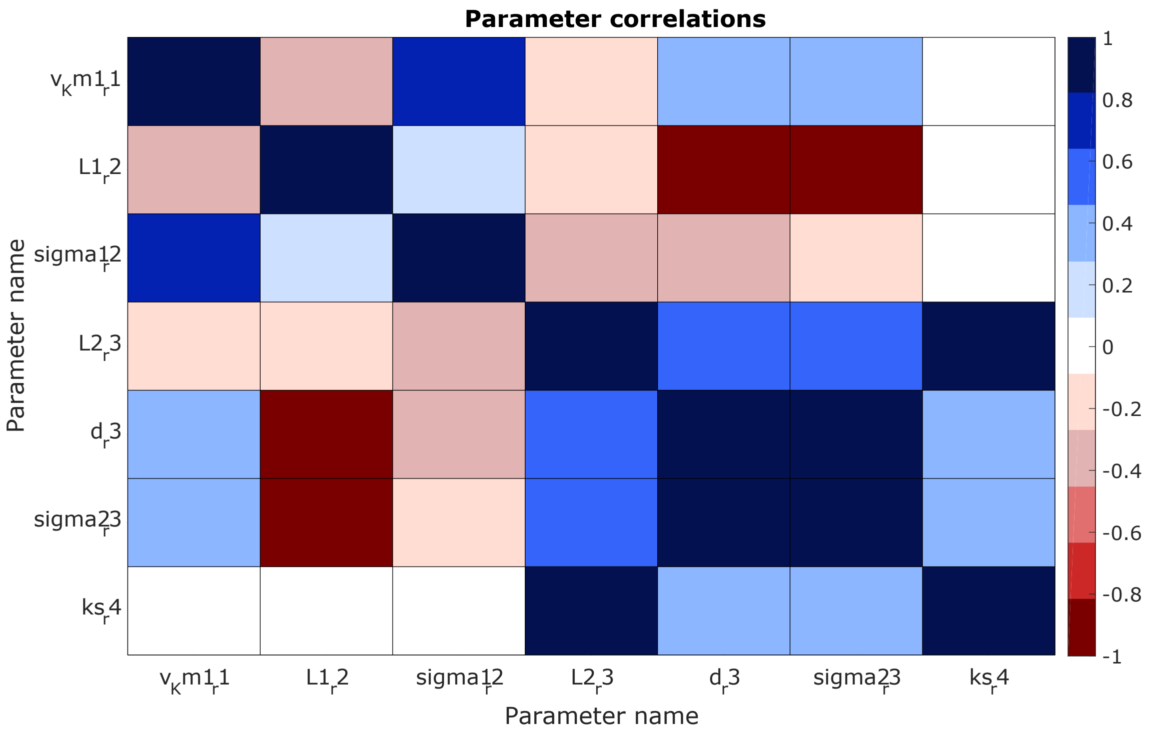

Figure S3.31: EO case study: final regularised fit with uncertainty intervals for the first fitting data set.

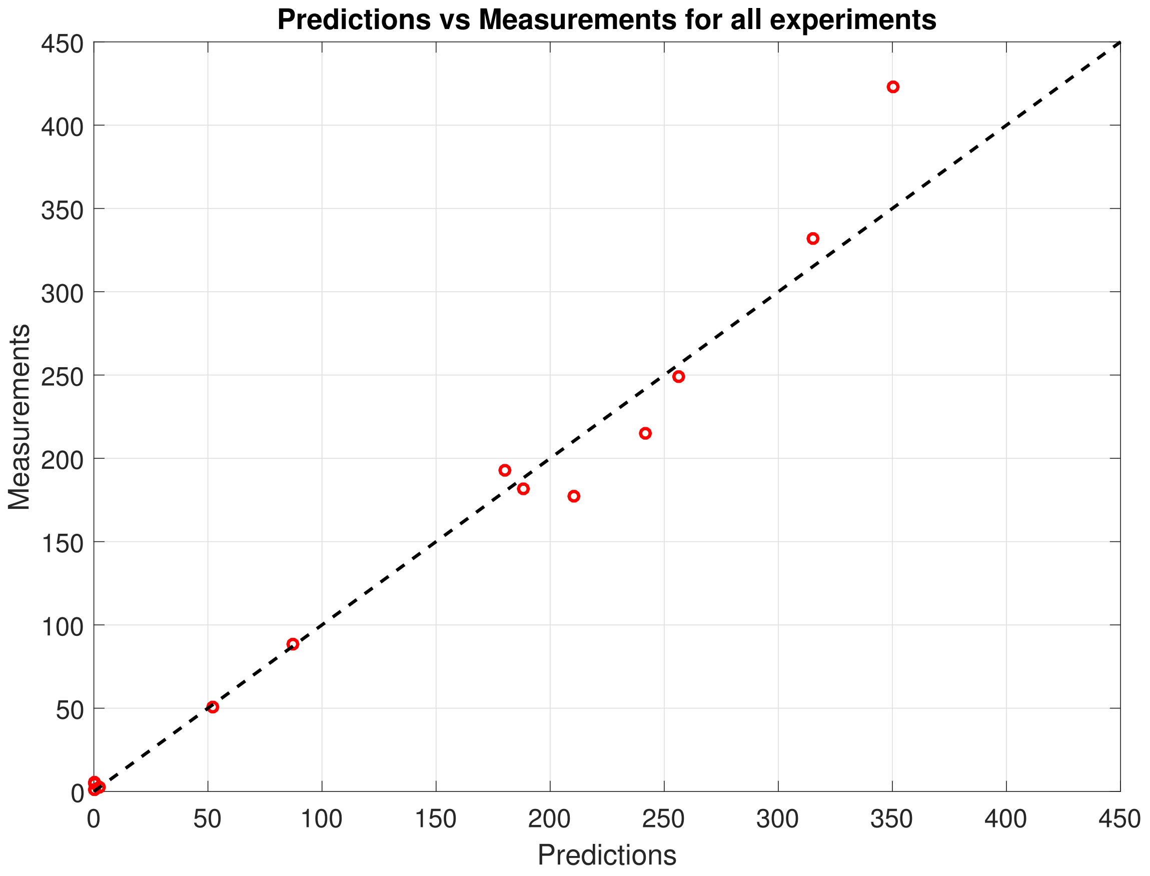

Figure S3.32: EO case study: predicted versus measured values for the first fitting data set.

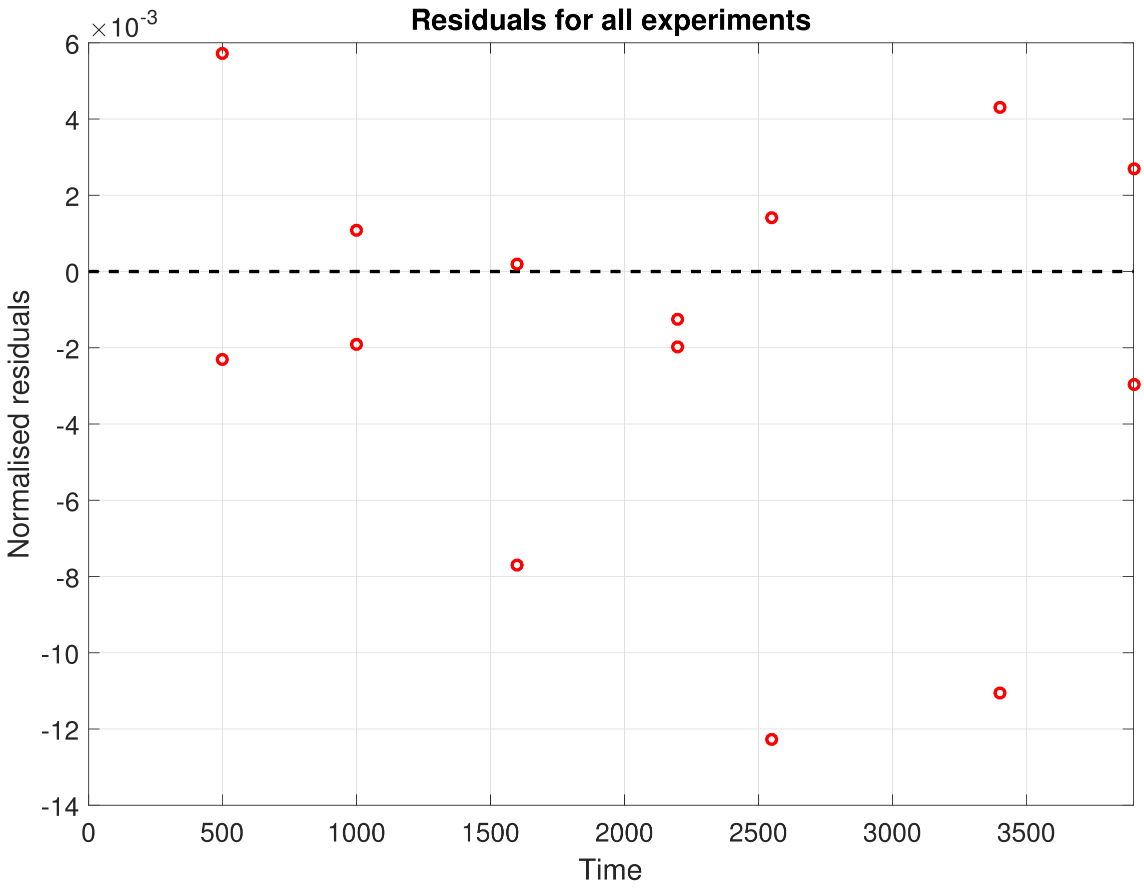

Figure S3.33: EO case study: normalised residuals for the regularised fit for the first fitting data set.

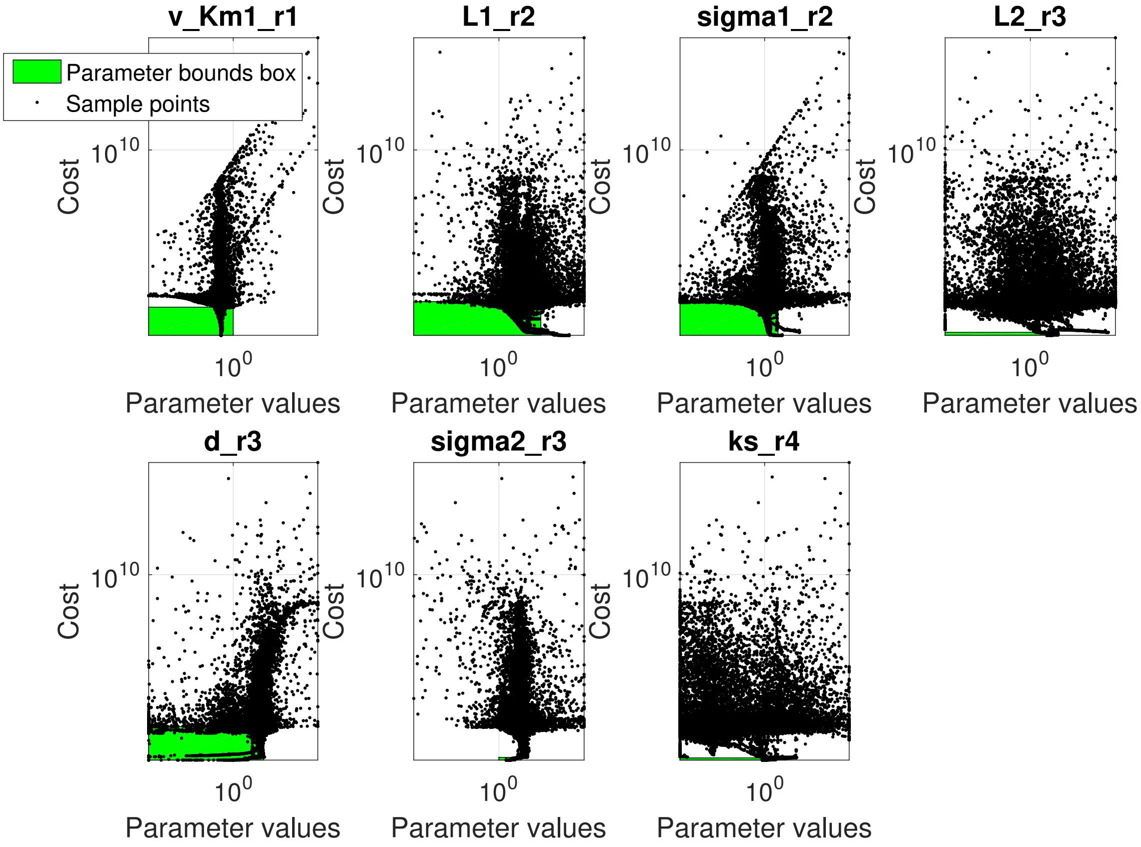

Figure S3.34: EO case study: sample from the initial estimation with the new parameter bound box, where the height of said box is the cost cut off for the first fitting data set.

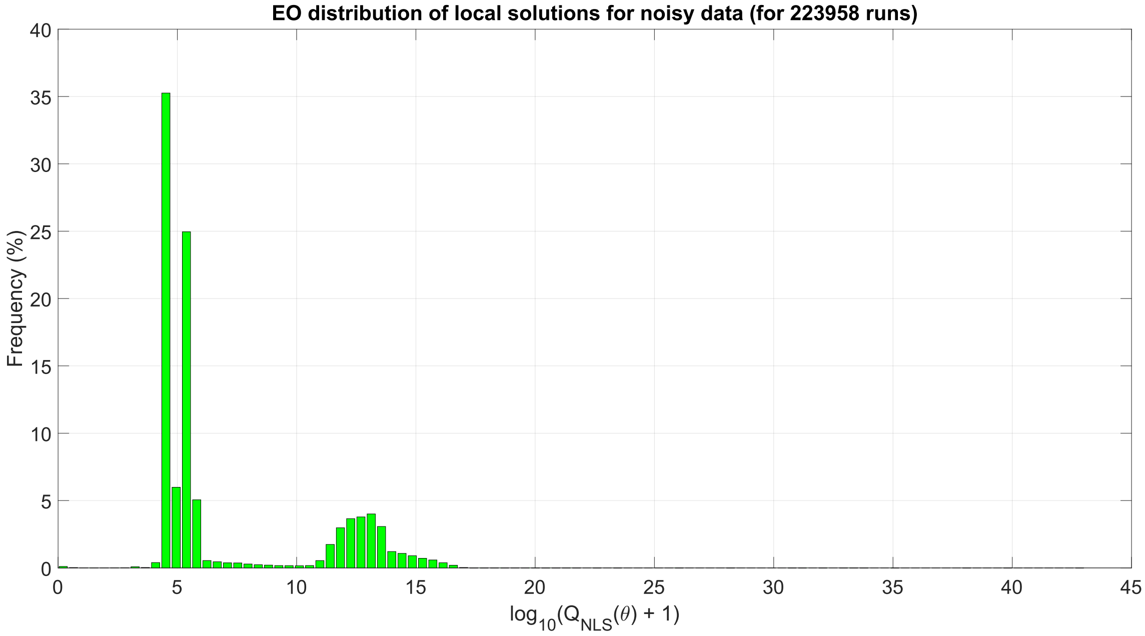

Figure S3.35: EO case study: distribution of solutions found using the nl2sol local solver fitting to noiseless data.

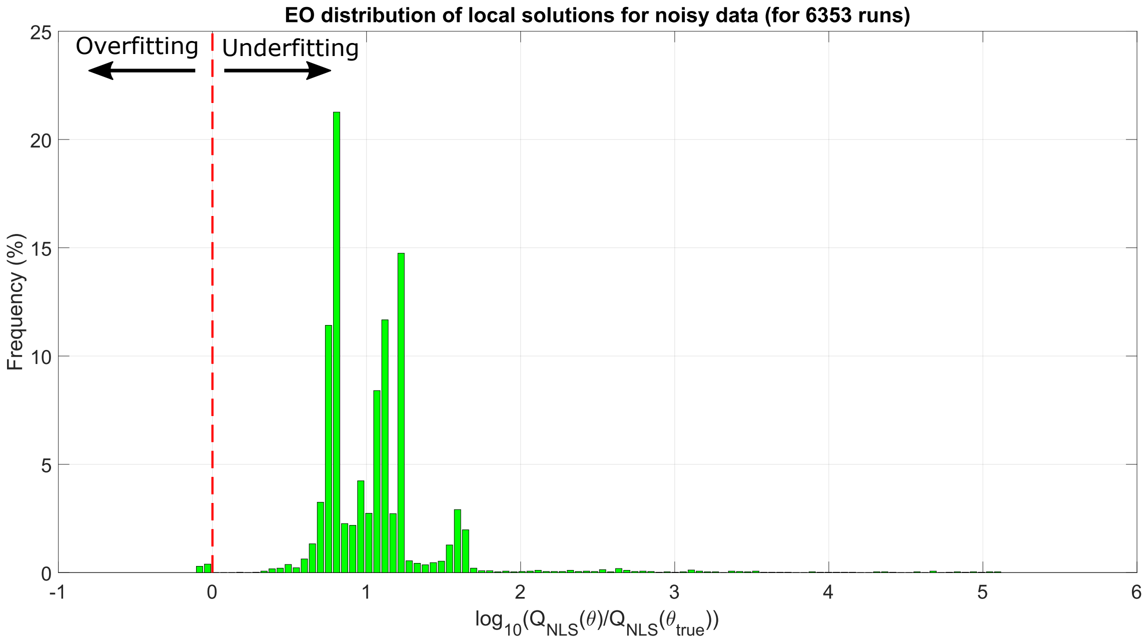

Figure S3.36: EO case study: distribution of solutions found using the nl2sol local solver fitting to noisy data for the first fitting data set.

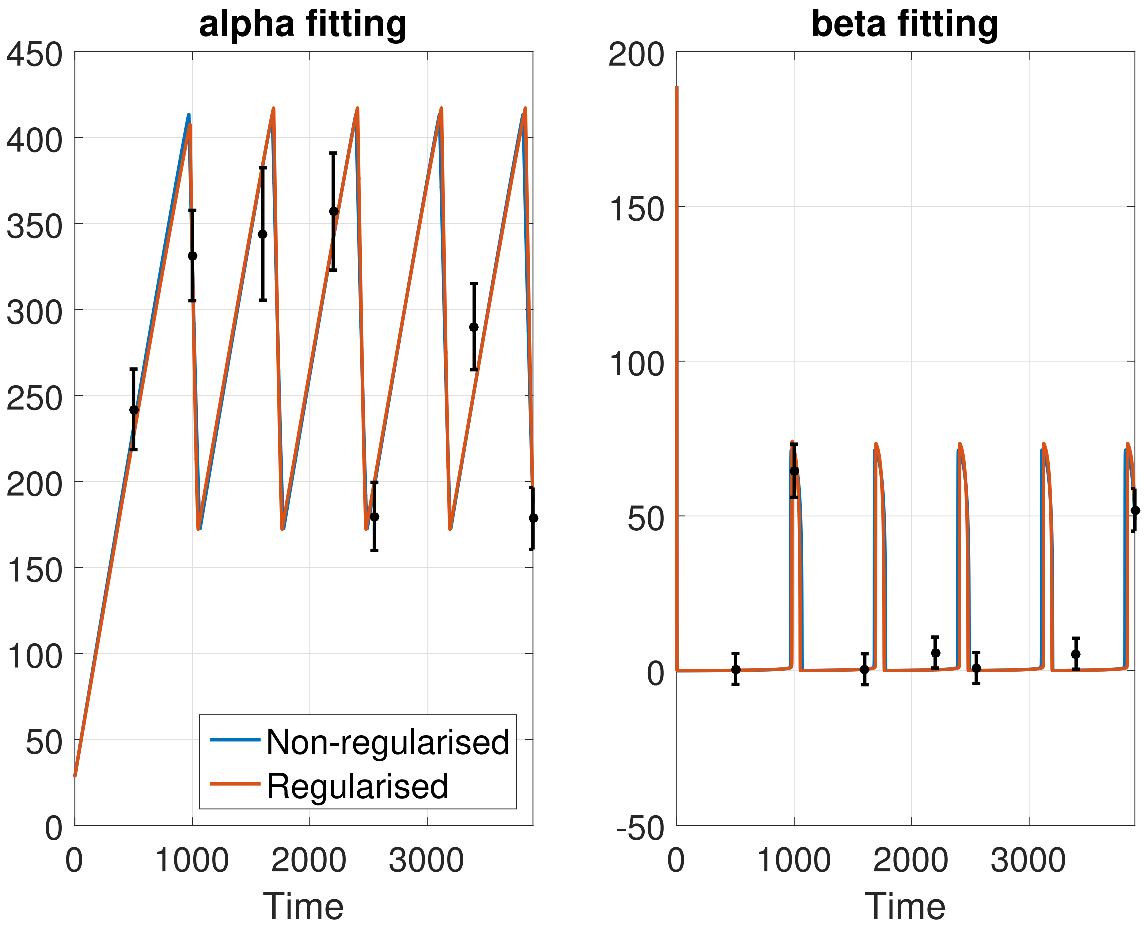

Figure S3.37: EO case study: comparison of the fits with and without regularisation for the first fitting data set.

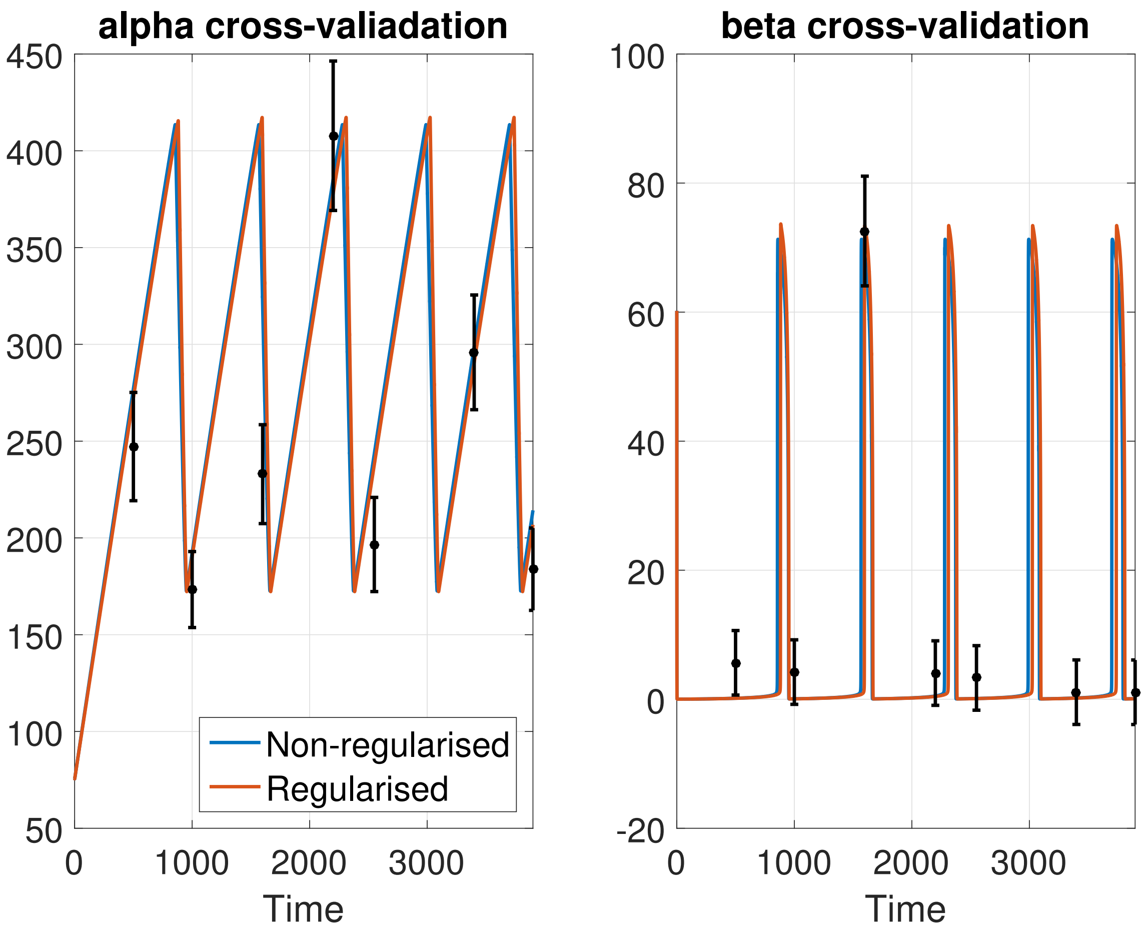

Figure S3.38: EO case study: comparison of the cross-validation with and without regularisation for the fit to the first fitting data set.

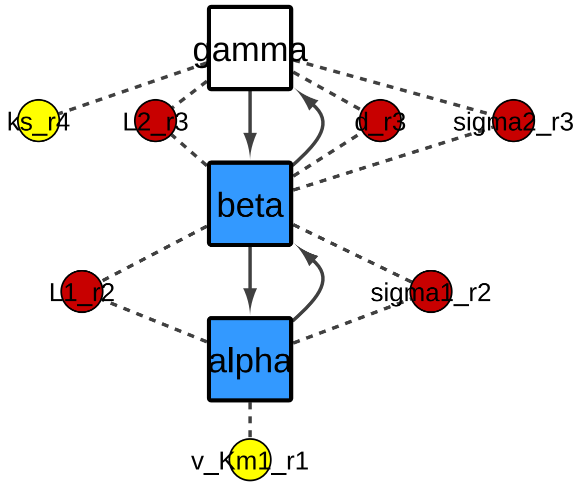

Figure S3.39: EO case study: results of the VisId analysis performed at the regularised solution for the first fitting data set.
